# Bridging training and practice gap: A mixed methods tracer study of bachelor of science in nursing graduates (2016–2020) at Kairuki University, Dar es Salaam, Tanzania

**DOI:** 10.1371/journal.pone.0333702

**Published:** 2025-10-03

**Authors:** Adellah Sariah, Minael Nathanael, Monica Bugomola, Edson Sungwa, Mathew Ndomondo, Elizabeth Mika, Tausi Haruna, Joan Zenas, Ezekiel Mbao, Innocent Semali, Columba Mbekenga

**Affiliations:** 1 Department of Mental Health and Psychiatric Nursing, Kairuki University, Dar es Salaam, Tanzania; 2 Department of Community Health Nursing, Kairuki University, Dar es Salaam, Tanzania; 3 Department of Reproductive and Child Health, Kairuki University, Dar es Salaam, Tanzania; 4 Department of Fundamentals of Nursing and Basic Sciences, Kairuki University, Dar es Salaam, Tanzania; 5 Department of Medical and Surgical Nursing, Kairuki University, Dar es Salaam, Tanzania; 6 Department of Community Medicine, Kairuki University, Dar es Salaam, Tanzania; IMU: International Medical University, MALAYSIA

## Abstract

**Background:**

Tracer studies evaluate the effectiveness of university training by assessing how graduates perform in the job market. This study focused on Bachelor of Science in Nursing (BScN) graduates, aiming to describe their training experiences, application of acquired competencies, and overall stakeholder perceptions to inform BScN curriculum improvement.

**Methods:**

A convergent parallel mixed-method design was used to collect quantitative and qualitative data concurrently from 2016–2020 BScN graduates (February-May 2023). Graduates and other stakeholders (including educators, employers, and policymakers) in this study were selected from private, public, and faith-based hospitals and universities, colleges, and the Ministry of Health. Quantitative data were gathered via online structured questionnaires adapted and modified from the American International Health Alliance and the Technical Vocational Education and Training tools. Qualitative data were collected through interviews and focus groups with graduates, employers, educators, and policymakers. Quantitative data were analyzed using descriptive statistics, while qualitative data underwent thematic analysis. Integration occurred during interpretation to provide a comprehensive understanding of graduates’ experiences, competency application, and stakeholder perceptions of the BScN program.

**Results:**

Among the 61 graduates who completed the online survey, 37 (60.7%) were female. Most (48; 78.7%) worked as nurses, while 6 (9.8%) were tutors and 3 (4.9%) worked as tutorial assistants. Demonstration was rated the most useful teaching and learning method by 52 (85.2%) respondents, and 47 (81%) rated practical exams as a useful assessment method. These were also supported by graduates and stakeholders who shared their perspectives with regard to the benefits and impact of the BScN program and training quality. Additionally, 54 graduates (94.7%) found the program very useful in preparing them for their professional roles, which aligned with their views on the connection between acquired competencies and job performance. Both graduates and educators highlighted challenges encountered during training and in professional practice. Policymakers and graduates also offered recommendations for improving the program.

Additionally, 54 graduates (94.7%) found the program very useful in preparing them for their professional roles, which aligned with their views on the connection between acquired competencies and job performance. Both graduates and educators highlighted challenges encountered during training and in professional practice. Policymakers and graduates also offered recommendations for improving the program.

**Conclusion:**

The findings demonstrate that the BScN program is widely regarded by graduates and stakeholders as effective in preparing students for professional practice, particularly through practical teaching methods such as demonstrations and practical exams. While the program’s impact on competency development and job performance was strongly affirmed, the study also revealed notable challenges during training and practice. These insights support the ongoing review and enhancement of the BScN curriculum.

## Introduction

Tracer studies are an effective tool for evaluating the outcomes of the education and training provided to graduates of a given institution, particularly in relation to the labor market and employer expectations [1]. They serve as an essential component of quality assurance, offering critical insights that inform curricula reviews and help enhance the quality of university education [2]. These studies aim to assess the medium- to long-term impact of teaching and learning by examining how graduates perform and transition into the job market [1,3]. As such, universities worldwide conduct tracer studies to evaluate the effectiveness of academic programs in preparing students for employment [4]. They gather information on the relevance of education and training in meeting labor market demands [1,3], providing valuable feedback for graduates, current students, employers, policymakers, and academic curricula [1,3]. Tracer studies typically involve identifying and following up with graduates from Higher Education Institutions (HEIs) to collect information about their training experiences and career transitions [1,3].

Tracer studies aim to identify challenges encountered during training and after graduation; improve training delivery models, curriculum content, and study conditions; and offer strategies to enhance graduates’ transition from education to the labor market. They also help align graduates’ competencies with current market demands [3]. As such, studies provide valuable information for evaluating the status and performance of graduates [1,3] by gathering feedback from stakeholders, such as graduates, educators, employers, NGOs, and the general public. These studies help identify gaps and inform effective curriculum reviews [5,6]. If tracer studies are not conducted regularly, HEIs may be unable to identify the strengths or weaknesses of their graduates, understand graduates’ perceptions of the program, or assess the evolving demands of the labor market. As a result, graduates may be inadequately prepared to meet societal needs. Additionally, the lack of tracer studies leads to the continued use of outdated curricula, making it difficult for HEIs to make evidence-based decisions to enhance the quality of education and services for nursing students [7].

The major role of HEIs is to ensure that the training offered equips nursing students with knowledge, attitude, and skills, as well as enabling them to adapt to rapidly changing labor market requirements and conditions. Regular, timely curriculum reviews informed by appropriate tracer studies provide an opportunity for educators to keep abreast with the ongoing socio-economic and technological changes, emerging and re-emerging diseases, and increased mobility by updating the curricula to reflect the current societal needs. For example, with the modern advancements in technology and internet use, HEIs and other training institutions are implicitly obliged to transform their teaching and learning methods to meet the educational opportunities for all [8]. This can be achieved through designing curricula that are informed by tracer studies, ensuring that learners are well-trained and have the necessary competencies required in the labor market. Reports from other nursing graduate tracer studies from the Philippines show how the Bachelor of Science in Nursing (BScN) program equips graduates with essential skills, leading to broader and more relevant employment opportunities within a short time frame. For instance, studies have shown that more than half of the graduate nurses secured employment within six months of graduation [9], and over two-thirds were employed in roles directly related to their degrees [9,10]. Moreover, another study found that two-thirds of the competencies demonstrated by graduate nurses were acquired through workplace experience, while the remaining one-third were attributed to their BScN training [11]. Graduates reported that the nursing degree provided them with professional competencies [9] such as communication skills, critical thinking, human relations [10], clinical performance, documentation, and a comprehensive understanding of nursing practice [11], as well as effective interpersonal relationship skills [12]. Although some nurses reported employment in non-professional fields, the findings suggest that nursing education should also prepare graduates to pursue alternative opportunities, including entrepreneurship and self-employment.

Notably, graduate nurses expressed that the program was useful in their current positions, allowing them to apply what they learned [9], which in turn enhanced their motivation and performance [12]. Graduates also rated the program highly in terms of its impact on both their personal and professional development. Key strengths of the program included the relevance of the curriculum to professional demands, its emphasis on problem-solving, quality instruction, strong teacher-student relationships, and institutional infrastructure [9]. Furthermore, employers observed significant improvements in graduates’ workplace attitudes as evidenced by reduced absenteeism, fewer complaints, increased punctuality, fewer work-related accidents, and reduced use of mobile phones during work hours [11].

Kairuki University (KU), formerly known as Hubert Kairuki Memorial University (HKMU), was founded in 1997 and was originally established as the Mikocheni International University of Health Sciences (MIUHS). It was later rebranded as Mikocheni International University (MIU). The university was renamed HKMU in honor of its founder, the late Prof. Hubert C.M. Kairuki. In April 2024, the name officially changed from HKMU to KU in April 2024. The university comprises the Schools of Nursing, Medicine (previously known as Faculties), Social Work (a former department), as well as the Institute of Postgraduate Studies and Research. It offers both undergraduate degree programs in Medicine, Nursing, and Social work as well as Postgraduate programs, i.e., Master of Science in Public Health (MScPH), and Master of Medicine (MMed) in Surgery, Pediatrics, Obstetrics and Gynaecology, and Internal Medicine. For clarity in this study, it is important to note that data collection, analysis, and reporting were completed before the change of the university name. As a result, the authors have retained the original name “HKMU” throughout the report to reflect the context in which the research was conducted.

The School of Nursing offers a Bachelor of Science in Nursing (BScN) program. This is a 4-year 8-semester program, followed by one year of compulsory rotatory internship at an approved hospital. The school comprises five departments, namely, the Department of Medical and Surgical Nursing, the Department of Fundamentals of Nursing and Basic Sciences, the Department of Reproductive and Child Health Nursing, the Department of Mental Health and Psychiatric Nursing, and the Department of Community Health Nursing. During the first two years, BScN students are trained in basic sciences, including anatomy, physiology, microbiology, biochemistry, just to name a few, and principles of nursing. The third and fourth years focus more on the core nursing clinical courses, such as medical and surgical nursing, paediatric nursing, mental health and psychiatric nursing, and nursing leadership and management. These clinical courses include both a theoretical component, where students receive classroom instruction, and a clinical component, where students rotate through various clinical and community settings to acquire the necessary competencies. Students are also taught other core nonclinical courses, including community health nursing and research.

The theoretical courses in the BScN program are integrated with clinical rotations and fieldwork in hospital and community settings. These courses are designed to prepare student nurses for the demands of modern healthcare practice. The Tanzania Nurses and Midwives Council (TNMC) plays a vital role in ensuring that all nursing and Midwifery training institutions produce competent graduates capable of delivering consistent and high-quality healthcare services. Additionally, the Tanzania Commission for Universities (TCU) oversees quality assurance systems across universities, emphasizing the importance of an up-to-date curriculum that is informed by timely and appropriate tracer studies.

The current BScN curriculum developed by HKMU was last reviewed in 2014 and subsequently reorganized in 2018. Findings from a previous, unpublished internal tracer study at HKMU- conducted in support of the Doctor of Medicine (MD) curriculum review -included responses from both medical (87.65%) and nursing (12.5%) graduates. Approximately 90% (56 out of 61) of all respondents reported that their training was appropriate for their current roles. However, feedback from employers and supervisors identified notable gaps in nursing competencies, particularly in patient care and midwifery skills. They recommended increased supervision of students during clinical practice to better address these deficiencies.

The low response rate of BScN graduates (12.5%) in the previous tracer study underscores the importance of reaching a larger number of graduates to assess accurately the program’s adequacy and alignment with community and professional expectations. Furthermore, limited data on how well the BScN curriculum equips graduates with workplace competencies highlighted the necessity of the study. A curriculum review is therefore crucial to incorporate contemporary developments, including responses to emerging and re-emerging pandemics-such as COVID-19 and Monkeypox, the rising burden of non-communicable diseases (NCDs), and rapid technological advancements, which have driven the expansion of specialized healthcare in Tanzania. Timely and relevant tracer studies are essential for aligning the curriculum with the evolving job market and the needs of graduates, stakeholders, and healthcare consumers. Furthermore, adapting to technological innovations and socio-economic transformations is critical for enhancing university-level teaching, learning, and clinical practice.

While previous evaluations of nursing education programs have often relied on quantitative surveys to assess outcomes, they frequently overlook the contextual and experiential insights that qualitative data provide. To address this gap, a mixed methods approach was employed in this study to generate an understanding of both measurable outcomes and the underlying experiences and perceptions of graduates, educators, employers, and policymakers. The design was used to allow for a comprehensive evaluation of the BScN program by combining the strengths of quantitative and qualitative approaches. The quantitative component provided measurable data on graduates’ characteristics, competencies, and perceptions, while the qualitative component offered rich, contextual insights into stakeholder experiences, perceptions, and recommendations. This design enabled the integration of numerical trends and in-depth perspectives, providing a deeper understanding of program outcomes and informing recommendations for curriculum improvement.

The primary aim of this tracer study was to describe HKMU BScN graduates’ (2016–2020) sociodemographic characteristics, the application of acquired competencies in their current professional roles, and their experiences of the BScN program’s teaching, learning, and assessment approaches. In addition, we also explored the overall perception of the BScN program and graduates among key stakeholders (educators, employers, and policymakers). Information from this study will generate evidence to inform the ongoing review and enhancement of the BScN curriculum.

## Materials and methods

### Study design

**Fig 1:**
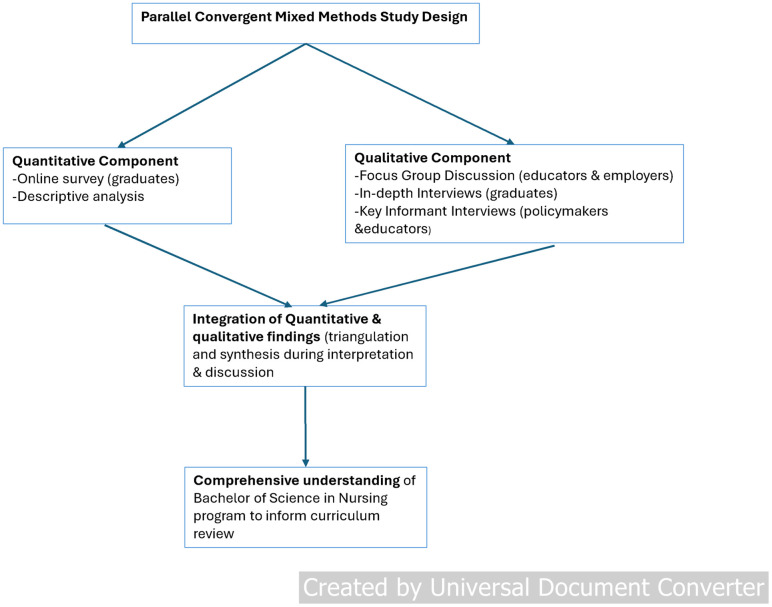
Study flow chart.

The study employed a Convergent Parallel Mixed-Methods Design, in which both quantitative and qualitative (QUAN+QUAL) data were collected separately but concurrently between February 2023 and May 2023. Quantitative and qualitative data were analyzed separately but concurrently, and the findings were compared. Triangulating data from both approaches enabled researchers to develop a comprehensive understanding of the phenomenon under study, with one set of findings confirming or complementing the other [13] (Fig 1). Using both methods concurrently allowed for the integration of subjective and objective perspectives, enriching the overall interpretation of the study’s context and results.

The research team comprised experienced faculty and researchers from nursing education and public health, with expertise in both qualitative and quantitative methods to ensure methodological rigor in the mixed-method tracer study. The Principal Investigator, a senior nursing educator with over 10 years of experience and a qualitative expert, led the study design, developed interview guides, oversaw thematic analysis, and ensured integration of both research approaches. A quantitative researcher and professor of epidemiology led the design and validation of the structured questionnaire, conducted statistical analysis using STATA, and interpreted the quantitative findings. The tracer study coordinator, also a nurse educator and qualitative expert, developed interview guides, trained the interviewers, oversaw field logistics and participant recruitment, and performed data analysis and reporting. Additional team members, with diverse research experience, scheduled interviews, and supported qualitative data collection, transcription, analysis, and reporting.

## Quantitative component

The quantitative component aimed to assess graduates’ social demographic characteristics, how graduates apply the acquired competencies in their current jobs, the usefulness of teaching, learning, and assessment methods, as well as the overall BScN program.

### Study settings

HKMU BScN Graduates were recruited from several facilities, including the Muhimbili National Hospital, regional and district hospitals, private and faith-based hospitals, higher learning institutions (both government and private), non-governmental organizations, and the Ministry of Health (MoH) in Tanzania. The majority were employed in these facilities as registered nurses, educators, researchers, or officers in the MoH.

### Study population

The study population included all BScN students who graduated from HKMU from 2016 to 2020. Participants were included in the study if they had graduated from HKMU in the past five years (from 2016 to 2020) and consented to participate in the study. They were excluded if they were mentally incompetent, severely ill, or could not be reached by any means possible.

### Sampling and recruitment of participants

The total number of HKMU BScN graduates from 2016–2020 was 182. Since the minimum calculated sample size was 125, which was close to the total BScN graduate population (182), all eligible graduates from the five cohorts (2016–2020) were invited to participate in the tracer study. From this, only 61 graduates responded to the invitation (response rate = 33.52%) (Table 1).

**Table 1:** Distribution of graduates, response, and response rate.

Graduation year	Number of graduates	Number of respondents	Response rate
**2016**	15	3	20%
**2017**	22	8	36.36%
**2018**	32	14	43.75%
**2019**	59	6	10.16%
**2020**	54	30	55.55%
**Total**	182	61	33.52%

Since the study included all eligible graduates from the finite population, no sampling technique was applied. Instead, the team employed recruitment procedures to engage the entire population. Five researchers from the tracer study team were responsible for tracking the 2016–2020 BScN graduates through the HKMU Alumni Association, the Dean of the Faculty of Nursing, Heads of Academic Departments, HKMU staff, and current BScN students. The team compiled a list of accessible BScN graduates, including their workplaces, email addresses, phone numbers, and WhatsApp groups. The team also established a BScN graduate database and created a separate WhatsApp group for each cohort (2016, 2017, 2018, 2019, and 2020). Through this process, the team was able to identify the number of HKMU BScN graduates available in various workplaces, the type and level of health facility they were employed in, and their WhatsApp networks. They verified the contact details of all identified graduates within each respective cohort. This process helped establish the validity and reliability of communication between the tracer study team and the accessible graduates.

### Data collection instruments

We conducted the tracer study in compliance with the Tanzania Commission for Universities (TCU) guidelines for conducting tracer studies by university institutions in Tanzania [14], which also recommend adapting data collection tools from sources such as Schomburg, Harald (2003) [15] and International Labor Office (ILO) (2016) [16]. Accordingly, this study used an online structured questionnaire, adapted and modified from the American International Health Alliance (AIHA) nursing graduate questionnaire and the Technical Vocational Education and Training (TVET) questionnaire developed by the International Labor Office (ILO) [16]. The TCU guideline and adaptation of the questionnaire were also aligned with the Muhimbili University of Health and Allied Sciences (MUHAS) guidelines for conducting tracer studies [17]. The adaptation of the above questionnaire was based on a consensus approach among the research team members, who collaboratively reviewed and modified the tool. However, no formal computation of inter-rater or expert agreement percentage was conducted during the adaptation process. Following agreement on the final version, the questionnaire was pilot-tested among four BScN graduates who were later not involved in the online survey. The pilot study aimed to show all the procedures of the main study and determine the feasibility and testing of the data collection tool. The questionnaire was uploaded to an online server, and the four BScN graduates were invited to participate in the exercise and given access to complete the online survey tools. This process emulated all the procedures that would be used during the online survey. Feedback from graduates, platform managers, and researchers was used to improve the online system and to make the necessary revisions and amendments to the survey. Online surveys offer convenient and reliable management of the collected data. They also help avoid transcription errors and do not allow participants to modify the tools [18].

The pilot study results revealed that participants consistently interpreted and answered the questions as intended by researchers. The validity of the tool was further assessed in a workshop setting with four nursing academic staff, who jointly reviewed the tools and agreed that they demonstrated acceptable validity. The questionnaire was developed and administered in English. A data-capturing interface was created using Google Forms, an acceptable platform that links responses directly to Excel sheets. The questionnaire was uploaded to an online survey platform that incorporated the consent process, built-in error checks, a data archiving system, and backup features. The platform granted the project leader restricted access to the data, allowing for regular validation of data quality and quantity.

The online graduate questionnaires consisted of multiple-choice questions, dichotomous, Likert scale, and open-ended questions, with built-in checks and brief immediate feedback. Multiple-choice questions were primarily used to assess demographic characteristics, including employment status, current rank of employment qualification, gross monthly income, and method of job application. Dichotomous questions (e.g., Yes/No) were used to determine specific experiences or conditions, such as gender, and whether graduates underwent an internship. Open-ended questions were used to assess the job title. Likert-type scale items measured professional competencies for graduates, usefulness of teaching and learning methods, usefulness of assessment methods, study conditions of BScN graduates, and graduates’ agreement with the usefulness of the BScN program.

### Data collection procedures

The process included two stages; the first stage was to create awareness and familiarity with the online survey and its management through the WhatsApp cohort groups. The second stage was the actual online survey, which started by motioning graduates to log in using their identification numbers, leading each to a page that would provide explanations about the purpose of the study, procedures, individual and national benefits, any possible risks, confidentiality, and contact details. If a participant had a reservation, she/he would use the provided telephone numbers and email address on the information page to contact the study principal investigator (PI) for instant clarification.

Graduates who fully understood the study details were asked to indicate their preference or lack of preference to participate by clicking the appropriate option. Those who preferred to participate were granted access to fill out the online survey questionnaire. The survey links were shared in established WhatsApp graduate cohort groups. The online survey questionnaire took about 20–30 minutes to complete, and the link was accessible for over eight weeks.

To achieve a high response rate, a standard reminder text message was sent to the WhatsApp graduate cohort groups every week to remind participants to respond to the survey questionnaire. The reminder message was accompanied by a link to the online survey questionnaire, starting with the consent-giving process. The reminder message was a means to follow up with those who had not completed the surveys and to also check for any difficulties experienced in accessing or filling them out.

The reminder text message read as follows: *Dear HKMU graduate, we recently sent you an invite to our online survey [****survey link****] to let us know your experience during and after graduation and recommendations on areas that require improvement in our BScN curriculum. Your feedback on our program matters to us, and your response will help improve the quality of our program training. In case of any questions, contact the school lead [****School official email****;* ***mobile number****] in your WhatsApp cohort group. Thank you.’*

Similarly, participants were given the opportunity for multiple access to the survey questionnaire before submission. Once the participant submitted the questionnaire, he or she was restricted from any further access. The opportunity to access the online questionnaire was open for two months, after which it was closed.

The survey had inbuilt quality checks, which were invoked simultaneously with the questionnaire completion process. In case a quality issue was detected, the system would ask for rectification and eventually obtain the data, which was transferred to the HKMU server as backup.

The survey questionnaires captured graduates’ demographic information, including educational background before the study, employment status, income, methods of job search, training qualification, feedback about the BScN training program, the relationship between training and professional competencies, strengths and weaknesses of the program, and suggestions for improvement. The team ensured that the survey format was protected against data loss and that it simplified data transfer into an Excel sheet, which could later be easily imported into the analysis software.

### Data analysis

Analysis began with data cleaning, which involved removing blank responses, as well as identifying and eliminating duplicates and errors. A total of 61 graduates responded to the questionnaire. Data were analyzed using STATA version 12 [19]. The analysis included running frequency distributions for each variable, conducting quality and normality checks, and making necessary quality adjustments. Descriptive statistics were used to summarize the data, including means, standard deviations, frequencies, and percentages, as appropriate. Descriptive analysis was also conducted to assess the frequency distribution of outcomes for each competence area, including relationships with patients, clients, and communities; relationships with colleagues; teaching and learning skills; maintaining good practice; working within the system and context of health care; professional knowledge; professionalism; practical/clinical skills; and work requirements. This was done to assess graduates’ levels of acquired competencies necessary to successfully perform functions imparted by the BScN program.

## Qualitative component

The qualitative component, based on the phenomenology approach, aimed to explore graduates’ experiences during and after the BScN training program, including their perceptions of acquired nursing competencies, job performance, work-related tasks, and professional development in nursing or midwifery specialization. Additionally, we explored stakeholders’ perceptions regarding the BScN training, graduates’ professional competencies, and further professional development. Focus group discussions, key informant interviews, and in-depth interviews were conducted concurrently with the graduate online survey.

The interview guides obtained qualitative data focusing on competitiveness in the labor market, progression in the profession, and related contexts. They also ascertained from stakeholders and graduates the extent to which the BScN curriculum equips graduates with essential competencies, namely, written communication, clinical reasoning, oral communication, information literacy, and soft skills, including critical thinking and problem-solving skills. It explored curriculum strengths, deficiencies, as well as gaps that need to be addressed to enhance competitiveness and access to the labor market.

### Study settings

These were the locations of the employers, graduates, educators, and policymakers, which included private, public, and faith-based hospitals and universities, colleges, and the Ministry of Health in Tanzania.

### Study population

The target population was all HKMU BScN students who graduated from 2016 to 2020, their employers (hospital directors of nursing services, block managers), heads of departments, educators from higher learning institutions, and policymakers. Study participants were included if they met the following criteria: 1) Graduates who completed a BScN at HKMU within the past five years (2016–2020); 2) Those graduates must have provided informed consent to participate in the study; 3) Employers of BScN graduates, educators from higher learning institutions offering BScN training, and policymakers from TNMC and the MoH. Participants were excluded if they were mentally incompetent, severely ill, or could not be reached by any means.

### Sampling and recruitment of participants

The required individuals for the FGDs, IDIs, and KIIs were identified from health facilities, Universities, and policymaking bodies (TNMC and MoH). These participants were purposively selected to get the desired mix of individuals based on the following: working experience with or supervising BScN graduates; graduates’ duration of employment and their working experience in different positions or organizations; and experience with regulating the BScN program.

Qualitative sample size determination was guided by the principle of data saturation, that is, sampling continued until the collected data were enough to answer the research questions and no new insights were obtained from ongoing data collection [20]. The study involved seven participants who participated in key informant interviews (KIIs) that consisted of two educators and five policymakers. Additionally, three focus group discussions (FGDs) with 23 study participants were conducted. These consisted of one FGD with graduates, one with employers, and two with educators from the selected Universities. Among the seven graduates who were invited to participate in the FGD, only three showed up for the interview. Efforts were made to reach the rest, but they were unsuccessful. We also conducted seven In-depth interviews (IDIs) with BScN graduates (Table 2). Most of these graduates also participated in the online survey.

**Table 2:** Summary of interviews.

Type of interview	Type of respondents	Number of respondents	Number of interviews
FGD	Educators	12	2
FGD	Graduates	3	1
FGD	Employers	8	1
IDI	Graduates	7	7
KII	Educators	2	2
KII	Policymakers	5	5
**Total**		**37**	**18**

### Data collection instruments

Interview guides were designed and used to collect qualitative data from the FGDs, IDI, and KII for specific participants. For instance, the interview guides for employers asked about their views regarding the BScN program and its benefits; how BScN courses can strengthen graduates’ employment opportunities; how the employers’ recruitment criteria ensure the hiring of qualified BScN graduates; types of competencies that need to be emphasized in the BScN program; and nursing skills lacking in the existing BScN graduates working in their facilities.

Employers were asked about their views regarding the quality of the BScN training; their thoughts about further professional development for the BScN graduate; views about the relationship between the BScN program and the graduates’ performance/competencies at work; competencies that the graduate lacks, and skills lacking in the supervisor’s ward/department. The graduates were asked about their thoughts regarding the BScN program and its relationship with their current practice; challenges encountered during job searching; experiences with the BScN training; competencies from the BScN training that are applicable/redundant in the current job; and their suggestions about MSc Nursing/midwifery programs that need to be established at HKMU.

The interview guide for policymakers enquired about their suggestions to HKMU management regarding the training of BScN students; their recommendations for the competencies needed in the BScN program to help address the current health issues nationally and globally; and their views about country issues that need to be added in the BScN curriculum. For educators, the guide explored their views about the BScN program and its benefits, and how the BScN program can strengthen graduates’ ability to pursue further academic training. The interview guides for the different groups of participants were modified as data collection progressed to fit the needs of the study.

### Data collection procedures

This involved conducting KIIs, IDIs, and FGDs to gather information from graduates and other stakeholders. The data collected included biodata, required graduate competencies (both professional and soft skills), employment sectors, and the perceived usefulness of the BScN program to graduates. A team of four specialist nurses and nurse educators (two males and two females) with MSc degrees conducted the FGDs, KIIs, and IDIs. These researchers included MB, MN, ES, and MN, and they had undergone various specialty training, including MSc pediatrics nursing, bioethics, community health nursing, midwifery, and women’s health. Data collection took place at the participants’ workplaces and HKMU. Before data collection, the research team built rapport with participants and obtained their consent to participate in the study and to allow audio recording. There were no reported biases or assumptions that could influence the data collection process.

#### Key informant interviews.

KIIs aimed to obtain in-depth information about BScN graduates from individuals who have worked closely with them, such as employers, including immediate supervisors (e.g., ward in-charges, block managers). Furthermore, the KIIs targeted policymakers from TNMC and MoH who regulate nursing education and practice in Tanzania to obtain their perspectives. The KII provided general information about the BScN program and included suggestions for improving the curriculum to address current and future national and global needs. Identified participants were contacted, and appointments were scheduled using the phone numbers provided. To ensure convenience, participants were allowed to choose the dates and times for their interviews. KIIs were conducted either face-to-face or via phone. For phone interviews, the research team arranged a quiet and private room to ensure clear communication and confidentiality. Face-to-face and phone interviews were conducted by four team members in neutral locations to avoid an unsuitable environment and avoid unsuitable environments and minimize potential bias.

The tracer study team used the developed interview guides to conduct two KIIs with educators, five employers, and five policymakers from TNMC and MoH. All interviews were conducted in the Kiswahili language and were audio recorded with participants’ consent. Each KII lasted approximately 45–60 minutes, with a maximum of two interviews conducted per day by each interviewer. It took about four days to complete all the KIIs. The audio recordings were reviewed multiple times to allow for reflection and to develop familiarity with the data collected.

#### Focus group discussions.

The target participants for the FGDs included graduates, educators, and employers. The research team conducted four FGDs: two with educators, one with graduates, and one with employers (director of nursing services and block managers). Identified participants were contacted, and appointments were scheduled based on their preferred day and time. Participants were invited to the University or their workplace for the FGDs. Two teams conducted the FGDs, each team consisting of a moderator (to facilitate the discussion) and a recorder (to take field notes and manage audio recording). The FGDs were conducted face-to-face in the Kiswahili language and were audio recorded with participants’ consent. Discussions were held in neutral venues to avoid environmental bias and ensure comfort. Before each discussion, participants were assigned a unique individual number. The moderator posed questions, and participants responded in turn, ensuring that everyone contributed. The discussions continued until all questions in the guide were addressed. FGDs conducted with employers and educators each comprised of 8–12 participants, whereas the FGD with graduates included only 3 participants due to low turnout. The discussions lasted between 60–90 minutes. Each team conducted a maximum of one FGD per day (with up to two FGDs held across both teams) to allow time for reflection and preparation, thereby enhancing the quality of subsequent discussions. All FGDs were completed within two days. At the end of each session, participants received a reimbursement of 10,000/= as compensation for their time and transport.

#### In-depth interviews.

IDIs were aimed at obtaining detailed information from BScN graduates regarding their experiences during training and after graduation. Potential participants were contacted via phone, and appointments were scheduled based on their availability. The same four team members conducted the IDIs, which were held either face-to-face or by phone. All interviews were conducted in Kiswahili and audio recorded with participants’ consent.

For the phone interviews, the team ensured that a quiet and private room was prepared to facilitate clear communication. Both face-to-face and phone interviews were conducted in neutral locations to minimize environmental distractions and potential bias. The research team used interview guides to conduct seven IDIs with BScN graduates. Each interview lasted approximately 45–60 minutes, and each interviewer conducted a maximum of two interviews per day.

### Data analysis

Audio-recorded interviews conducted in Kiswahili were transcribed and saved in Microsoft Word files and subsequently translated into English. To ensure accuracy, the transcriptions were verified by listening to the original recordings and correcting errors. The interview transcripts were reviewed in their entirety to gain an overall understanding of the participants’ responses to various issues.

Data were analyzed using an inductive qualitative approach following thematic analysis as outlined by Braun and Clarke [21]. The process was iterative, with data collection and analysis occurring simultaneously. Each dataset was initially analyzed before proceeding to the next round of data collection, allowing insights from earlier datasets to inform subsequent ones. Four members of the research team developed a preliminary common codebook, which was then reviewed and refined in consultation with the rest of the team. The analysis began with a thorough reading of the transcripts to familiarize researchers with the content and to expand or refine the initial codes as needed. Four researchers (MB, MN, AS, and CM) were involved in data coding and analysis. Coding was subsequently conducted for each dataset.

Emerging codes were grouped into subthemes and overarching themes. The themes were derived from the data, and they aligned with the study’s specific objectives. To aid in the organization and comparison of data across different participant categories, we used an Excel workbook. The workbook facilitated the summarization and structuring of results. Themes were continually identified, reviewed, and analyzed to explore patterns, similarities, and differences. Regular team discussions enriched the analysis by incorporating diverse perspectives and interpretations. Final themes were clearly defined, named, and synthesized to capture key insights on graduates’ experiences of the BScN program, stakeholders’ perceptions, and recommendations for enhancing the BScN program and informing the design of future career development initiatives. This iterative and collaborative approach ensured a robust analysis that accurately reflected the range of perspectives and experiences captured throughout the study.

### Strategies to establish trustworthiness

Trustworthiness in this study was established through several strategies addressing credibility, dependability, confirmability, and transferability [22]. Credibility was enhanced through data triangulation across participant groups (graduates, educators, employers, and policymakers) and methods (quantitative surveys, qualitative interviews, and focus group discussions), as well as member checking with selected participants to clarify issues that were not clear. In addition, we applied the principle of data saturation to determine when sufficient information had been gathered from participants. For instance, the team conducted the KIIs, FGDs, and IDIs until saturation was reached. Throughout the study, interview guides were reviewed to ensure clarity while maintaining the objectives of the study.

Dependability was ensured through clear documentation of all research procedures and tools, the use of a team approach for coding and theme development, and maintaining an audit trail of analytic and methodological decisions. The audio-recorded files were listened to several times to allow time for reflection and gain familiarity with the collected data. Proper selection of suitable codes was ensured by the team during analysis to guide the development of appropriate themes.

Confirmability was supported through independent coding by multiple team members with consensus discussions and preservation of raw data for potential external audit. Transferability was strengthened by providing a thick description of the study setting, participant characteristics, and context, purposeful sampling to capture diverse perspectives, and the inclusion of detailed participant quotes in the results section.

### Integration of quantitative and qualitative approaches

In this parallel convergent mixed methods study, quantitative and qualitative data were collected and analyzed separately, then integrated primarily during the interpretation phase to strengthen the validity and depth of the findings [13]. Quantitative data from graduates’ online surveys provided information about their sociodemographic characteristics, application of competencies in professional practice, and perceptions of the BScN program. Qualitative data from focus group discussions, in-depth interviews, and key informant interviews offered rich contextual insights into stakeholders’ experiences, perceptions, and recommendations. Integration was achieved through comparing and triangulating findings from quantitative and qualitative components, identifying areas of convergence, divergence, and complementarity. This combined interpretation allowed for a more comprehensive understanding of the BScN program’s outcomes and areas for improvement.

### Ethical considerations

The tracer study team obtained ethical approval from the HKMU Institutional Research Ethics Committee (IREC) (certificate no HKMU/IREC/27.10/167). For the online surveys, the first page contained the consent information, and graduates were informed that completing and submitting the questionnaire indicated that they had read and understood the information provided and consented to participate in the study. Written informed consent was sought from participants involved in face-to-face interviews (all FGDs, KIIs, and some IDIs). We obtained oral informed consent from participants involved in phone interviews, and their consent was recorded along with the interviews.

Before data collection, participants were informed about the data collection procedures, purpose, and benefits of the tracer study; voluntary participation; and their freedom to withdraw at any time without any penalty. Graduates who participated in the online survey were also informed of their right to withdraw and their option to opt out of sharing any data provided in the survey. To uphold ethical research standards, the team ensured that the survey was designed to allow participants to skip or erase responses and navigate backward if needed. All participants were assured that their privacy would always be protected and that the information collected would remain anonymous and strictly confidential. For participants who were involved in KIIs, IDIs, and FGDs, permission to conduct the study was obtained from their local authorities and employers.

## Results

### Characteristics of the study population

The quantitative component involved 61 HKMU BScN graduates whose enrolment was from 2012 to 2016 and who graduated from 2016 to 2020. Their mean age was 28.6 years, SD (3.2). Most (60%) were female, and nearly all (98.4%) completed an internship, with 44 individuals (72.1%) employed as Nursing Officers. More than half 33, 54.1%) received a monthly income of less than a million shillings. The average duration for employment was 7.5 months (SD 9.1), with a range from 0 to 36 months, and the most common job search method was self-application (39.3%) (Table 3).

**Table 3:** Sociodemographic characteristics of BScN graduates (N = 61).

Characteristics	Number	Percent	Mean	SD
**Age**	61	100	28.6	3.2
**Sex**				
Female	37	60.66		
Male	24	39.34		
Underwent internship	60	98.4		
Working as a nurse	48	78.7		
**Current ranks of employment**				
NO	44	72.1		
RN	8	13.1		
Tutor	6	9.8		
Tutorial Assistant	3	4.9		
**Gross monthly income**				
Less than 1 million	33	54.1		
1 million-2 million	21	34.4		
More than 3.1million	4	6.6		
2.1-3 million	3	4.9		
**Year of enrollment**				
2012	2	3.3		
2013	9	14.8		
2014	14	22.9		
2015	7	11.5		
2016	29	47.54		
**Year of graduation**				
2016	3	4.92		
2017	8	13.11		
2018	14	22.95		
2019	6	9.84		
2020	30	49.18		
**Methods of job application**				
Self-application	24	39.3		
Job adverts websites	23	37.7		
Informed by a friend	10	16.4		
Newspaper job adverts	2	3.3		
None	2	3.3		

The qualitative component consisted of 37 participants (23 participated in FGDs, 7 in KIIs, and 7 IDIs), including 22 females and 15 males, with their ages ranging from 24 to 56 years. Most participants held a BScN and were fully employed in public academic and health institutions (Table 4).

**Table 4:** Sociodemographic characteristics of participants involved in qualitative interviews (N = 37).

Type of interview	Participant category	Participants	Age	Sex	Professional qualification	Facility type	Sector
FGD	Educators	P1	54	M	PhD	Education	Private
		P2	44	F	MSc	Education	Private
		P3	52	F	MSc	Education	Private
		P4	42	F	MSc	Education	Private
		P5	38	F	MSc	Education	Private
		P6	35	M	MSc	Education	Private
		P7	48	M	PhD	Education	Public
		P8	44	M	MSc	Education	Public
		P9	38	F	MSc	Education	Public
		P10	40	F	PhD	Education	Public
		P11	49	M	PhD	Education	Public
		P12	38	M	MSc	Education	Public
	Employers	P1	35	M	BScN	Health	Public
		P2	39	F	BScN	Health	Public
		P3	46	F	BScN	Health	Public
		P4	42	F	BScN	Health	Public
		P5	44	M	MSc	Health	Public
		P6	48	F	BScN	Health	Public
		P7	40	M	BScN	Health	Public
		P8	49	F	BScN	Health	Public
	Graduates	P1	24	F	BScN	Health	Private
		P2	23	F	BScN	Health	Private
		P3	25	M	BScN	Health	NGO
KII	Educators	P1	45	M	PhD	Education	Public
		P2	46	M	PhD	Education	Public
	Policymakers	P1	47	F	MSc	MoH	Public
		P2	42	F	MSc	MoH	Public
		P3	44	M	MSc	MoH	Public
		P4	55	F	MSc	MoH	Public
		P5	56	F	MSc	MoH	Public
IDI	Graduates	P1	30	F	MSc	MoH	Public
		P2	30	F	BScN	Health	Public
		P3	29	F	BScN	Health	Public
		P4	24	F	BScN	Health	NGO
		P5	25	M	BScN	Education	Private
		P6	25	M	BScN	Health	Public
		P7	28	F	BScN	Health	Public

MoH, Ministry of Health; NGO, Non-Governmental Organization

### Emerged themes from qualitative data

Analysis of qualitative data revealed several subthemes and seven main thematic areas, including stakeholders’ perception of the BScN program; experience working with BScN graduates; perceived benefits and impact of the BScN program; views regarding the quality of BScN training; relationship between acquired competencies and professional performance; challenges experienced by graduates; and recommendations to improve the BScN program (Table 5).

**Table 5:** Summary of emerged themes and sub-themes.

SN	Main themes	Sub-themes
1	Stakeholders’ perception of the BScN program	
2	Experience working with BScN graduates	• Good leadership skills • Disciplined, attentive, punctual, and committed • Proactive and eager to learn.
3	Perceived benefits and impact of the BScN program	• Improved knowledge, skills, and passion • Increased efficiency in healthcare delivery • Fostered life and networking skills • Provision of wider career choices and professional advancement
4	Views regarding the quality of BScN training	
5	Relationship between acquired competencies and professional performance	
6	Challenges during the BScN program and in professional practice	• Challenges related to curriculum content and learning experiences • Challenges related to professional practice
7	Recommendations to improve the BScN program	• Introducing a competence-based curriculum • Ensuring the availability of clinical instructors • Providing sufficient exposure to clinical practice

### Contribution of the BScN program to professional skills

Most 44 (72.1%) participants concurred that the skills in “Formulating the nursing diagnoses and prioritizing patients’ needs” enabled them to conduct their professional nursing practice effectively. Additionally, 42 (68.9%) agreed that the ability to “make appropriate plans for patients’ nursing care”, “implement the plans effectively”, and “manage patients’ pain effectively” enabled them to practice effectively in their current nursing profession (Table 6). This information is supported by qualitative findings where some educators perceived that the program is important, and it equips graduates with the necessary competencies to enable them to apply professional practical skills more effectively. A participant reported,

**Table 6:** Professional competencies for nurse graduates: Application of professional practical skills (N = 61).

Practical/clinical skills	Very unlikely	Likely	Don’t know	Likely	Very likely
Gather complete and focused patient information, in an organized manner, appropriate to the clinical situation and the patient’s or relative’s ability to understand	4(6.6)	1(1.6)	1(1.6)	22(36.1)	33(54.1)
Conduct complete and relevant nursing assessments in a systematic manner	3(4.9)	1(1.6)	1(1.6)	19(31.1)	37(60.6)
Document the nursing findings in an organized and comprehensive manner	4(6.6)	0(0)	0 (0)	18(29.5)	39(63.9)
Formulate the nursing diagnosis, and prioritize patients’ needs	4(6.6)	0(0)	0(0)	13(21.3)	44(72.1)
Make appropriate plans for the patient’s nursing care	3(4.9)	1(1.6)	1(1.6)	14(22.9)	42(68.9)
Implement the plans effectively	4(6.6)	2(3.3)	0(0)	13(21.3)	42(68.9)
Evaluate the care provided	4(6.6)	1(1.6)	1(1.6)	15(24.6)	40(65.6)
Manage patients’ pain effectively	4(6.6)	2(3.3)	0(0)	13(21.3)	42(68.9)

*“The BScN program has many benefits for graduates. We train them to be leaders in various health departments, units, and committees. These are persons who can stand on behalf of patients and suggest something for various stakeholders”* **(KII, Educator 2).**

Furthermore, employers narrated that graduates from HKMU meet the standards required for nurses, especially in clinical practice. A participant said,

*“I can say that we are proud of HKMU graduates because the majority meet the standard, especially in clinical practice. A good example is that most of the staff are granted permission for further studies at HKMU. When they graduate and come back, they are helpful and increase productivity at work”* **(FGD, Employers P5).**

### The usefulness of teaching and learning methods in the BScN program

The majority, 52 (85.2), agreed that ‘demonstration’ was a very useful teaching and learning method, followed closely by 49 (80.3%) who identified ‘patient case studies’ as useful. ‘Individual or group presentations and ‘fieldwork’ also received slightly lower but equal concurrence with 80.3% (Table 7). Qualitative data did not yield specific insights related to the usefulness of teaching and assessment methods; therefore, only quantitative results are reported for this aspect.

**Table 7:** The usefulness of teaching and learning methods (N = 61).

Teaching method	Not at all useful	Not useful	Fairly useful	Useful	Very useful
Lectures	0(0)	1(1.6)	10(16.4)	26(42.6)	24(39.3)
Lecture-Discussions	0(0)	2(3.2)	1(1.6)	16(26.2)	41(67.2)
Seminars/Tutorials	0(0)	4(6.6)	7(11.5)	22(36.1)	28(45.9)
Demonstration	0(0)	0(0)	1(1.6)	8(13.1)	52(85.2)
Practical skills	0(0)	1(1.6)	5(8.2)	14(23.0)	41(61.2)
Bedside teaching	0(0)	1(1.6)	2(3.2)	14(23.0(	44(72.3)
Family case studies	0(0)	1(1.6)	7(11.5)	20(32.8)	32(52.5)
Patient case studies	0(0)	0(0)	0(0)	12(19.7)	49(80.3)
Fieldwork	1(1.6)	1(1.6)	5(8.2)	9(14.8)	45(73.8)
Individual or group presentations	0(0)	0(0)	4(6.6)	12(19.7)	45(73.8)
Self-reflection	1(1.6)	2(3.2)	6(9.8)	25(41.0)	27(44.3)
Group and individual assignment	1(1.6)	2(3.2)	2(3.2)	20(32.8)	36(59.0)
Simulation	0(0)	0(0)	2(3.2)	23(37.7)	36(59.0)
Roleplay	1(1.6)	1(1.6)	3(4.9)	22(36.1)	34(55.7)
Microteaching	1(1.6)	2(3.2)	1(1.6)	16(26.2)	40(65.6)

### The usefulness of assessment methods in teaching and learning

Most participants, 47 (81.0%), agreed that clinical/practical examinations were very useful methods of assessment in teaching and learning. These were followed by end-of-semester/final examinations, with 46 (77.9%) of participants concurring on their usefulness (Table 8). Similarly, stakeholders supported the use of practical examinations as an effective method for assessing learning. They also emphasized the need for a competency-based curriculum that would be reflected not only in teaching but also in the assessment process. One participant in a focus group discussion expressed:

**Table 8:** The usefulness of assessment methods in the BScN program (N = 61).

Assessment method	Not at all useful	Not useful	Fairly useful	Useful	Very useful
Continuous Assessment	0(0.0)	1(1.6)	0(0.0)	15(24.6)	45(73.8)
End of Semester/Final Examinations	1(1.6)	1(1.6)	0(0)	11(18.0)	48(78.7)
Objective-type questions	0(0.0)	1(1.6)	5(8.2)	25(41.0)	30(49.2)
Essays	0(0.0)	0(0)	3(4.9)	26(42.6)	32(52.5)
Short Answers	0(0)	1(1.6)	3(4.9)	18 (29.5)	39(63.9)
Oral Examinations	0(0.0)	4(6.6)	8(13.1)	17(27.9)	32(52.5)
Field Work/ Projects	0(0)	2(3.3)	15(24.6)	1(1.6)	43(70.5)
Clinical/ Practical Examinations	0(0)	0(0.0)	2(3.3)	11(18.0)	48(78.7)
Research Reports	0(0)	1(1.6)	3(4.9)	18(29.5)	39(69.3)
Presentations	0(0)	0(0.0)	2(3.3)	14(23.0)	45(73.8)
Graded assignment	0(0)	0(0)	4(36.6)	16(26.2)	41(67.2)

*“Lastly, it is all about improving the type of exams. Lecturers impart knowledge, but at the end of the day, assessments should reflect practical skills. Therefore, we suggest that universities prioritize practical exams, such as Objective Structured Clinical Examinations (OSCEs), rather than relying too much on theory”* **(FDG, Employer 5)**.

### Learning experiences during the BScN program

Participants rated their experiences during the BScN program, with more than 90% reporting positively on several aspects. These included contacts with fellow students (96.4%), availability of technical equipment (91.1%)- such as lab equipment, computer labs, and skills lab)”, availability of learning materials (91.2%)-such as books, and internet access, and availability of teaching materials (92.9%)-such as projectors, and computers (Table 9). However, qualitative findings revealed that graduates expressed concerns about the shortage of equipment necessary for skill development and competence. One graduate shared concern regarding the limited availability of certain equipment in the university’s skill lab:

**Table 9:** Study conditions of BScN graduates (N = 61).

Study condition	Very bad	Bad	Don’t know	Good	Very good
Quality of classroom learning	**6(9.8)**	**13(21.3)**	**4(6.6)**	**27(44.3)**	**11(18.0)**
Student recreational facilities on campus	1(1.6)	4(6.6)	0(0.0)	29(47.5)	27(44.3)
Availability of learning materials (e.g., books, internet access)	5(8.2)	10(16.4)	5(8.2)	25(40.9)	16(26.2)
Grading system	0(0.0)	0(0.0)	3(4.9)	33(54.1)	25(41.0)
Contacts with fellow students	1(1.6)	4(6.6)	9(14.8)	33(54.1)	14(23.0)
Chances for students to influence policies	1(1.6)	3(4.9)	1(1.6)	29(47.5)	27(44.3)
Availability of technical equipment (e.g., lab equipment, computer lab, skills lab)	2(3.3)	5(8.2)	2(3.3)	31(50.8)	21(34.4)
Quality of technical equipment (e.g., mannequins, equipment for various procedures)	0(0.0)	2(3.3)	2(3.3)	25441.0)	32(52.5)
Availability of teaching materials (e.g., projectors, computers)	2(3.3)	2(3.3)	4(6.6)	29(47.5)	24(39.3)

*“Ah, there were also a few mannequins; you find yourself... umm... There were few, and there were certain mannequins, for example, mannequins for... for doing resuscitation... cardiopulmonary resuscitation (CPR), there was only one. Increase the number of accessories in the skills lab. And make the skills lab larger, as I mentioned.”* **(IDI, Graduate 4).**

Participants also commented on the quality of infrastructure, particularly lecture rooms, noting that they were insufficient to accommodate the growing number of students and academic programs. One graduate remarked:

*“Furthermore, regarding the buildings, there was a challenge with insufficient buildings. There was a lot of movement that caused a lot of interference for individuals, and it didn’t create a conducive learning environment. We would shift from one classroom to another, as there is no permanent class that is sufficient and conducive for us.* **(IDI, Graduate 1).**

### The usefulness of the BScN program

Participants generally agreed that the BScN program helped secure employment, though fewer believed it was useful for long-term professional career development. The program’s ‘fulfilling present tasks’ was rated high at 94.7%, followed by “development of personality” at 92.9%. However, the lowest rating was for “future professional development/career”, which was endorsed by only 64.9% of participants (Table 10).

**Table 10:** Graduates’ agreement with the usefulness of the BScN program.

The usefulness of BScN studies	Not at all useful	Slightly not useful	I don’t know	Useful	Very useful
For finding an adequate job after finishing your studies	1(1.6)	5(8.2)	3(4.9)	26(42.6)	26(42.6)
For fulfilling your present tasks, if applicable	0(0)	1(1.6)	2(3.3)	32(52.5)	26(42.6)
For your future professional development/career	0(0)	3(4.9)	17(27.9)	1(1.6)	40(65.6)
For the development of your personality	1(1.6)	3(4.9)	0(0.0)	21(34.4)	36(59.0)
For the economic development of your country	3(4.9)	1(1.644)	2(3.3)	25(41.0)	30(49.2)

Respondents noted that the BScN program equips graduates with advanced knowledge that they can apply in various areas. For instance, they can integrate knowledge from basic sciences (such as anatomy and physiology) into clinical practice and utilize nursing research skills in conducting research. These competencies help graduates perform their daily tasks more effectively. One graduate shared:

*“The major course content that has helped me a lot is basic science, for example right now I’m pursuing MSc in anatomy, so anatomy helps me a lot when I combine it with physiology”…. “…. professionally, I am well-equipped, so I have been able to teach, do research, consult, and help anyone who has come up with something related to my profession…….* **(IDI, Graduate 5)**

Graduates also appreciated the BScN program for equipping them with improved clinical skills that enable them to provide efficient care to clients with a variety of conditions, including surgical, medical, or otherwise encountered in daily practice. These skills have enhanced their clinical reasoning and decision-making abilities during the execution of procedures. One graduate reported:

*“For example, there are times when I’m not busy with administration issues, I stay in the minor theater, I do these minor procedures like incision and drainage, and if there are people who come with a cut wound, I know how to do stitches. If there are people who come with a fracture, I apply POP ….”* **(IDI, Graduate 3)**.

The BScN training program paved the way for most graduates to secure their current positions, increase their visibility, and enabled them to compete effectively in the job market. For example, one of the graduates said:

*“…. I would not be able to get a teaching job at Tandabui College, as well as be appointed as an examination officer. So, the program has helped me a lot to get a job and to develop myself in teaching, which means without the program I would probably be on the streets.”* **(IDI, Graduate 4).**

Information from several graduates indicated that the BScN program played a significant role in shaping and enhancing their personality traits, including self-confidence, passion, and discipline. For instance, some graduates shared that the program helped to boost their confidence in performing daily duties and handling routine challenges. One graduate stated:

*“…. I feel that I am doing well because I have confidence, I work freely and peacefully because I know many things. But apart from that, it has also helped me deal with many challenges in the community,”* **(FGD, graduates P2).**

Participants also reported that the BScN program strengthened their passion for the nursing profession. Graduates noted that, throughout the program, faculty consistently emphasized the importance of discipline and inspired them to provide care with compassion and dedication. One graduate shared:

*“We are also taught about discipline, how to dress, to love what we study, not only as a source of income but also to do it with passion. The lecturers inspire us to love our studies and practice from the heart with passion”* **(IDI, Graduate 1).**

### Challenges during the BScN program and in professional practice

Findings from the qualitative interviews revealed several challenges experienced by graduates during and after the BScN program. These challenges were categorized into two sub-themes: challenges related to curriculum content and learning experiences, and those related to professional practice.

### Challenges related to curriculum content and learning experiences

Participants reported several challenges experienced during their training, including repetition of content across some courses, the absence of critical care and emergency nursing topics, and insufficient time allocated for clinical practice*.* The repetition of the theoretical content across different semesters was said to reduce the time available for clinical or field-based learning. In addition, the current BScN curriculum lacks essential content in emergency and critical care nursing, which limits students’ ability to acquire the necessary skills for managing acute and life-threatening conditions. Some participants recommended the inclusion of additional modules in the revised BScN curriculum, such as customer care, communication skills, and infection prevention and control (IPC). One participant explained,

*“You may find the same course content repeated in this course and that course, and time is squeezed to fit everything, there are a lot of repetitions some of which we have not experienced at all … also, the system we used did not give us a direct access to study emergency nursing, so we studied emergency conditions in other courses leading to lack of clinical skills”* **(IDI, Graduate 5).**

Additionally, an experienced educator reported that student nurses do not get sufficient time to practice the skills taught in class, which contributes to a lack of competence during their training. She stated:

*“I see that in nursing degree training, a large percentage of students do not get enough time to practice, which leads to a lack of skills and competencies. I think this is the biggest challenge in the BScN degree program”* **(KII, Educator 1).**

### Challenges related to professional practice

Participants encountered several challenges during the internship and at current employment, including staff shortage, inadequate clinical skills, lack of adherence to standard practice, and limited opportunities to apply their acquired knowledge. Some participants reported an inability to apply their knowledge and skills as per professional standards because many nurses followed routine practices rather than evidence-based guidelines. Additionally, due to staff shortages, particularly of nurses and doctors, participants were sometimes required to perform procedures beyond their scope of practice to save patients’ lives-actions they acknowledged as necessary but professionally unacceptable.

One participant said:

*“Our institution faces a challenge of staff shortage, encompassing not only nurses but also doctors. Consequently, there are tasks outlined in my job description that I am expected to fulfill. However, certain procedures, such as the application of POP and incision and drainage, fall outside my scope of practice, and I am not licensed to perform them. Nonetheless, I am required to carry out these procedures in the absence of doctors.”* **(IDI, Graduate 3).**

The findings also revealed that some participants felt incompetent performing clinical procedures during their internship and after gaining employment. However, they reported having basic skills and adequate knowledge. One of the participants explained:

*“During the internship, I felt that I could not practice at all. Though I could perform some procedures to some extent, I did not feel competent enough.”* **(IDI, Graduate 1).**

Participants reported difficulty applying their knowledge and skills according to the required nursing standards of practice, as senior nurses often adhere to routine practices that offer limited opportunities for growth. Graduates who attempted to introduce changes in nursing practice were frequently met with resistance, perceived negatively, and at times, isolated. One participant narrated:

*“The main challenge is that people adhere to professionalism only to a small extent. When you try to do things correctly as they should be done, you find yourself different from your colleagues, and you can be singled out for wanting to do too much.”* **(IDI, Graduate 3).**

Participants expressed a strong desire to innovate and improve nursing education, aiming to enhance the professional image of nursing. However, they encountered challenges in decision-making, which they attributed to the hierarchical nature of the health system. Despite actively participating in doctors’ rounds, their input was often limited. One participant expressed:

*“Although the environment was not conducive, I wanted to implement innovative practices to distinguish myself from those with lower qualifications. Nonetheless, I found myself particularly focused during doctors’ rounds”* **(IDI, Graduate 2).**

### Recommendations to improve the BScN program

The following recommendations are based on the analysis of qualitative data that involved the perspectives of stakeholders, including graduates, employers, educators, and policymakers.

### Establishment of a competence-based curriculum

The participants recommended a shift from a content-based to a competency-based curriculum, emphasizing critical aspects such as the nursing process, research, leadership, computer literacy, critical thinking, decision-making, and problem-solving skills. They believed that this approach would better equip students with the knowledge, skills, and attitudes required for effective performance in their future nursing roles. The study highlights the need to enhance graduates’ ability to competently manage patients, thereby increasing uniqueness and relevance in the current job market.

Stakeholders raised concerns about the limited application of the nursing process among graduates. They recommended that greater emphasis be placed on its integration into the curriculum. This includes teaching students to systematically assess, diagnose, plan, implement, and evaluate patient care, to ensure a holistic, patient-centered nursing practice. One participant shared:

*“The nursing process has become a significant challenge; we rely on those with BScN to help us in this area, but they also need adequate training……. The nursing process is the core area that drives nursing practice; if I can’t practice that, it becomes a challenge to apply it”*** (KII, Policymaker 2)**.

Participants also emphasized that a competency-based curriculum should prioritize the development of strong decision-making and critical thinking skills among nursing students. They suggested that this would require creating opportunities for students to analyze complex healthcare scenarios, evaluate various options, and make informed decisions grounded in critical thinking and evidence-based practice. Additionally, they highlighted the need to incorporate activities and assignments that challenge students to analyze and evaluate information, think critically, and apply problem-solving strategies in diverse clinical situations. One of the key informants stated:

*“…. the challenge I will talk about is my experience when receiving those who come with a bachelor’s degree; well, to be honest, even in terms of decision-making, it is still a challenge because as a nurse, you are expected to have a sharp mind, and keen eyes to observe, make decisions, and implement them. I have seen it as a significant challenge”* **(KII, Policymaker 1).**

Another participant added,

*“Therefore, we need to develop a curriculum that creates self-dependent individuals. In terms of critical thinking, applied sciences, and knowledge and technology application, it should enable individuals to provide services in all areas”* **(KII, Policymaker 5).**

Participants also emphasized the importance of allocating sufficient time within the BSc Nursing curriculum to equip students with the knowledge and skills required to conduct research effectively. In addition, they highlighted the need to strengthen computer literacy, enabling graduates to proficiently use various computer applications and electronic health record systems. Mastery of both research and computer skills would empower graduates to critically analyze evidence and contribute meaningfully to the advancement of nursing practice and improved healthcare outcomes. One of the graduates remarked:

*“…Research is a valuable course that can have long-lasting impacts, not just for today or tomorrow. So, we should make it a priority, not just as a subject that we rush through and finish quickly.* **(IDI, Graduate 2).**

The same participant added:

*“Strengthening computer skills can enhance students’ efficiency in documentation, data management, and utilizing technology for evidence-based practice”.* **(IDI, Graduate 2).**

### Providing sufficient exposure to clinical practice

Study participants highlighted the importance of providing students with ample opportunities for clinical practice. This includes clinical placements in diverse healthcare settings, which enable students to apply theoretical knowledge, develop clinical skills, and build confidence in delivering patient care. In addition to the graduates, both policymakers and employers expressed concerns about the limited time allocated for practical exposure in many nursing curricula, as illustrated in the following statement:

*“When I look at our nursing history, in the past, we were taught as if we were going to become leaders. Therefore, in the new curriculum, I would like to see an adequate amount of time allocated for practical training.”* **(KII, Policymaker 3).**

Another participant added:

*“So, having more hours in the clinical area and fewer hours in the classroom, based on how the curriculum will be structured, what they will gain in the classroom, and given the majority of their time is spent on-site, they will be able to link it together, and in the end, it will result in a good product.”* **(FGD, Employers P6).**

### Ensuring the availability of clinical instructors

Furthermore, the study participants emphasized the importance of ensuring the availability of clinical instructors who can provide guidance, supervision, and support during students’ clinical placements. Having an adequate number of skilled instructors would enhance the students’ learning experience and facilitate the application of theoretical knowledge in real-world healthcare settings. As expressed by one HKMU graduate:

*“I see the need to increase the number of instructors, especially in clinical settings. I believe that clinical practice should be given equal importance to theory. Sufficient clinical instructors should be available to assist students in learning practical skills”* (**IDI, Graduate 1**).

## Discussion

This study found that the BScN program effectively prepares graduates for professional nursing roles, with practical and demonstration-based methods rated most useful. Stakeholders confirmed the program’s positive impact while identifying areas for improvement, particularly in aligning acquired competencies with labour market demands. These findings provide insights for enhancing nursing education, guiding curriculum development, and informing workforce and policy planning.

### Learning experience and the contribution of the BScN program to graduates’ professional skills

Despite the reported shortage of lecture rooms and certain equipment in the skills lab, graduates highly rated the availability of other essential equipment, learning resources, and teaching materials in the training institution. Consistent with our findings, previous studies have shown that well-equipped learning environments in nursing programs enhance student engagement and academic performance. Access to adequate resources, such as clinical laboratories, libraries, and digital learning tools, plays a crucial role in supporting effective learning among nursing students [23].

Graduates affirmed that the BScN program significantly improved their competencies, clinical skills, and theoretical knowledge. Likewise, employers consistently recognized these competencies as key strengths among nursing graduates, indicating that the program effectively prepares students for the practical demands of the healthcare sector and positively impacts job performance [6].

As a result of the BScN training, graduates reported a high success rate in securing suitable employment, pursuing academic opportunities, and achieving professional recognition [9], as well as advancing in their careers [24]. Graduates viewed competency mastery as essential for professional growth-nurses who demonstrate strong competencies are more likely to be promoted. These competencies are often reflected in the quality of care provided, which contributes to greater patient satisfaction [25].

This suggests that the BScN curriculum and clinical exposure effectively prepare competent nurses to meet labor market demands and support long-term career development [26]. Similarly, previous studies highlight the critical role of effective clinical practice in equipping nursing students with the skills required for professional performance. Practical clinical exposure enables students to apply theoretical knowledge in real-life settings, thereby fostering essential skill development [27,28].

Furthermore, adequate supervision, consistent support during clinical placements, and strong collaboration between academic institutions and clinical facilities contribute to improved student outcomes and the successful translation of theory into practice [29].

Other studies have reported similar findings, where BScN graduates acknowledged that the program enhanced their ability to work more efficiently and effectively [2,4,9]. Nurses’ job performance is closely tied to their competencies and educational background [10]. Graduates reported improved job performance and work competence after completing the BScN program. These improvements were attributed to the ability to apply theoretical knowledge in practice, leading to increased confidence and autonomy in their professional roles [1]. The knowledge and skills acquired enabled graduates to perform daily tasks effectively and adapt to various areas of nursing practice, indicating that the training was comprehensive and well-delivered. These results align with findings from the East Africa (EA) tracer study, which reported that graduates from private universities acquired the competencies needed for employment [26]. Similarly, students’ perceptions and experiences with the curriculum and academic support showed that they found the BScN program engaging and believed it sufficiently prepared them for clinical practice [31]. Other research also confirms that university education equips graduates with relevant competencies applicable across various fields of work [30,32,33], emphasizing the importance of adaptability and the ability to apply skills in diverse contexts [34].

The results highlighted significant improvements in graduates’ attributes, including discipline at work, self-confidence, and passion for the nursing profession. For instance, graduates in other studies reported choosing to pursue a BScN degree due to a strong passion for the field [10], which supports the findings of this study. Education was shown to play a crucial role in enhancing both individual autonomy and the ability to function effectively within a team, which are essential in nursing practice. Moreover, the development of core competencies contributed to greater professional commitment and confidence, ultimately enhancing job performance [11]. Graduates also indicated that the program reinforced their discipline as professional nurses. This aligns with existing literature that underscores the importance of instilling professional values such as honesty, collaboration, and discipline within nursing education. [12]. These personality traits are consistent with findings from previous studies that stress the need for ongoing monitoring and reinforcement of discipline throughout both academic and clinical training [32,33].

### The usefulness of teaching, learning, and assessment methods in the BScN program

The teaching and learning methods used in the program support students in developing essential competencies and applying their knowledge and skills in professional practice. This finding aligns with previous studies. For instance, the introduction of new concepts, effective supervision, and the provision of autonomy were identified as key factors enhancing clinical learning among nursing students. Clinical experiences serve as valuable learning opportunities that contribute to the development of their competencies [35]. Although lecture-based teaching remains the most commonly used method in higher education institutions, the practical nature of nursing education necessitates a strong focus on skill acquisition [36]. For example, Bachelor of Science in Nursing students who were exposed to both video-assisted teaching and traditional demonstrations on obstetrical palpation showed improved skills with both methods; however, traditional demonstrations were significantly more effective [37]. Similarly, the demonstration method proved more impactful in enhancing both knowledge and practical skills in medical-surgical nursing courses than traditional classroom lectures alone [36]. Demonstrations are also integral to clinical training in nursing. While both lectures and clinical training contribute to knowledge and skill development, nurses who received clinical training on caring for patients with angina achieved higher scores and demonstrated greater confidence and long-term retention compared to those who only received lecture-based education [38].

Overall, demonstration-based methods tend to be more student-centered and engaging, making them more effective in capturing attention and enhancing learning outcomes than traditional lectures [36]. However, contrasting findings in another study indicated that some learners were not adequately equipped with the skills and knowledge required for evidence-based clinical care [39].

Consistent with our results, previous studies have also highlighted the effectiveness of assessment methods during training. Students noted that clinical examinations provided them with opportunities to demonstrate both knowledge and practical skills. Moreover, they expressed a preference for multiple-choice questions (MCQs) to assess theoretical knowledge and objective structured clinical examinations (OSCEs) to evaluate practical skills [40]. Scholars have emphasized the importance of using a well-balanced mix of assessments to be effective. There is a need for programs to develop the right combination of assessment methods and select trained assessors to ensure the reliability and validity of the assessment process [41]. Additionally, it is important for institutions to consider students’ preferences when designing assessments, as these preferences often vary depending on individual learning styles. Aligning assessment strategies with students’ preferences can enhance learning outcomes and contribute to a more effective and student-centered educational framework [42].

### Challenges related to curriculum content, learning experiences, and professional practice

Our findings revealed several challenges encountered by BScN graduates both during and after their program. Notably, participants reported inadequate time allocated for clinical exposure, which hindered the development and mastery of essential clinical competencies. Furthermore, the curriculum was reported to lack critical content areas, such as the management of emergency conditions, customer care, communication skills, and infection prevention and control (IPC). These gaps in the curriculum mirror findings from other studies, which have recommended curriculum improvements that include extending the time and credit allocation for communication skills and patient-centered care, incorporating customer care modules, and fostering a supportive clinical practice environment to enhance interpersonal relationships [43]. Similarly, a lack of IPC content within the BScN curriculum has been highlighted as a significant concern in related research [44]. These findings underscore the urgent need to review and revise the current curriculum in favor of a competency-based approach that equips learners with the skills, knowledge, and attitudes required to deliver high-quality care.

Consistent with our findings, prior research has identified several factors that negatively impact student nurses’ educational experiences, including limited engagement in clinical practice, insufficient support from faculty, and an overloaded curriculum with non-essential subjects. These factors impede students’ ability to effectively integrate into their clinical roles [45,46]. Conversely, studies have shown that academic achievement among student nurses is significantly enhanced by including an interactive curriculum, comprehensive clinical orientation and exposure, and strong support from nurse educators [47].

Challenges reported in professional practice included inadequate clinical skills, non-adherence to standard practices, staff shortages, and difficulties in applying acquired knowledge. These findings are consistent with previous studies that have identified similar barriers, including insufficient clinical competencies, lack of confidence, weak organizational support structures, poor resource allocation, and continued reliance on routine, non-evidence-based practices among nursing professionals [48,49]. Such challenges are known to substantially hinder the ability of nurse graduates to deliver effective and high-quality patient care [50].

### Recommendations to improve the BScN program

Participants emphasized the importance of allocating sufficient time for nursing students to gain hands-on clinical experience, highlighting that adequate practical exposure is essential for mastering clinical procedures and delivering safe, effective patient care. Inadequate time in clinical settings has been linked to deficiencies in clinical skills, which may contribute to compromised patient outcomes. Extending the duration of clinical placements bridges the gap between theoretical knowledge and its application in real-world settings, thereby improving the quality of care delivered by nursing graduates [51]. This underscores the need to review and revise the current curriculum to ensure that the BScN program equips graduates with the competencies required to meet the evolving healthcare needs of the population [52].

The revised BScN curriculum should prioritize the integration of critical thinking into all aspects of nursing education, as it is a foundational element for professional judgment and clinical decision-making. While existing efforts have aimed to foster critical thinking, research indicates that further advancement in teaching strategies is needed to fully embed these skills in clinical practice, education, and research [3]. Despite ongoing initiatives, significant challenges remain in developing and implementing effective pedagogical approaches that nurture critical thinking in BScN students [53]. Addressing these gaps is essential, as the formulation and delivery of vocational nursing education programs continue to represent a substantial challenge within nursing education [54].

Participants also emphasized the importance of having clinical instructors available during training, consistent with previous studies that highlight the significant impact of clinical instructor effectiveness on nursing students’ learning outcomes. Effective clinical instructor behaviors not only enhance clinical teaching but also improve the overall learning process for students [55]. Studies indicate that when instructors are not sufficiently present in clinical settings, valuable learning opportunities for nursing students are limited. This lack of supervision hinders students’ ability to fully engage in hands-on practice, which is essential for developing clinical competence and confidence [56]. Furthermore, the quality of the relationship between nursing students and their instructors is crucial in fostering satisfaction and competence within the learning environment, thereby contributing positively to student development and success [57].

### Strengths and limitations

This study has several strengths that enhance the credibility, relevance, and utility of its findings. First, it employed a Convergent Parallel Mixed-Methods Design, enabling the collection of both quantitative and qualitative data concurrently, which allowed for triangulation of findings. This methodological approach enhanced the credibility and depth of the results by comparing and corroborating data from different sources. Second, the involvement of diverse stakeholders (graduates, employers, educators, and policymakers) provided comprehensive insights into the effectiveness of the BScN program. Third, the study used structured and contextually adapted tools from internationally recognized sources (AIHA and ILO), ensuring relevance and rigor.

Despite its strengths, the study has several limitations that should be considered when interpreting the findings. The response rate was 33.3% (61 out of 182 graduates), which may limit the representativeness of the findings. Also, the use of an online questionnaire could have excluded graduates with limited internet access. A small group of graduates (three participants) for the FGD may have constrained the diversity of perspectives captured. Additionally, while a Convergent Parallel Mixed-Methods Design was employed, not all findings were fully integrated across methods due to the nature of the data collected. For instance, the recommendations for improving the BScN program were based solely on qualitative data. The quantitative component did not assess these recommendations, which may limit the extent of integration between the two data strands. Similarly, only quantitative results (such as the usefulness of teaching and learning methods) were presented, as qualitative data did not provide supporting or refuting insights relevant to those specific findings. This limited the extent of data integration in certain areas of the analysis. Furthermore, the adaptation of the questionnaire was based on a consensus approach among the research team members, and we did not compute formal measures of validity and reliability such as the Content Validity Index, percentage of agreement among experts, internal consistency (e.g., Cronbach’s alpha), or test-retest reliability which may limit the ability to quantitatively assess the psychometric properties of the instrument used in this study.

## Conclusion

The study has provided important insights into the characteristics of BScN graduates, the relevance of acquired competencies to their professional roles, and their perceptions of the overall program. Most graduates reported that the program effectively prepared them for the needs of the nursing profession, with demonstration-based teaching and practical examinations rated as particularly useful methods. Qualitative findings from graduates, employers, educators, and policymakers reinforced these results, highlighting the program’s positive impact while also identifying challenges experienced during training and offering recommendations for improvement.

Both the quantitative and qualitative findings provide a comprehensive understanding of the strengths and areas for improvement within the BScN curriculum. These results serve as a critical resource to inform ongoing curriculum review and development, ensuring that the program remains responsive to the evolving needs of the healthcare system and continues to produce competent, well-prepared nursing professionals.

## Recommendations

Based on the study findings, it is recommended that nursing education programs enhance practical and demonstration-based teaching and assessment methods, which were found to be particularly effective in preparing graduates for professional roles. It is essential to align the curriculum content and workplace competency needs, which requires continuous collaboration between educators, practitioners, and policymakers. Nursing practice environments should support the application of acquired competencies by ensuring appropriate supervision, mentorship, and professional development opportunities for new graduates. Policymakers are encouraged to institutionalize regular graduate tracer studies and curriculum reviews to maintain responsiveness to healthcare system needs. Future research should explore longitudinal outcomes of nursing graduates and the impact of curriculum revisions on professional performance and healthcare delivery.

## Supporting information

S1 File Qualitative dataset. (PDF)

S2 File Quantitative dataset. (XLS)
