# Supplementary material for: Bridging training and practice gap: A mixed methods tracer study of bachelor of science in nursing graduates (2016–2020) at Kairuki University, Dar es Salaam, Tanzania
Source: PLoS One. 2025 Oct 3;20(10):e0333702. doi: 10.1371/journal.pone.0333702 (PMC12494258; doi:10.1371/journal.pone.0333702)
Supplement: S1 File — (PDF) [file pone.0333702.s001.pdf]

FGD Educators #1

Interviewer (I): Okay, welcome again.

Participants: Thank you.

I: Okay, we'll start with participant number one. What are your thoughts regarding the bachelor's degree in nursing education?

Respondent 1 (R1): Thank you for the question. The bachelor's degree in nursing education is good because it puts those learning nursing in a good position, although nothing lacks both advantages and disadvantages. I don't know the correct Swahili to use... in those pros and cons of each one. It appears that in the bachelor's degree in nursing education, students largely don't get enough opportunities to do practical training. I think that's a bit of a challenge in the bachelor's degree training. But also on issues of... I don't know the right Swahili, attitude—that studying nursing at the bachelor's level, they think they're going to work as rulers and not to work as regular nurses. So even in the work environment, there are some challenges on that side. Thank you.

I: Thank you very much. Let's come to you, respondent number two. What are your views regarding the bachelor's degree in nursing?

R2: Umm... my opinion is that the world is changing, and because the world is changing, our young people being prepared for the bachelor's in nursing must have curricula that align with the way the world is moving. For example, ahh, if you look at the trend [I: Mmh], in the next fifty or ten or twenty years, many jobs will be taken over by ward-mounted machines, including surgery that might be done by machines [I: Mmh], and you can see machines entering the health field in many areas. But it's an undeniable truth that the nursing degree will not be replaced by machines because it still requires human caring—to care for the patient. So knowledge acquired must be of a very high level, meaning they must be critical thinkers, be able to reason, analyze thoroughly, and have the attitude to care for a patient as a machine might—but better, as a human cares for another human. So the nursing degree must have the capacity to enable adaptive learning, so that students can learn, unlearn, and then learn again. What you know today may not be true tomorrow. So it should enable that adaptive revolution—to help the student understand that learning is a process: to learn, unlearn, and learn again—because knowledge is expanding and very complex in the world we're heading into.

Interviewer (I): Okay, thank you. Now how can we overcome — does it mean the current curriculum does not prepare our students or graduates to help others learn from them?

Respondent 2 (R2): Mmmh, it's not that it doesn't prepare them [I: mmh], it does prepare them. But the process of preparing them must be one where you learn and then learn again. That is, you must be a lifelong student because knowledge changes every day. What you know today may no longer be true tomorrow. You might have a very good skill today, very complex, but tomorrow that skill may no longer be relevant. So you must have the ability—like using a phone, for example. When a new phone model comes out, you don't go to school to learn it; you start using

it right away, leaving behind what you previously learned. So you have to be able to teach yourself—you become a "life-long learner." Now, if we prepare them this way rather than just giving them too much information, which later becomes a burden instead of being beneficial… What they have is good, but it needs to be aligned with the changing world.

Interviewer (I): Thank you. Number three, what are your thoughts on the bachelor's degree in nursing?

Respondent 3 (R3): My thoughts about the nursing degree are that we truly need graduates who finish the degree—especially if we want services to improve further. We really rely on those who finish this program. And if we look back—comparing previous years to now—or if we compare different levels, like those who hold diplomas versus the education offered in the degree program, it's very different. Because at the diploma level, or in the sciences, the sciences are not taught in much depth. And nursing really needs science—we say nursing is both a science and an art. So at the degree level, science is taught more, and when science is well taught, it greatly helps in service delivery to patients. Another thing I've noticed, as my colleague mentioned, is that we are looking ahead and the world is changing very fast. Technology is essential, and it needs to be significantly emphasized in the nursing degree. Right now, everything is becoming paperless—everything is digital, even patient documentation will be computer-based. I once thought—just while sitting around—I said to myself, hmm! In this nursing degree, we should add a component like BSc and IT. That is, IT should be strongly emphasized in the BSc program, because IT is rising rapidly in the professional world. So it would really help the graduate completing a BSc in nursing when they go to work, and even in their ability to deliver services—it would help them a lot. That's all from me [laughs].

Interviewer (I): Okay, thank you very much. Let's come to you, number four.

Respondent 4 (R4): Thank you. The nursing degree program is good because I believe that at the degree level, we expect a person to have advanced knowledge, and their education will have elevated. And because respondents two and three have already said that science is evolving—now if science is changing, and we don't have people capable of learning and understanding that science, how will they perform? So we must have this degree level that includes people who have reached a certain level of education. Here, they enter with form six (advanced level secondary education), which is already a higher level compared to secondary school. Some also apply for diploma programs. But I believe the person taking this nursing degree, by the time they finish, cannot be the same as someone with a diploma or certificate in nursing. Their education will be higher to the extent that when changes occur, it's easier for them to adapt to environmental changes. Because they'll know what's important, what's current, how the world is moving, and how to adapt with advancing technology… and science and technology keep progressing. When new skills arise, it's easier for them to catch up. And Tanzania is not an island—we are part of the world. So, someone with a nursing degree here can work elsewhere, where similar standards and education systems exist. That person could work in various national organizations—not just remain a nurse who distributes medicine or performs simple tasks and fails to handle more complex duties. So, I think it's a good level to have.

Interviewer (I): Okay, before I leave you [R4: Yes], let me ask you another question [R4: Mmh]. Do you see any benefits of this nursing program for nurses?

Respondent 4 (R4): There are benefits [I: Mmh], because… [I: I'd like your opinion on those benefits] yes, there are benefits. I try to look at it this way—if I place a degree nurse and a diploma nurse side-by-side, the degree nurse often, even when we're teaching them, we must teach them to reason. That is, they don't just go through the motions—they must reason through what they are doing. Why is this happening? What results am I aiming for? Compared to the diploma or certificate nurse, who mainly knows **what to do**, not necessarily **why** to do it. Reasoning isn't emphasized much at that level. So, we need people who can reason—not just follow procedures. In providing care [I: Mmh], they must reason out what they're doing and why. That's a big benefit.

Interviewer (I): Thank you. Number three—what are your thoughts regarding the benefits of the nursing degree program?

Respondent 3 (R3): Ah… mmmh, the nursing degree has benefits. Because we can even say—we're increasingly looking to the future. We expect this nurse also to be someone who can work even without… sometimes they should be independent—even in their duties. So, even tasks like deciding: this patient has this issue, maybe I should administer this and that IV fluid—they should be able to do so without waiting for a doctor to give instructions. They should have the knowledge to recognize that this patient has a sodium deficiency and decide what type of fluid to give.

But also, in terms of benefits… even when I look at our graduates who are already working, we see that they are doing great things. They are in various levels of leadership, they are involved in **policy development and decision-making**—they are the ones now **running hospitals**. If you look at nursing directors, ward in-charges—many of them completed this degree level. And they are performing well. Services have improved. I'd say that—truly, the degree has benefits.

Interviewer (I): Thank you [R3: Mmh]. Let's come to you, number two. [R2: Yes.]

Interviewer (I): [Regarding] the benefits?

R2: There must be benefits [I: Mmh], at every level. One is that we expect the degree nurse to have the ability to integrate—to seek information, convert it into knowledge, and convert that knowledge into understanding. But also, they are required to expand the **body of knowledge**—the nursing body of knowledge itself. It must grow as people gain knowledge and expand the **philosophy of nursing per se**—so that it develops.

You also expect that wherever a degree nurse is performing a task, the outcomes should be **significantly different** compared to when a diploma or certificate nurse performs the same task. That is, in terms of **quality**, in terms of **the foundation** of why something is done. The ability to **think** is expanded. The degree nurse becomes someone with greater focus, someone who can **integrate information**—and so, the care they provide to the patient will be of **higher quality**,

extraordinary, based on the knowledge they possess. They should not be just an ordinary nurse—not just a performer, but someone who acts **because of the knowledge they have**.

Interviewer (I): Mmh.

R2: So, it expands the **depth of thought** and the **nursing body of knowledge** itself as people advance through educational levels.

Interviewer (I): Thank you. Number one?

Respondent 1 (R1): Thank you. I think many things have been said about what the benefits of degree-level nursing are. They are significant. Because, in our country, we haven't had degree-level nursing for many years. We used to see the challenges that emerged before we had degree-holding nurses, and now that we have them, the difference is visible. There are major differences seen in the workplace, in areas where patients receive care. So, the benefits are apparent.

As education rises, we expect the **quality of service** to also change. So at the degree level, the benefits outweigh the challenges we face.

Interviewer (I): Okay, thank you very much. Number five, do you have any opinions regarding the benefits of the BSN program?

Respondent 5 (R5): Yeah, I'm not far off from the previous speakers. One thing I also see is that I believe when a student progresses from one level to another, they gain additional skills. And when they go out to work, we expect to see changes. For example, in hospitals—we ourselves are witnesses—when we take our relatives or when we go ourselves during hard times, when you are treated by someone who is a graduate—someone with a BSN—you notice the difference. Because that person provides care **holistically**, and that's how they were trained. They can treat your **physical issues**, they can provide **counseling**, and they can integrate the issues they've learned and put them into **practice**.

So, I think their **intellectual capacity** is also higher compared to someone with a diploma or certificate. So there are great benefits. Another thing I see is that when graduates are in hospitals, they add strength—**stronger manpower** than the lower levels, you see? So BSN holders have a **higher ability to think and make decisions** because they've been trained that way. And we—when we teach students—we teach them how to develop **critical thinking**, so that when making decisions, they can combine theory, practice, and deliver better outcomes. So the **quality of care** is higher for someone with a BSN compared to the other levels. So the benefit is significant, and they've also **added manpower** in hospitals, and we are seeing **major improvements** in those facilities.

Interviewer (I): Thank you. Coming back to you, number one—our graduates, the ones we train—we want them to progress further. Are our institutions prepared to give them the capacity to move on to higher levels, especially Master's programs?

Respondent 1 (R1): (Silent for a while) Let someone else begin first.

Interviewer (I): Okay. [R1: (laughs)] You're welcome. Let's move on. How do we prepare our students to have the ability, to have a greater chance to advance? Over to you, number three.

Respondent 3 (R3): From my perspective, in terms of knowledge, we have prepared them. Meaning, once they complete their BSN, they should be able to proceed to higher education. But I think we, as institutions, haven't established the **proper context**. What I mean is, we haven't created a conducive environment for further development. That's why you'll find that even the number of programs available at higher levels is limited. You never know—maybe they want to progress, but **where are the opportunities?** There are none.

Even scholarships are a challenge. Most of the time, they fund themselves. I don't know—only a few seem to get government scholarships. But even we, as universities, have **not made efforts** to seek out scholarships for students to continue to higher education. We should have a system in place—if we see a student has done well, let's **retain them** and help them find a scholarship so they can go further. That's what would help nurses.

Interviewer (I): Thank you.

R3: Truly, we're not well prepared—I must say that.

Interviewer (I): Number three says we're not well prepared to empower our graduates to advance.

R3: Especially in terms of **finance**.

Interviewer (I): Finance?

R3: Yeah.

Interviewer (I): Number four?

Respondent 4 (R4): Thank you. What we're doing at the undergraduate level to help them advance further is mainly teaching them in a way that enables them to **achieve GPAs** that will qualify them based on academic criteria. But when it comes to **finance**, we've not done much in that regard.

Also, even though we teach many different subjects during the degree program—and students might be interested in various areas—when it comes time to pursue further studies, many of the **Master's programs aren't even available**. Only now are we starting to see more—there's Midwifery, Mental Health, Pediatrics Nursing, and others. But earlier, it was tough—you wanted to study, yes—but the programs weren't there unless you were lucky enough to go abroad. And to go abroad—**where is the money**? Most people have to **rely on luck** to get scholarships or must become tutorial assistants so that institutions send them for further study. But if someone is working clinically or in a private facility, there's **no clear path** for them to further their education because there's **no support system**.

So overall, while we support students to get good grades, **when it comes to seeking scholarships**, we have **no proper strategy** in place to support them.

Interviewer (I): Thank you very much. Number five?

Respondent 5 (R5): Yeah, I'm not too far off from what's already been said. One thing we do as instructors is that when we're teaching students, we **tell them about Master's-level opportunities**—that they exist and that there's a chance to move from a degree to a Master's. We often encourage them—especially those who show interest. Some want to be instructors like us. Others have their own goals—someone might say, "I want to pursue this specific Master's degree." They'll come and ask you, "Teacher, if I want to do pediatrics, what are the options? Which course is better?"

They come to us for counseling, because we are **their advisors**, and when they come, we **guide them**. For example, we tell them, "If you want pediatrics, make sure you perform well in certain subjects that will help you get a good GPA for advancement." And even in the classroom, we tell them, "Don't just stop at your degree—go further." Because when you go further, your **ability to perform better** increases, and even **your salary improves**. We motivate them.

Now, the challenge, as we've said, is that many students **want** to progress, but **they can't afford the tuition**. On the other hand, institutions themselves **don't have enough instructors**. Mmh! You'll find that many universities offer only undergraduate programs—but they **don't have enough qualified faculty** to offer Master's programs. Most nursing schools are still **new**, and they **lack the capacity** to train at the Master's level.

Only a few offer Master's degrees, so when students go there, they still face the same tuition challenges. So the problem is **on both sides**: institutions are young and haven't hired enough faculty to support Master's-level programs, and students struggle with **funding**—even though the desire to study further is there.

Interviewer (I): Okay, briefly, if you have something to add?

Respondent 2 (R2): Ah... we've talked about environments and upcoming changes. In today's era of education, beyond just finances—one of the things we need is **preparation in technology**. If we were well prepared technologically, students could advance through **online programs**, some of which are even **free** nowadays.

But a big problem, even beyond the issue of whether colleges are prepared, is the **learner themselves**—they must master the **learning process** so that once they finish, they can **search for more knowledge** independently. That is, they can **self-learn**. Because today, it's impossible to finish school and say, "I'm done." No—you must continue learning.

So, every program should **empower students** to continue learning. And now there are different components—finances, technology, mindset, and creativity. For example: what if **WhatsApp** was used by nurses to expand knowledge? Imagine if nurses used it just to **share knowledge**—they would grow tremendously. Just using WhatsApp.

But the way WhatsApp is used today—does it **expand or deepen knowledge**? Does it help a student **learn through a social space**? Because nurses from different areas could **create social spaces**—maybe on Twitter—and discuss issues related to nursing and how to further their education and share opportunities. Imagine the number of **opportunities that could arise**—there would be so many. But are there **nursing social spaces** out there where you can say, "Indeed, this is a place where nurses from, say, the U.S., Canada, Tanzania, are all discussing an issue"? That kind of space would help them **expand** and **rise intellectually**.

But technology is here—and **our creativity** is lagging behind. Instead of using it to build knowledge, we're using it more for **gossip**, **rumors**, and **sharing explicit content** [Interviewer: laughs]. So it hasn't been used effectively to **expand knowledge**.

Interviewer (I): Thank you. Number one?

Respondent 1 (R1): Thank you. I see many issues have been discussed, but there's a challenge that number five mentioned—that **individualized guidance and counseling** for students isn't really happening. We have **large numbers of students**, and it's hard to get to know each one individually—to identify, for example, if one student would do better if guided toward a particular path, but they're stuck pursuing something else where they're not strong.

If we could guide each student personally, **progressing to higher education would be easier**. We went through our undergraduate studies, but we never had a lecturer say, "Faraja, I see you're doing well in this subject—push yourself and go further in this area." Or, "Juma, you're strong in this topic—here's the advantage and disadvantage if you pursue it." That's not happening.

So when students reach graduation, they start scrambling for scholarships—"Oh, there's one in mental health? I'm going there!" Or "So-and-so went there, I'll go too." Or they follow the **available slot**, not their **true interests**. That one-on-one counseling isn't emphasized enough. At the end of the day, students choose something randomly, **cling to it**, and later things get difficult.

So I think this area needs more attention.

Interviewer (I): Before I leave you [R1: Yes], what should be done so students can be properly counseled about available programs?

R1: Because we have these things called **academic advisors**, that's their role—to sit with students. And when you sit with them, you get to know each one's challenges. You can be an academic advisor for four years and never actually meet the students—or understand their struggles. Some may have **family problems** that affect their performance in certain subjects. But once that issue is understood, maybe they can succeed in a field you didn't expect.

So this academic advising role should be taken seriously. Teachers should be required to submit reports—like if a particular student needs to be talked to or advised on something. Or if a student couldn't express themselves in front of their classmates, they should be given **separate time** to

speak. Though, to be honest, **time is also a challenge**—because we have **too many students** to give them all the proper attention.

Interviewer (I): Thank you. Number three wants to jump in again.

Respondent 3 (R3): Yes, I'd like to add something. There's something called a **career pathway**. This career pathway should be something every student follows so that the advisor can help guide them—"Your career pathway might go this way." I think we've already seen this issue in nursing—someone goes and takes an MPH, and later they're told, "This isn't your career pathway." See? They went the wrong way. So these nursing career pathways need to have **clear components**. Some people went off and took unrelated management programs and came back to find, "This is not your career path." I think our nursing schools and universities need to have **offices dedicated to advising students** on these career pathways. So that even when you finish your degree, you know: "You can go this way, not that way." Otherwise, students lose time and money on paths that don't suit them.

Interviewer (I): Thank you. Now, as we prepare to review our undergraduate curriculum—how can we strengthen **critical thinking** in our students? Let's start with number two.

Respondent 2 (R2): To improve critical thinking—first, we must understand that for nurses, there's a **neurological aspect** to education. We need to know how **neurons develop**, and particularly how that affects **learning**. There's a connection—neurons and learning. To build critical thinking, we must incorporate **question development** into the curriculum.

What kind of **models** are we using to stimulate a student's ability to ask questions? When students are taught to question, they naturally start to **think critically**. In some countries, nurses work alongside engineers. For example, when designing a hospital building, a nurse is involved to ask, "Does this design allow me to care for the patient effectively?" Or in developing **AI tools**, the nurse must be there to ensure the machine doesn't compromise **holistic patient care**. That's why questioning is key—and must be part of the curriculum. The types of questions students are asked—if they only encourage memorization and regurgitation, they'll never develop critical thinking.

Interviewer (I): Okay, before I leave you—how do we build **problem-solving** and **creativity**?

R2: To develop that, you must expose students to **real-world scenarios**. The content we teach must be **scenario-based**, to prompt them to **think through solutions**. Instructors must shift from just teaching to **accompanying** the student—helping them through the learning journey. Creativity is about seeing the same thing in **multiple dimensions**—so students should not just see "a bag," but ask: "What else could this be?" We must build their ability to see **multiple perspectives**.

Interviewer (I): Thank you. We have a new participant—number six. Please help us: How can we equip our BSN students with **critical thinking**, **creativity**, **problem-solving**, and even **resilience**?

Respondent 6 (R6): Okay, thank you for the question. In their learning, we must use **methods that promote critical thinking**—for example, giving them **assignments** and **scenarios** that encourage them to analyze and make decisions.

Interviewer (I): Okay, thank you. Let's go deeper—we've seen critical thinking is important. What about **technology** and **digital literacy**?

Respondent 4 (R4): Before we go forward, let me add something. We talk about helping students think critically—but what about the **instructors**? We cannot move forward if we are weak ourselves. Before helping students, we must ask: **Do we have this capacity?** If not, we'll just ask them memorization questions like "What is nursing?" and they'll just memorize and forget. Also, when we send students to the **clinical area**, we teach them all conditions—but when test results come back, they say, "Wait for the doctor." The nurse should be able to **interpret results**. If the doctor is delayed, a patient might die while the nurse is standing by—unable to act. Nurses must be **empowered to act**, not just make care plans. Also, many degree-level nurses **lack resilience**. They say, "Ward work feels like slavery." We must prepare them early—no matter how educated you are, even a professor, if you're a nurse, your job is to **serve the patient**.

R3: Let me add something too—we must **engage students in problem-solving**. For example, the **nursing process** is a great tool to promote critical thinking. It helps the student assess the patient, analyze the problem, and plan care. I remember when I was a student, we'd go to the clinical area, apply the nursing process, and people would say, "Wow, things are improving here!"—but it was just us students. So let's emphasize **nursing process application**.

Interviewer (I): Thank you. Number two?

R2: Yes, we're still on critical thinking. Number one said—do instructors themselves have the **skills**? Do we even know how to ask critical thinking questions? I think to produce a student who is a **critical thinker**, a **problem solver**, we must **change our teaching approach**. We must shift from "teaching" to **facilitating**. Because when you facilitate, 80% of the learning is done by the student. With teaching, we just come in with 100 slides, deliver a lecture, and leave. But if you're a **facilitator**, you guide the student to **discover the topic themselves**. I had a lecturer like that—he never gave lectures, but we all prepared because we knew we had to **share ideas**. That style helped us grow.

Interviewer (I): Welcome number five

R5: Yeah, and he stole my point [laughs]. But what I wanted to say is: our curriculum must **reduce theory hours** and increase **clinical and seminar hours**. For example, if you have 100 hours, 60 should be for clinical/practical, and only 40 for theory. Competence cannot be gained in a classroom alone. We say our curriculum is **competence-based**, but competence means **skills and attitudes**—and that must be built in clinical areas. Teaching should be **interactive**, not just lectures. Right now, our classes feel like **church**—you come, listen, leave. No chance to ask the preacher questions [group laughs]. We must change that.

R3: After all, these students we're training—they're going out there to **solve problems**.

Respondent 2 (R2): Sorry—just to chip in. There was a point mentioned by participant number five, about **competence**. We often hear these two terms: **competence** and **content**. Nowadays, they've become buzzwords—people say, "We've moved from content-based to competence-based education." But these two approaches have their own **characteristics**.

For example, **content-based learning** is rooted in **behaviorism**—which uses policies of **reward and punishment**. You perform well? You get an A, B, C, or D. In primary school, it was corporal punishment. You get top marks, you're praised. That's content-based.

But **competence-based learning** is guided by **cognitive theory**, where the **mental relationship** between teacher and student matters. And the policy here is not about reward or punishment—it's about **feedback and self-reflection**. The **feedback** we give students is vital for learning—more so than reward or punishment.

One thing I cherish in competence-based learning is **mistakes**—when a student makes a mistake, it helps you, as the instructor, understand where they are and what they need. This leads to **student and teacher reflection**, and only then can you say this student is progressing toward competence.

So, singing the song "content-based vs. competence-based" sounds nice—but actually applying competence-based education takes **tolerance, patience, and deep engagement**. It's not easy.

Interviewer (I): Thank you. Before we finish this section—there's the issue of **technology, digital literacy, and information literacy**. Number four?

Respondent 4 (R4): Thank you. I think we've made progress. I believe the BSN curriculum already has an **IT course**, right? So through that course, even those who didn't know how to use computers begin to learn. But now, we need to look beyond that—because even in hospitals, surgeries are being done using machines, not traditional open methods.

So maybe we should consider **short courses**—like one-week programs to visit referral hospitals, just like secondary students go on field trips to soap factories. We could take nursing students to **Muhimbili, Mloganzila**, etc., to see how machines work.

And for hospitals with resources, they can enhance their **skills labs** with **digital tools** and **simulation models**—more advanced than the basic mannequins. Right now, we're not too far behind, but we can do more.

Respondent 1 (R1): Thank you. I think participant number four made a good point. But when it comes to **information technology and literacy**, we're still **limping**. Yes, we teach students IT, but at the end of the day, we just give them PowerPoint slides and expect them to navigate them.

We don’t build an environment where students are told, “Go to the library website, find this book, go to this page, read it, and return next class to summarize what you found.” That helps build the habit of **seeking information independently**.

Also, we sometimes **undermine students**—just because we’re the ones standing in front, we assume we know everything. That’s not true—some students know more than we do.

I remember a student at Msata who came up with an **idea for a system** to track students during clinical placements—who went where, what they learned, who supervised them. It was a great innovation!

Sometimes we’re too strict—standing behind students in the ward, intimidating them. If we gave them space, they could learn more freely. So we need to **create a mutual learning environment** between teachers and students.

Respondent 6 (R6): Just to add, nowadays many hospitals have **hemodialysis units**. Students should be given opportunities to visit such places. In our course on **medical and surgical nursing**, there are sessions about it—but due to limited resources, we often end up teaching just in the classroom.

Sometimes the students ask, “Madam, we’ve learned about this, but can we see real patients?” But at **general hospitals**, there may be no patients undergoing dialysis, or head injury surgeries, or cardiotomies. They want **real-life exposure**, and I think that’s important.

Respondent 3 (R3): I think we need to allow **elective studies**—not all students are financially struggling. Some can afford to choose their own learning paths. And I still insist that we rename the program to **“BSc Nursing with IT”**. Why? Because IT is a **core course**.

If IT is core, then why not highlight it in the title? Programs like “BSc with IT” are highly marketable. Let’s make it clear and valuable in the job market.

Respondent 6 (R6): Thanks—you reminded me of something. You know, “with something” has more **market value**. But many nurses feel **demobilized**. For example, someone brought up the issue of result interpretation—what comes next?

In nursing, there are **barriers** that limit us. In some countries, a BSN nurse can perform **some surgeries**. Not just circumcisions—but even **C-sections**. There was a proposal to allow this in Tanzania, but it faced fierce resistance.

Maybe this study could help us **break those chains**. Because sometimes, in health centers, nurses are alone, and lives could be saved if they were empowered to **intervene more directly**.

Interviewer (I): Thank you. Let’s move into the **last section**. You’ve all mentioned the importance of communication skills. One of the main criticisms out there is: “Nurses can’t communicate well.” In your opinion, how can we **strengthen therapeutic communication** in the BSN program?

Respondent 4 (R4): Thank you. Communication is a **cross-cutting competency** in the BSN curriculum. But how well is it being implemented? That's the issue. Sometimes we focus more on **technical knowledge** and **less on communication**. We end up with nurses who are competent technically, but who **lack interpersonal skills**. Patients say, "That nurse didn't talk to me," or "She was rude."

To improve this, communication training needs to be **embedded into every course**. It's not enough to teach it in just one semester. And we should include **role plays**, **case scenarios**, and **real-life videos** where students can observe and critique behaviors—both good and bad examples.

Students should also **practice with feedback**. For example, record a simulation and review it together. Ask: "Where could you have done better?"

Also, we should train them to communicate not only with patients but with **colleagues, supervisors, and interdisciplinary teams**. And we need to model this behavior as educators too.

Respondent 5 (R5): I agree with number four. What I'd add is that **communication begins with respect**. You'll find someone calling a patient "yule mgonjwa wa kisukari" ("that diabetic") instead of by name. Or talking over them like they're not there. So, we must nurture **a culture of respect**—using the patient's name, explaining procedures, asking consent. These are simple things, but they go a long way. We also need to **assess communication skills practically**. Right now, we mostly assess theoretical knowledge. But we should **evaluate students during clinicals** on how they communicate—with patients, family members, and colleagues.

Interviewer (I): Thank you. Now, as we close—what would you like to see **changed or improved** in the undergraduate curriculum?

Respondent 1 (R1): We need to increase the number of **practical hours** in clinical areas. Competence is gained by doing. The curriculum says we are training for competence, but how can that happen if students spend **more time in classrooms** than in clinicals? Also, we should **review all courses** and ask: What skills should a BSN graduate have? From there, we can map the curriculum to ensure those skills are built throughout the program—not in just one semester.And we need to reduce redundancy—some topics are repeated in different courses. Let's be more efficient and **streamline the content**.

Respondent 6 (R6): Let's also rethink **evaluation methods**. Right now, we rely too heavily on written exams. But real nursing requires **hands-on skills**, **decision-making**, and **empathy**. We need to assess students in those areas too.

Respondent 3 (R3): I agree. And I'd add that the curriculum should prepare graduates to work at **all levels**—from a rural health post to a national referral hospital. That means they need **broad clinical skills**, not just theory. Let's make our curriculum **flexible**, **responsive**, and grounded in **real-world practice**.

Interviewer (I): Okay, we've reached the end. Last question: If you were to advise the university, what Master's programs should be prioritized for nursing?

Respondent 1 (R1): I think Maternal and Child Health Nursing is very important. Because we have a big problem with maternal deaths and child deaths.

Respondent 2 (R2): Yes, I would add Mental Health Nursing. This is a very neglected area. We really need to prepare more specialists.

Respondent 3 (R3): I support what's been said. I also think we should have a Master's in Pediatric Nursing. Children require special care.

Respondent 4 (R4): We also need a Master's in Infection Prevention and Control. With the experience of COVID-19, this is a high priority.

Respondent 5 (R5): Let's add Advanced Nursing Practice. This will enable nurses to work independently, especially in areas with few doctors.

Respondent 6 (R6): I'd also recommend Nursing Education and Leadership, to train future educators and nursing managers.

Respondent 1 (R1): Even specialties like Cardiology or Neonatal Nursing should be considered. We are seeing more of these cases.

Respondent 3 (R3): And Oncology Nursing. Cancer is increasing, and we need trained personnel.

Respondent 2 (R2): Let's prioritize based on national needs. But also give room for people to pursue their interests. That way, we'll have diversity in the profession.

Interviewer (I): Thank you. Now, there was another question here that asked what we should consider—I think we've already addressed it as we talked about establishing those programs.

Participants: Mmmh.

I: Well then, I think that brings us to the end of our discussion. We thank you very much—it has been great, and you've given us excellent cooperation. We pray that God blesses you greatly.

Participants: Amen, thank you.

I: Thank you so much. If there's anything else to add, perhaps?

Participants: Nothing.

I: Nothing? Thank you very much.
[Participants laugh]

FGD Educators #2

Interviewer (I): First of all, we would like to know your opinions regarding the bachelor's degree program in nursing. Anyone may begin sharing their views.

Respondent 3 (R3): My opinion regarding the bachelor's degree in nursing is that it is a very important degree, especially considering the national needs and responsibilities—particularly the goals related to learning outcomes and objectives. As educators and trainers, we believe it's crucial training that needs ongoing improvement to keep up with national and global needs. Since most of our graduates go to hospitals where there are emerging diseases and even recurring ones, plus the growing awareness of the people we serve, I believe nurses must always have up-to-date training. So this is a vital profession for the health sector and for the broader development pillars of both the country and the world.

Interviewer (I): Thank you, another participant?

Respondent 1 (R1): Thank you for the opportunity to participate. My opinion about the nursing curriculum at the bachelor's level is that we expect strong output from our students who go on to work in hospitals. But as we all know, there have been developments in science and technology. Nowadays, the way we deliver services is very different from how it was in the 20th century or even ten years ago. So, when we design or review our curricula, we must consider things like technology. We need to include courses that are aligned with current realities. For example, courses that teach nurses how to operate hospital systems like informatics, or how to handle advanced life support machines and modern diagnostic tools. So when training our students, we must consider scientific and technological advancements so that they can keep up.

Interviewer (I): Thank you. Along with what you've shared, we'd now like to know your thoughts about the **benefits** of the nursing bachelor's training. Do you think there are any benefits, and if so, what are they?

Respondent 4 (R4): There are many benefits. One of them is that we are **reducing the gap** of health professionals in the healthcare sector. We're also contributing to reducing **unemployment** in the country due to the **multiplication effects** that come with having skilled health workers in nursing. We're also reversing the **trend of disease burden**—because when you have professionals who can support health service delivery, the country is in a good position. Moreover, the nursing sector is closely related to **other sectors**, like tourism. Right now, we see that the tourism industry is growing rapidly. But people won't travel to a country where they can't be assured of **reliable healthcare services**. That's where nursing graduates come in—they're part of that bigger picture. Today we see many **privately run clinics**, and most of the management is done by **nurses**. The doctor comes in, performs their duty, and leaves—but it's the nurse who **continues to run the facility**. Having graduate-level nurses really helps in such settings. And when we talk about **national indicators** like **maternal mortality**, now we're beginning to see doctors in remote areas. But for the most part, it's nurses—especially in **lower-level facilities**—who are doing this work. So, when we have a nursing graduate there, we feel **assured of quality services**, and the community gains **more confidence in their government**.

Respondent 2 (R2): Okay, to add to what number four has said—another benefit is that we'll be able to **care for patients more professionally** and competently. This also contributes to the **economic well-being** of the country. Why? Because when patients receive **appropriate treatment quickly**, they **spend less time in the hospital**, recover faster, and **return to their national duties** more efficiently. That helps both the **family economy** and the **national economy**.

Interviewer (I): Thank you very much for your thoughts. Any other participants?

Respondent 1 (R1): Thank you. There are **many benefits** to producing nursing graduates. One of them is that when we train a student as a **nurse officer**, upon completing their studies, they can take on **leadership roles**. In most of our hospital settings, we rely on them to become leaders—heads of departments, units, and even **health committees**. These are people who can **speak on behalf of patients**, make suggestions to various stakeholders, and their input can actually influence change. That's benefit number one. Benefit number two is **skill level**. When we graduate a nurse with a bachelor's degree, we expect they'll bring something **new and different** compared to a diploma holder. Their **efficiency and performance** in delivering care will be higher. So, we improve the **quality of nursing services**. Another thing is that life is constantly changing. **New diseases** emerge, and we need people who can do **research**. When we train nurses at the bachelor's level, they can **participate in research** and help generate **new findings** to solve real problems. They can also **reach out to the community** through **outreach programs**—educating people and offering preventive care. These are services often done by degree-holding nurses because they have that **additional training and knowledge**.

Interviewer (I): Thank you. Number two?

Respondent 2 (R2): Yeah, number two here… [laughs with the group] There are many benefits, and I'd prefer to break them down by **levels**. First, at the **individual level**, empowering someone to reach the bachelor's level fulfills a **basic human need**—it makes them feel like they **belong** and are contributing to the profession. It boosts their **self-esteem**. Back in the day, a **diploma was the highest level**, so having a degree makes people feel proud. It also improves **income**. When someone gets a job with a degree, they may earn more than someone with a diploma. So, it improves their personal and family economy. Also, when exposed to an **advanced level of education**, individuals can connect with **other professionals and scientists**—even outside the health field. Then there are benefits at the **professional level**. The nursing profession can now take pride in having gone through a **positive academic transformation**—from certificate, to diploma, to bachelor's. It helps people **advance in their roles**. Nationally, now that we've **decentralized health services**, we need **bachelor-level nurses** who can **work independently** in health facilities and **in communities**—especially where **preventive care** is the priority (primary, secondary, and tertiary levels). So, having nurses with degrees helps the country provide **cost-effective**, **quality care** at both community and facility levels.

Interviewer (I): Thank you very much. Now let's move on to another area: We often train students at the undergraduate level, but they finish and don't move forward to the Master's level. In your

opinion, do our institutions prepare students well to proceed to **postgraduate studies**? And how can we improve this?

Respondent 4 (R4): Thank you. Honestly, we try our best to give students a **foundation**, especially through courses like **research methods**, so they're able to move on to **Master's-level programs**. But if we're honest, it's not just about the curriculum—we also need to have **mentorship and guidance systems** in place. You'll find a student finishes their degree and has no idea what programs are out there, or what to do next. Most of them don't even know **where to start** looking for a Master's program. So institutions need to have **career guidance offices** or assign **mentors** who can guide students about further education—both locally and abroad. That way, they're not lost after graduation.

Interviewer (I): Thank you, number four. Number two?

Respondent 2 (R2): I agree with number four. We also need to ask: Are we just **finishing the syllabus**, or are we truly **preparing students** to move forward? If we're just racing to finish the syllabus, the student leaves with knowledge but no **sense of direction**. Institutions should not just **teach courses**, but also **help students identify their strengths**. We might have someone who is very strong in community health or maternal care—why not guide them in that direction? Sometimes you find a student is pushed into a field they're not even passionate about, simply because that's what's available. So we need to introduce **career mapping** at the undergraduate level. We must also train **academic advisors** to do more than register students for exams—they should have **active conversations** with students about **what comes next**.

Respondent 1 (R1): To add on that—we should also include **course units or modules** that are specific to preparing students for postgraduate education. Even a simple **elective unit** that walks students through **postgraduate program options**, **scholarship applications**, and **academic writing** could be very helpful. We also need to **nurture talent early**. For example, if a student is strong in research, why not assign them to work with a faculty member on a project, so they start building confidence and interest in that area? That would make it easier for them to apply for Master's programs.

Interviewer (I): What are the **barriers** that make it hard for graduates to progress?

Respondent 2 (R2): The main one is **financial limitation**. Most students simply can't afford to pay for postgraduate education. We need to **expand access to scholarships**, and institutions should take the lead in **partnering with donors** or **creating internal funding programs** for top-performing students. Secondly, there's a **lack of information**—many students don't even know where to apply, or how to write a statement of purpose, or what academic references are. So even if they qualify academically, they're unable to prepare a competitive application.

Respondent 3 (R3): I'd like to add that we need to **build partnerships** with both local and international universities. That way, we can have **clear transition paths** for students who want to pursue Master's programs. Imagine a system where we say: "If you graduate here with a GPA of X, we've already got an MoU with this university, and you qualify for their Master's in Mental Health Nursing." That kind of system would **motivate students to excel**.

Interviewer (I): Now that we've discussed postgraduate studies, let's talk about something important within the undergraduate level. How can we strengthen **critical thinking**, **creativity**, and **problem-solving skills** in our students?

Respondent 3 (R3): Thank you. I think for students to develop critical thinking, we must **change the way we teach**. We need to move from **teacher-centered** approaches to **student-centered** ones. For instance, instead of delivering 100 PowerPoint slides in a lecture, we should pose **real-life clinical scenarios** and ask students to work through them. We give them a case: "A patient comes in with this and that... what do you do?" That way, students learn to **think through problems**, not just memorize content. Also, we need to **train the trainers**. If lecturers don't know how to stimulate critical thinking, how can they pass it on? Some instructors still teach the way they were taught 15 years ago, using rote memorization. That has to change.

Interviewer (I): Okay, number two?

Respondent 2 (R2): Yes, to build **problem-solving**, we should integrate **competency-based learning** that's structured around the **Nursing Process**. That tool is brilliant. It guides students to assess, analyze, plan, implement, and evaluate. When used well, it trains them to **make decisions based on evidence**—not emotion or assumption. Also, we can introduce **open-ended assignments** that have **no single answer**. Let students debate different approaches. We should encourage **group work**, **reflection journals**, and **peer teaching**—these are methods that develop **independent thinking**. We must also make space for **failure**. When students make mistakes, we should let them reflect and try again, instead of punishing them. That's how growth happens.

Respondent 4 (R4): I'd like to add that critical thinking isn't just about academic ability—it's also about **confidence**. Some students are shy. So we should create a **safe learning environment** where they feel comfortable speaking up, asking questions, and even challenging ideas. We can use **role plays**, **debates**, and even **simulation labs** to encourage creativity. Let students experience pressure, then reflect: "What could I have done differently?"

Respondent 1 (R1): I agree with all that's been said, but we should also look at **the feedback we give students**. If we only say "correct" or "wrong," they won't grow. Feedback should be **constructive and specific**: "This part was strong, but consider this next time…" Also, the way we **ask questions** in exams and classwork needs to change. Instead of asking, "Define this," we can ask, "Given this patient case, what are three possible actions and why?" That encourages **analysis and reasoning**.

Respondent 5 (R5): Thank you. One thing I always say is: **creativity is not memorized**—it's triggered. You can't tell students, "Be creative." But you can **create an environment** that pushes them to think differently. In my classes, I sometimes ask a question that has **no clear answer** and let students work through the ambiguity. At first, they're confused. But over time, they become more **confident** in navigating uncertainty. That's real-world thinking. We also need to expose them to **interdisciplinary challenges**. For instance, how would they work with an engineer to design a better patient bed? Or a tech team to create an app for tracking medication adherence? These are opportunities for **real creativity**.

Interviewer (I): Thank you so much. That was very rich. Now let's shift gears: How can we strengthen **digital literacy** and the use of **technology** in our BSN program?

Respondent 2 (R2): This is an important area. Technology is moving fast, and the health sector is transforming with it. So, students need to be digitally literate—not just how to use Microsoft Word or PowerPoint, but how to **find information**, **evaluate sources**, and use digital tools to **solve real clinical problems**. It's not just about using computers—we need to train them on **how to use tech to learn**. That includes accessing **online journals**, using **health databases**, and **navigating e-learning platforms**. And these skills should be taught **across all courses**, not just in a one-time IT class.

Respondent 3 (R3): I want to add something important here. We often give students lectures using PowerPoint, yes—but we rarely challenge them to go and **find out more on their own**. For example, we could say, "Here's a topic. Instead of me teaching it, I want you to go to **PubMed**, read two articles, and present a summary." That builds both **critical thinking** and **digital research skills**. Students also need to learn how to use tools like **Zotero** or **Mendeley** to manage references, and how to write **literature reviews** using proper citations. These are **essential postgraduate skills**, and we should introduce them early.

Respondent 4 (R4): I think we're also underutilizing **video content**. There are amazing medical and nursing tutorials on YouTube and open access platforms. Why don't we **integrate curated video content** into our teaching? A student may forget a lecture, but they'll remember a good animated video showing how the heart pumps blood. It sticks! We can also encourage students to create **digital content**—let them make presentations, infographics, even short educational videos. That's how they'll retain knowledge.

Respondent 5 (R5): Let me go even more grassroots here. WhatsApp! Yes—WhatsApp can be a powerful **academic tool**. Students already use it for social communication. Why not guide them to create **discussion groups** that focus on clinical case reviews, or share learning resources? I once had a class where we used WhatsApp to **run weekly clinical questions**. I'd post a scenario, and students would respond with ideas, references, and their reasoning. It became a dynamic and collaborative learning space.

Respondent 1 (R1): I love that. But we also have to teach **etiquette** and **professional use of digital tools**. Sometimes students join online discussions and post irrelevant content, or plagiarize from Wikipedia. We must instill **digital responsibility**—not just the skills, but the **ethics** of using digital tools in a healthcare context. And another thing—we must **model** this ourselves. If we, as educators, don't use these tools well, students won't take them seriously. So, let's **lead by example**.

Respondent 6 (R6): I think we should also consider field exposure. If possible, organize **digital health field visits**. For instance, visit a hospital that uses **electronic health records (EHRs)** or **telemedicine systems**. When students see these systems in action, they understand how IT and nursing intersect. That practical exposure is better than a hundred lectures.

Interviewer (I): Thank you all for your rich input. Let's wrap up with a few final questions. First—**communication** has been identified as a major challenge among practicing nurses. How can we strengthen **therapeutic communication** in our BSN program?

Respondent 3 (R3): Communication is **key in patient care**. It's not just about saying words—it's how you **deliver information**, how you **listen**, how you **respond**. We should make therapeutic communication a **cross-cutting theme** in our teaching—not just a one-time course in year one. Let's integrate it across multiple modules and use **practical sessions**. For example, through **role plays**, **simulations**, and **video reviews** where students can see both good and bad communication and reflect on it.

Respondent 2 (R2): We should also **assess communication skills practically**. Not just in exams, but in the clinical setting. For example: Did the student greet the patient? Did they introduce themselves? Did they explain procedures clearly? We need to create **rubrics** for this and ensure supervisors assess students not just on technical care, but also on **how they engage with patients**.

Respondent 4 (R4): I agree. Some of our students are very knowledgeable but struggle to **empathize** with patients. Others don't know how to handle difficult conversations—like delivering bad news. We should offer **communication labs** to practice these scenarios in a **safe space**.

Respondent 6 (R6): It starts with us too—as educators. If we model poor communication, students pick that up. We must teach by **example**, especially in clinical rotations.

Interviewer (I): Now, as we consider **curriculum review**, what would you suggest changing or improving in the BSN program?

Respondent 1 (R1): First—**increase clinical hours**. Right now, we have more classroom time than practical time. But nursing is **learned by doing**. Let's reverse the ratio: 60% practice, 40% theory. Also, let's reduce **repetition** in the syllabus. Some topics are taught in multiple courses unnecessarily.

Respondent 5 (R5): We should **update course content** to reflect **emerging global health issues**—climate change, pandemics, digital health. These aren't in the curriculum, but they affect nursing practice now more than ever.

Respondent 3 (R3): Let's involve **stakeholders**—clinical nurses, policymakers, and even former students—in curriculum development. They'll tell us what's working and what's not.

Respondent 4 (R4): One more thing—**evaluation**. Let's not rely only on written exams. Use **OSCEs**, **practical assessments**, **portfolios**—tools that actually measure skill and attitude, not just memorization.

Interviewer (I): Thank you. To close: If you could recommend specific **Master's level nursing specializations**, which ones would you prioritize?

Respondent 1 (R1): Maternal and Child Health Nursing—especially to address high maternal and neonatal mortality in rural areas.

Respondent 2 (R2): Mental Health Nursing—we have very few specialists in this area, yet mental health issues are rising.

Respondent 3 (R3): I'd push for **Pediatric Nursing**. Children have unique needs, and general nurses don't always feel confident caring for them.

Respondent 4 (R4): We need **Oncology Nursing**. Cancer cases are increasing and most nurses are not equipped to handle the complexity of that care.

Respondent 5 (R5): Advanced Practice Nursing (Nurse Practitioner). It's time to empower nurses to **diagnose and prescribe**—especially in remote areas.

Respondent 6 (R6): Infection Prevention and Control—absolutely essential post-COVID. We need experts who can lead in this area across the country.

Interviewer (I): Thank you all. That marks the **end of our discussion**. You've been incredibly generous with your time and insights. May God bless you.

Participants: Amen. Thank you.

I: Thank you again. Any last thoughts?

Participants: No, nothing more.
[Laughter in the room]

Employers

Interviewer (I): Thank you for coming. As I mentioned earlier, I'd like you as leaders to give me your experiences working with graduates from Hubert Kairuki Memorial University. Number five, I see you want to contribute.

Respondent 5 (R5): Yeah, let me say I have almost ten years of experience working closely with students from Hubert Kairuki University. I began gaining this experience while working with them on the ward, but then I got experience supervising them as a leader. I can say that these are students who—because as you know our institution receives products from various universities—but I can say that we as leaders overseeing the nursing and midwifery professions are proud that most of them meet the standards especially when we're with them in clinical practice. A real example is our leaders who we allow to leave here and go study at this university; when they return, they become deliverable personnel who contribute positively to the department of services. You'll even see in this panel we have graduates from Hubert Kairuki who are already in management. This confirms my statement that this university is credible in terms of skills, knowledge, and even administration matters.

I: Thank you. Anyone else who would like to contribute on this? Okay, number three.

Respondent 3 (R3): Thank you. First, we are thankful to the university itself for offering these courses—they've been very helpful. I am one of the graduates of Kairuki University. When I went there to get an education, first of all discipline was a must. So, you've maintained discipline very strongly to the extent that even when I returned to the site, the students who were coming had very good discipline, starting from where they were produced. They were very attentive—most of them, though not all. But we really appreciate the leadership because we have had leaders who are attentive even when we consult them about student conduct. For example, there were students who wore inappropriate clothes, so we would send them back and also punish them. We would also monitor that if they didn't meet a certain percentage standard, they were not allowed to sit the final exam. So discipline was at a high level, and also productivity—some of the students have become very good in-charges, so it's going well. We congratulate you.

I: Thank you, number three. Anyone else who would like to contribute? Number seven, I see your hand.

Respondent 7 (R7): I don't have much experience with Kairuki students, but for the short time I've worked with them—first, they are attentive and punctual in terms of reporting to work. They arrive early and follow the instructions they are given. The challenge I've seen is the rotation time they're given because I too have had rotation, so I know—the time is short when you look at the things they are supposed to learn. If it's possible, they should be given more time to stay in the clinical area so that when they move forward, they find it easier to master many things.

I: That's for students—if I understood you well. How about the graduates you work with? What have you observed?

R7: For the graduates, there's no challenge.

I: Anyone else who wants to contribute regarding the Kairuki graduates you work with?

Respondent 1 (R1): On that, I won't be far from what number seven said. For me, I haven't been able to know who is from Kairuki or from where so that I can comment based on your question. Because I've been in this leadership position for now going on six months, and those under me are a bit many—about four hundred and something—so I haven't been able to know who is from Kairuki and who isn't.

I: So you'll need a bit more time. Thank you. Number four.

Respondent 4 (R4): I'm not far from the others. I've held some leadership roles—at first I started as an in-charge then progressed—but I've worked with people who studied at Kairuki, including participant number three, with whom I worked when he had just graduated from Kairuki. Honestly, they are good—their discipline is good, they are hard workers, and maybe we're here because of the good things he delivered which led him to where he is. In my current team, I have in-charges who studied at Kairuki, and truly they are dedicated and know what they're doing. Someone can't know what they're doing unless they were taught and given a good foundation. On the student side, I've had the opportunity to work with them a few times when I was a leader and their senior, and I can say they are obedient and they grow as number five said—they know what brought them to clinicals. Also, the good collaboration of teachers during follow-up is one of the things that made them who they are because when you send a student to a clinical area, we who are around them don't know them as well as their teachers do at school. So when teachers follow up, asking how their students are doing, sometimes requesting feedback or asking about challenges—it gives us, who are working with them, the room to help and guide those students. That's all from me.

I: Before we continue, anyone else who'd like to contribute? Number eight there.

Respondent (R8): Thank you very much. As my colleagues have said, my experience with the students is that they are proactive—they know what they're doing, they're eager to learn, and they come having already studied the theory. So when they come to the practical site, they've already studied the materials—unlike students from other institutions who often don't understand the theory part. So we find ourselves having to teach them differently, whereas Kairuki students already understand many things.

I: If I understand you, I think we need to emphasize more on students, but maybe as we continue, let me ask: what are your opinions about these graduates from Hubert Kairuki—are there any traits, any gaps in their skills? Do you see any differences between them and graduates from other institutions? Let's start with number five.

R5: First, from my experience, it shows that students from Hubert Kairuki University—I can say about 70% come from at least economically stable families. Because the university is private and its tuition is high compared to other universities, it already justifies that economically, they are stable—compared to others who get chances to study directly in government colleges. From the

work perspective here, we see them as people who are not troublesome. They come from families that have already solved minor challenges that can disturb a worker from achieving their goals due to family issues.
However, from my experience, a few of them are delicate.
As we’ve already contributed—if you look at an institution like this—what I know is that here we have about seven ward in-charges, a manager, and two in-charges in maternity if I’m not mistaken. That’s not just something we’re saying; that is part of the justification. Because when leaders are chosen, we don’t look at which school they came from—we look at their capability. So the picture I’ve painted shows they are trusted and understand what they’re doing.
But as I said, these are people who were brought up—right from their roots—with fewer basic issues that could disturb them from fulfilling their responsibilities. Many who come from there don’t have such challenges. In short, that’s what I can say.

I: Still on that, do you see any difference between those from Kairuki and other colleges—and if so, what’s the difference?

R5: The difference is—starting from the student level—a person we say is good depends on how they were treated back there. So when students come here, and their teachers come to collaborate with professionals who are the main supervisors—and considering we have clinical instructors in every area—when you get teachers who reach out to clinical instructors and discuss together the challenges their students face when they come here, it’s an undeniable truth that those challenges get solutions and are worked on as the students continue with their rotations. In the end, they are likely to get at least 80% of what they expected to gain. That’s when their quality becomes visible. In short, the secret to this success lies in the system used.
Still, we know there are challenges. These include:

- Getting information during exam periods about how to prepare supervisors—that’s a big gap.
- Getting late notice that students are coming tomorrow when we are not prepared to receive them.
- Insufficient time—we know when talking about nursing or midwifery here, you’re talking about... yes, in class they need knowledge—but when employed, we say a nurse is good first if they are good at skills. Then we can listen to them in discussions to understand their capacity.
  So we would love it if clinical hours could be increased.
  Second, the college should recognize the clinical instructors mentoring their students here onsite—because this is a very important part.
  Having a clinical and mentor forum with the university would make it much easier.
  Lastly, improve the type of exams. The teacher provides knowledge, but at the end of the day, the exams should reflect practical knowledge. So we say the university should base more on OSCE exams instead of putting too much theory which, in reality, after graduation, theory plays a small role while practicals play a bigger one.

I: Thank you very much number five. Anyone else want to add?

All: *Silent*

I: Maybe continuing on—what are your thoughts on this bachelor's degree program in nursing? What do you see as the difference between a nurse with a degree and one with a diploma? Because you are senior leaders and have worked with both in your roles. Number seven.

R7: Thank you. Briefly—it's very beneficial. First, it aligns with the market compared to how other professions grow—it's a must for professions to grow.
So having a degree has enabled us to grow professionally—now we've reached the master's level and we'll reach PhD. If we didn't have degrees, we wouldn't have progressed.
Second, in terms of knowledge—it has helped us to have broader knowledge aligned with the needs of the profession, society, and the world at large. We can now market ourselves globally.
Another big thing is it has increased the prestige of our profession—now we can sit at the same table with others who recognize that our profession is also growing and comparable to others.
Also, in service delivery—it has helped because this nurse has received extensive knowledge, diverse experience, and has been empowered. They can improve patient care and bring positive outcomes.

I: Welcome, number five.

R5: To add on—alongside what number seven has said—the most important thing is that right now everything in the world is about evidence-based and standards.
We know that in other educational levels—diploma, EN—they may have sufficient skills and even more experience than degree holders. But honestly, justifying the decisions they make and organizing and explaining *why* they do things—degree level gives people the confidence to do things knowing the rationale behind it. That's what I wanted to add.

I: Thank you. Welcome, number four.

R4: I wanted to add—this degree, unlike other levels as my colleagues have said, has helped increase decision-making ability.
Before, we relied on other professionals to make decisions.
Now we can at least sit at the same table—as we were once in the same classroom.
Sometimes there are things we share—like the confidence to say, "This shouldn't be done like that because of one, two, three… I won't give this today because…"
That has increased because of the degree level.
Decision-making was something nurses lagged behind in. We were being ordered around on everything.
But now, as education increases to the degree level and beyond—it has empowered us to make decisions.
Besides that, we've gained respect as professionals—people now recognize that this is a profession with its own education.
So we can sit at the table with others who also recognize their own professions.
I represent that.

I: Aha! Number three, welcome.

R3: We have gained the ability to do research, various types of research, and to submit these evidence-based practices and to oversee that this thing is done this way. I have done research—during Diploma we didn't have the ability to do research, and writings couldn't be published in journals and everywhere. Now we have the ability to write these writings and present them on various panels.

I: Thank you. Number two, welcome.

R2: Thank you. To contribute there, I'm not far from my colleagues, but when looking at service delivery, honestly it has improved because the knowledge these degree holders acquire is greater than the others'. Because they can make more decisions and can sit at the same table and decide. Even if they haven't reached the point of making the final decision, they can start doing something even when the doctor is present, then he can speak on it.
Another thing is the income—there's a big gap between diploma and degree holders.

I: Let's continue. You as managers—how should HKMU enhance its employment opportunities through this Bachelor of Nursing program? What do you suggest should be done? Number six.

R6: For that question, like other contributors have already said in some of the previous questions—from the teaching at the university—it has mostly been classroom-based. Therefore, they should increase time in the clinical areas where they meet different clients. So, if there are more clinical hours and fewer classroom hours, depending on how the curriculum will be designed, what they get in class and spending more time on site will allow them to link the two. In the end, it will produce a better product than the one who spends too much time in class and little in the clinical area—because you end up not applying what you learned, it stays in your head, and later in the market you fail… you have knowledge, but transferring it to skill is lacking.
We know when they go to various hospitals for placement, not everything they learned in class will be found there. But by expanding the scope of exposure based on what they studied, they will meet most of those things—so when they come to work, it'll just be a matter of topping up what they came with from school.

I: As number six said, as leaders, what qualities do you look for?

R3: Our training should be competence-based. That's when we can take over the job market.

R2: I think more courses should be added—let's not rely on BSN alone. We should have critical care and different courses because it appears you're producing good products. The nursing market is still very big. What kind of course? Right now, they don't just need BSN. We should think outside the box.

I: If I understood you correctly, we should increase postgraduate degrees. Let's go to number four.

R4: I'll go back a little to the point number five made about the qualities of students from HKMU. Most of the time, they have confidence—perhaps because of their background. You find

that the majority come from decent families.
But another thing they need to learn—you know, confidence also has a place. You may come from a good background, with a good education, but when you enter our work environment—it's very different from what they are used to.
If you're used to working in air-conditioned rooms, but get employed at Mwaisela where there are many patients in bad conditions—that kind of student may not be familiar with this situation as compared to someone from MUHAS.
So if you could help adjust their mindset and prepare them psychologically to understand that environments differ, it could help them more.

I: Thank you number four. Let me ask—you're here, what qualities do you look for when hiring a nurse with a bachelor's degree? Number six.

R6: Thank you. For that question, because this is a public institution, nowadays all jobs come from the government's employment secretariat. Though in the past, institutions used to recruit directly, now many public institutions no longer hire directly—except for volunteers. Those who are officially employed come through the recruitment secretariat.
So there are certain qualifications—they apply and are later posted. So we as a hospital have reduced say in determining specific criteria like before.

I: From your perspective, if you were asked—because here we are leaders and may go elsewhere later—if you, number six, were asked to give criteria, what would you suggest?

R6: For any nurse, we know they must be registered with the legally recognized council and must have a valid work license. Other things, like experience, or what they did at school, can follow. Looking at the school might cause bias, but the nursing qualifications—which already have guidelines like: "Nursing Officer must have these qualifications," "This nursing rank has these qualifications"—those are the ones you should walk with. Outside of that, if you add your personal preferences, I think that would be extra.

I: Thank you, number six. Welcome, number five.

R5: To add to what number six said—first, employment is competitive, so it's a struggle for the fittest. The better a student was prepared, the more likely they are to get a job. Because it's about competition—there's oral, written tests, knowledge will count.
In oral exams, people will check your communication skills, how you handle pressure, how disciplined you are, and how well you can position yourself. These are communication skills learned in school. That's where a person learns to stand before people and defend themselves—and eventually get hired.
But for HKMU to be a leader in the job market, what I know is: you must understand that Tanzania is a poor country. The people being served are poor—some don't even understand Swahili well. Some come to the hospital and don't even have fare to return home.
So if they are trained with this awareness and are built with the spirit to adopt this mindset—that "when I graduate, my market is to help Tanzanians of this design"—then it will be easier for them to get jobs.
Because even with their high education, they will humble themselves and help that Tanzanian

who doesn’t even have 50 cents to offer.
And every time a patient is left with that nurse, they’ll say, “when that nurse is on shift, there’s no problem.”
And then people will say, “Where did they study? Kairuki.”
“That person studied with so-and-so who is also good.”
That’s how the product becomes sweet to mention.
Unless otherwise, in our Tanzanian setting—you may speak fluent English and have studied at St. Francis with great qualifications—but when you meet a Tanzanian from Tandahimba, if you don’t understand the context you’re working in, you’ll end up discriminating, feeling stressed, and what you expected isn’t what you find.
In the end, many run from practice and look for NGOs—and eventually you produce people who end up in NGOs, who you can’t justify are helping the nation by being at the bedside and making an impact.

I: Thank you, number five. Now that we’ve reached this point in the discussion, let’s continue. We are reviewing the curriculum. As stakeholders—let’s say you work with these people—what skills do you want us to emphasize as we review the curriculum? Welcome, number five.

R5: Let me contribute. Despite all the good traits we’ve discussed, there’s a small percentage—two or three percent—but most are good.
But if you find even one student—I personally witnessed an intern from Kairuki, and it was a girl—but she was smoking marijuana.
That is a feedback I want to give.
We once received an intern and encountered such a scenario. So I think a person can be the best performer in class, but if there’s no mechanism to scrutinize and understand their internal behavior and what they do—it can slightly damage the image.
For example, that university is based on faith—we don’t expect someone to study for four years and no one ever noticed they’re a marijuana smoker.
And if that person is treating people—they could cause trouble and damage the institution’s image.
That’s what I can comment about that.

I: Thank you, number five.

R2: There’s **nurse etiquette** that’s being forgotten by some students—their appearance, how they dress, how they style or cut their hair, how they walk, their nails and such things.
There are things—sorry to go back—there was once an announcement that nurses should not wear fake eyelashes. Some said, “Oh, don’t make nurses too rough.”
But you’ll find a nurse with a “punk” haircut and thinks that’s okay. So we should be strict on nurse etiquette.
Also, IPC (infection prevention and control)—most of those I meet, their IPC is a bit lacking. I don’t know how you’ve put it—please address that too.
That’s all I wanted to add.

I: Thank you very much. Welcome, number seven.

R7: Maybe to add on—another thing is about laws, rules, and guidelines of nursing.
Many nurses seem not to understand them. And this subject hasn't been prioritized much in schools.
At the end of the day, when you're in the field, you're trusted to know—and if you don't, the law will catch up with you.
In this area, we should strengthen it so we get a product that understands what to uphold in the field.

I: Does that align with ethics?

R7: Yes.

I: Thank you very much, number seven. Maybe before we continue—number two, I see you have something to add.

R2: Let me contribute—about the students from that university—there's a simple question you can ask them the first day they arrive.
Ask, "What made you become a nurse or a midwife?"
You'll get many different answers. From there, you can already tell whether this student will become a good nurse later or is just passing through—somewhere along the way, they'll stray.
If you ask and get their answer early, it's easier to change that person—because some will say, "TCU chose this for me, I didn't want it."
So when they finish nursing and go to the field—they won't want to mop floors or provide basic care to patients.
It doesn't matter whether you have a degree or diploma—some basics are essential.
If someone wasn't built from the start, they'll become a very strange person later.
Some things, they won't be able to do at all.
That's when we'll say, "They finished their degree, but in practice, they are stubborn."
So, if you assess them early, all that knowledge will carry through well until they finish.

I: Thank you very much, number two. Welcome, number six.

R6: I want to add a little to what number two said—those statements from the beginning, "We're all nurses."
Old and new nurses are different.
Now it's like there's pressure—that "I'm here in class because I didn't qualify for medicine or pharmacy, or what I wanted wasn't possible."
So if we start with them from the beginning—I remember when I was studying—you had to explain why you were there, why you wanted to study nursing.
Others used to say, "I love the cap, the white uniform, how they inject patients."
So everyone had their own reason that pulled them into nursing.
But I don't know if now we should improve the first year—ask them, "What made you choose nursing?"
That will help you know what kind of nurses you have—maybe someone came as a shortcut saying, "I'm studying nursing, but I want to be a businessperson."
So as you walk with them from year one, two, and three, you'll know how to mold them.

By the time they exit, perhaps those with such mindsets will have already changed.
The one who didn't understand nursing well will now understand—and we get a better product.

I: Thank you very much, number six. Maybe since you're here, you understand the organization well as leaders.
What kind of nurses are missing in this organization? Welcome, number five.

R5: I think we have nurses—but what's missing is the **specialist level**—that expertise in a specific area.
For example, nowadays the life expectancy in Tanzania has increased.
So we would like to have people dealing with the elderly.
Along with their diseases, their behaviors are also changing.
A nurse caring for such people needs to be built with a different spirit—because it's not just nursing care—it requires **nursing tolerance**.
So I can say that skill is missing.
That's why sometimes we encounter challenges like being rude or uncooperative—either the patient was troublesome or the nurse responded poorly—because no one was prepared to handle that burden.
So I see that's what we're missing.

I: If I understood you well—**a geriatric nurse**?

R5: Yes.

I: Number four, maybe you have a contribution, welcome.

R4: Thank you. I would like to agree with the one who spoke before that nursing should also be for newborn babies. Sometimes you find yourself there just because the job placed you there. But the area that number five mentioned, there's a passion that you need to have when dealing with small children. Someone else may be placed there just because they were employed today and are told to be there, so they just go like that. But if we get people who willingly went to study that, when you send them to children, they go with heart, doing the work with peace and passion.

I: You mean a pediatric nurse, if I understood correctly?

R4: Yes.

R5: She's referring to the premature babies.

I: Maybe let me ask you since this is a government institution: what national issues would you recommend we include in the curriculum for the benefit of the country? Go ahead, number five.

R5: I know this might be outside the institution, but through our input, it might reach the top. In other countries similar to ours, they have systems—this nurse who is a fresh graduate from school, after training as a general practitioner, should go through postgraduate training.
For example, this nurse finishes a first degree and is attached to emergency to gain experience

for one year—they understand emergency settings. There should be one-year postgraduate training.
These postgraduate courses are different from Master's. In postgraduate, you are taught to work at a level higher than graduate. Then after that, you proceed to Master's where you write your thesis. But already, your work performance no longer has a question mark.
With our current curricula in the country... I finish undergraduate and go to Muhimbili, I work in emergency for a year, then go study a Master's in nephrology—in practice and reality, we see that in terms of decision-making, yes, it's fine.
But we want to produce nurses who, when they return with their superspecialized knowledge, the impact is clinical—not just to become a block manager.
Because how many block managers are there? How many heads of departments? Yet schools are creating curricula.
So we want to produce people who feel good doing their specialized work without question marks.
Currently, there's a loophole where a diploma nurse questions a graduate nurse, and the graduate nurse questions the Master's holder.
Now, in other countries, these questions are eliminated—you finish school, you're put in emergency, or ICU, work for a year, qualify, go to school—some courses don't require research or being given a pamphlet to explain yourself.
Instead, your exams are based on your competence in the specific area—say, critical care—and after that, you do a thesis in an area you've already excelled in practically.
If I come with my Master's and teach you, I say "That's not how it's done," I can put down my papers and show you the right way—while still having my education and research background in that field. I can still teach you.
If we get to that point as a country, we'll have helped ourselves.

I: Thank you. Welcome, number eight.

R8: Also, as a university, the demand for degrees has increased greatly. At Muhimbili, ENs (Enrolled Nurses) are no longer there—it starts from degree level. So the demand for degree-level training has gone way up. Also, affordability—the cost has become high, if you can help reduce it, that would be good. Thank you.

I: Let me ask, how do you see these degree nurses in terms of how prepared they are to carry out their professional responsibilities? And why do you see them that way? Go ahead, number five.

R5: Let me contribute. First, what we see is that degree nurses are greatly supported by **internship**.
Internship is what merges them and prepares them to at least get those competencies to be employable and do the job.
But if you take a graduate nurse who hasn't done internship yet, they are still a nurse with uncertainty—some things they can't be left to handle 100%.
Unfortunately, internship centers have increased—but for political reasons.
So those who get placements at Muhimbili, Bugando, KCMC, or Mbeya Zonal at least meet the demand I described.
But others are taken to Chato or Mtwara referral—which is also called Zonal—but in reality,

there's only OPD.
So this... going back to your question—it’s possible we continue seeing them as people who can’t be trusted to work or be hired as expected.
So the issue is: for your product to be maintained, right now there’s a strong relationship between where a student was trained and where they intern. So there’s that association.

I: Thank you, number five. Based on this discussion, let me ask, as we go to review the Hubert Kairuki curriculum, what key things would you like us to consider to improve? Number 5?

R5: Customer care, communication skills, IPC (infection prevention and control).

I: Anyone else?

All: *Silence*

I: As we near the end, let me ask—what are your suggestions for Master’s degree programs in Nursing and Midwifery to be introduced? Number four, welcome.

R4: Master’s in Psychiatry.

I: Thank you, number four. Welcome, number 8.

R8: Thank you. Another could be in **Theatre Management**, because in our country it doesn’t exist at all.

I: Why do you think we should start it?

R8: Because Tanzania doesn’t have a school offering that course. Maybe if you offer it at that level, we can access it.

R5: To add—right now, every ward has a health center, and every health center has been equipped with a theatre for maternal care.
That means there must be a nurse there.
And for that nurse to be there, they must have **theatre skills**—that’s the importance of producing such people.
But also, to add my own point—there is a big market and demand for Master’s in **Nursing Administration and Management**.
This is because most hospitals are run by nurses—24 hours.
For a hospital to run, the leaders are nurses—because they’re there 24/7.
There’s no hospital at any level where all staff leave.
But those who stay as supervisors are all nurses.
However, you find it’s hard to get people with the proper qualifications to make decisions in that area.

I: To continue that, in your work experience, what other skill gaps have you observed among specialist nurses, since you've started working with them? You mentioned administration. What else?

R5: Because I'm a leader, let me emphasize—someone finishes their Master's and is made Director of Nursing.
That's a huge responsibility, and maybe they didn't specialize in that area.
I went to study critical care—but you valued my general education and made me chief nurse.
That's already a gap.
When you say a leader should do this—you find most write things like "Monday I'm not available," "Tuesday I'm not available," while they are supposed to be there for people.
That's already a leadership gap.
So that's why I'm advocating for my point—others will talk from their areas—but I say we have a **leadership gap** in the nursing profession.
An institution can't run without leadership.
We're being forced to print leaders while we lack the skills—and this leads many into trouble—being fired or taken to tribunals.
Because results come back to haunt us—and we were given these responsibilities without knowing how to decide.
It's not just at the director or manager level—even the night supervisor should have space to learn.
You'll see those appointed as DNOs, RNOs, and heads of health centers—if they were certified that they can lead because of this package—along with their education, they also have this package.
So if you want to introduce Master's, I insist: there's a need to offer slots for people to study **Nursing Administration**.

I: Thank you very much. Now as we conclude our discussion—since we've reached the end—let me ask all of you: what is the most important thing to consider if we are going to introduce a Master's degree in Nursing or Midwifery? Number seven, welcome.

R7: The most important thing to consider first is **cost**—in terms of affordability.
The second is to set **entry criteria** that are aligned with other professions.
If it's a Master's in Nephrology offered at Kairuki—say a GPA of 4.0 or 3.0—make sure it matches other universities so we're not seen as using very low or too high standards.

I: Thank you, number seven. Number two, I saw you raised your hand.

R2: Another thing—look into **distance learning**. Don't focus too much on students being physically present.
No.
They can have two sessions per week and continue working normally.
This will reduce shortages.
Right now, degree holders are many, Master's holders are growing.
To prevent shortages, make it **distance learning**.

R5: To add on—I agree with her.
Distance learning, especially for Master's in Management—will make it accessible.
Most applicants will already be leaders—so making a **friendly learning environment** will attract them.
But another thing—to answer your question—it would be good if you **advertise your courses**.
Because this is Master's—not first degree—consider the applicant's **experience**.
If someone is applying for Nephrology, at least they should have worked in a medical ward.
If it's Neonatology, at least work experience in that area.
If it's Nursing Administration, at least they should have been **in-charge** somewhere.
If you set criteria like that—you'll get the **right applicants**, who have already built trust and a clear path to specialize in that field.

I: Anyone else would like to finish up?

All: *Silence*

I: I see no one. I would like to thank you all very much.
Really—I emphasize my thanks.
You are leaders with many responsibilities, but you gave us your time.
Truly, thank you very much.
We've reached the end of our discussion.

All: Thank you.

IDI Graduate #1

Interviewer (I): Good morning.

Respondent (R): I'm well, how about you?

I: I'm well. I'm from Kairuki—we're planning to review our curriculum, so we're collecting information to help us improve it: what should be added, what should be reduced. So as one of our key stakeholders who studied at Kairuki, we kindly ask for your cooperation in this discussion. I'll be asking you a few questions and you can respond as you understand. There's no right or wrong answer.

R: Okay.

I: Can you tell me in what ways the **Bachelor of Science in Nursing (BSN) program** has affected your work performance?

R: Affected?

I: Yes—has it affected you or helped you in any way?

R: The Bachelor of Nursing degree from Kairuki has had a **positive impact** on my life. It has helped me first to understand nursing in its full scope so I can provide care to people who are ill. It has also helped me personally—knowing how to **protect myself from diseases** and help others in the community avoid illness, and for those already sick, to provide **proper nursing care**.

I: Mh! What are your **future expectations**? And in what way has Kairuki's nursing program prepared you for the future?

R: My future expectation is to see the **nursing field grow** and become more **recognized** than it is today. Yes, people currently see it as important, but the **attitude is still not strong enough**. So I hope that, in the future, society will recognize that **nurses are a vital cadre** in people's lives.

I: Can you elaborate? You mentioned that it's not yet viewed as an essential cadre. How did people perceive it in the past, and how is it seen now?

R: In the past, people saw nursing as a **voluntary profession**. They viewed nurses as people who **lacked qualifications** and were just brought in to offer help. People believed that **nurses didn't love their work**, and so they didn't provide proper care. They also thought that nurses were people who **failed academically**, and that's why they opted for nursing. So, my perspective and hope is that, going forward, people will see that nursing is an **important and foundational profession**—because it offers critical support to individuals, and it also **creates employment** and **helps people earn an income**, thus overcoming unemployment.

I: What changes would you recommend for the BSN program at Kairuki? What should we add? What should we reduce?

R: Thank you. From my experience while studying there, I would recommend **increasing the time spent in clinical training**. Personally, I had **more theory than practice**.

I: So you mean more **clinical hours**?

R: Yes, the time for **clinical rotations** should be increased so that students get more hands-on experience—not just theory. I feel like I didn't learn enough until I did my **internship**, that's when I felt I truly learned. But while I was studying, I didn't get enough practical time. So I think that should be addressed in the next curriculum. Also, the **infrastructure**—when I was studying, there was a **shortage of buildings**, which created a lot of movement and interference. It didn't provide a **conducive learning environment**. One day you're in one classroom, the next day you have to shift and give way to others—you don't have a **permanent, suitable class**. That should also be improved, if possible, depending on **student intake**.

I: Okay, thank you. You said that time should be increased. So what challenges did you face **when looking for a job**? And what could have been done during your training to prepare you for those **employment challenges**?

Respondent (R): The challenge I encountered was that I had done **two interviews** while looking for a job, and I realized that when I was asked **clinically oriented questions**, I couldn't answer them well—but I was able to answer **theory questions**. As a result, my **first job** ended up being as a **teacher**, even though I had applied for a **Nursing Officer** position in a hospital. I wasn't successful, and I believe it's because I failed to answer ward-related questions. But when I went for an interview for a **teaching position**, I passed—because I had a good grasp of the theory.

Interviewer (I): Okay, so what should we do to improve clinical skills?

R: The **clinical hours** should be increased, and there should be **enough dedicated instructors**.

I: Thank you. How would you describe your **overall experience** during the BSN program?

R: My experience?

I: Yes, the experience you had.

R: Ah, I had a **good experience**. I studied at a college I'm proud of—Kairuki. The school provides opportunities to **interact with many people**. You learn a lot beyond your field. You also interact with people from **different countries**, so I feel like I studied at an **international institution** that teaches not just nursing but **life skills** too. Additionally, I appreciated that we learned some **non-core subjects** like **entrepreneurship** and **philosophy**. I think it was a great initiative by the school—many other students elsewhere didn't have the chance to learn these kinds of things. So I feel the school did well by adding content that's outside our core field but still **adds value both within and beyond the profession**.

I: Okay. How would you describe the **quality of facilitation**—both good and bad—in the BSN program?

R: Sorry, could you repeat that?

I: How would you describe the **facilitation**—what was good or what should be improved at Kairuki?

R: Things that should be improved include **infrastructure**, like I mentioned earlier—**more classrooms** are needed. And in the **curriculum**, there should be enough time for **field practicals**.

I: Okay, you've mentioned practicals already.

R: Yes, I've said that. Another thing...

I: Okay, let's move on. When you compare yourself to graduates from other schools, are there any areas where you feel they're ahead of you?

R: I don't feel they're ahead. In fact, I feel like **I'm ahead** in terms of **thinking ability**, or maybe we're at the same level—but I don't feel they've surpassed me.

I: Aha! In what way do you feel you're ahead?

R: It's because I **understand nursing**, but also **life beyond nursing**. I've gained knowledge in **other life aspects** as well. At Kairuki, there's a strong emphasis on **discipline**. We were taught to be disciplined, to dress properly, and to love what we study—not just as a source of income, but as a **passion**. The teachers inspired us to **love what we were learning** and to do it from the heart—not just because it's your job, but because it's your **calling**.

I: Okay. What **skills or content** from Kairuki have helped you in your work?

R: The biggest thing that helped me at work is **discipline**. I feel like I was molded to be disciplined. While studying at Kairuki, even though I was far from my parents, the **teachers followed up** on us—asking why you didn't attend class, why you missed fieldwork. If you didn't meet a certain attendance percentage, you were asked to **repeat your field placement**. That made me more **systematic and focused**, unlike if they had left us to be careless.

I: On the flip side, what **courses or content** did you learn that **haven't helped you**—or didn't seem relevant to your job?

R: Honestly, none. **Everything I learned was useful.**

I: Everything was useful?

R: Yes.

I: Okay. How would you describe your experience working **beyond your scope of practice**?

R: My experience working beyond my scope?

I: Or what challenges have you encountered?

R: The challenge I mentioned earlier. While I was an intern, I **felt like I couldn't fully practice**. Not that I was completely incapable—I could perform to a certain extent—but **not to the full level**. That's the main challenge I can explain.

I: Okay. Based on our discussion, you've explained your challenges and where the gaps are. What should we **focus on** when reviewing our curriculum?

R: I think we need **more clinical instructors**, especially for the clinical sessions. Clinical training should be given the **same weight as theory**. There should be enough instructors to **help students learn practical skills**. And if possible, there should also be **transport** provided for students. For example, we used to go to clinicals at **Mwananyamala** and **Muhimbili**, but there was **no vehicle** to take us. That was a challenge. If possible, the school should consider getting a vehicle. I saw that the medical students had one—but I never saw one for nursing students. If possible, this should be considered to help students **arrive on time** for clinical rotations and **get full exposure**. Also, there should be **enough clinical instructors**—not just one for the entire BSN class. There should be specific instructors for **surgical**, **medical**, **pediatric**, etc. That really helps students **learn practical skills**.

Interviewer (I): Do you think that the **lack of clinical instructors** or **lack of transport** affected your ability to gain the **competence** you wanted?

Respondent (R): Yes, it did affect me.

I: To what extent?

R: Because I didn't have a **dedicated person** who was with me full-time while I was in the ward. Yes, the nurses were there, but they were **busy fulfilling their own duties**—so it wasn't easy for someone to fully support me the way I expected. If there were a **dedicated clinical instructor** who was with me **full-time**, throughout all sessions, moving with me **from department to department**, I believe that would help a lot more.

I: Thank you. What's your opinion regarding the **introduction of a Master's program** at Kairuki?

R: I think it's a good idea. Even when I was studying, there were **MMed students**, and I had the chance to interact with them in the field. I saw them doing well. So it's a good idea to introduce **Master's programs in nursing—as long as there are enough instructors and proper facilities**. That would be great.

I: Which **Master's programs or specializations** would you like to see introduced to match the **job market**?

R: I think **Midwifery** and **Critical Care**. I'm not sure if it's been approved in Tanzania, but something like **Nurse Practitioners**—like they have in the UK—would be really helpful. Nurses

have already started **prescribing medication** for conditions like HIV under some programs, so if such roles are supported by law, it would be a good direction to take.

I: Okay. In your area of specialization, you've focused on…

R: MPH (Master of Public Health).

I: In that MPH you studied, were there any **missing or insufficient courses** that you think should be added?

R: In the MPH I did, all the courses were related to health, but there were **no additional subjects outside of health**. For example, topics I studied at the undergraduate level weren't included. You only learn about **community health**, and that's it. So if other **value-adding topics** (not strictly health-related) were included, that would be very helpful.

I: Okay. Where do you see **skill gaps** in the roles of **specialist nurses**, considering current and future work demands?

R: The gap may be with the **individual themselves**—a **lack of commitment**. I think that's an issue among nurses. Also, **not having the right attitude** toward one's own profession, and **not being committed** to what one is doing. That could be a major gap.

I: Mh.

R: For some—not all.

I: Not all.

R: Mh [yes].

I: What's your take on the issue of **communication**?

R: Communication...

I: Yes, between **specialist nurses and patients**. Sometimes there seem to be issues—what's your view?

R: I think we do communicate—but **how** we communicate matters. For example, the **language** used—someone might use language that's **not appropriate** for a sick person. That's a challenge too, especially in a **care profession**.

I: Okay. As we approach the end of our discussion, what is the **most important thing** we should consider when launching a **Master's in Midwifery**, which we're planning to introduce?

R: Mh! Give students **enough time** to **interact with patients**, and ensure there are **enough instructors**. They should have sufficient time to **work directly with patients**, because **at the end**

of the day, if you have the theory but don’t know how to practice, I don’t think the patient will be helped as expected.

I: Mh! Thank you so much. This discussion has been very insightful. The ideas you’ve shared will be very useful in **improving our curriculum**. On behalf of Kairuki, we wish you all the best in your work.

R: Thank you.

I: You’re always welcome back to Kairuki.

R: Thank you.

I: Alright.

IDI Graduate #2

Interviewer (I): Welcome to our discussion. As I mentioned, you're a graduate from Kairuki. Let me begin by asking, how has this **Bachelor of Science in Nursing** from this institution influenced your work performance?

Respondent (R): For me, it's had a significant influence. First of all, the education I received was a bit **advanced**. Even when you talk about nursing, it felt somewhat advanced. Although when we went to the ward, we encountered things that were more **routine**, in class we were learning more **advanced content**, and that encouraged me a lot.

I: You're saying what you had was advanced knowledge?

R: Even when I went to the ward, I had the **desire to perform** things in an advanced way—even though the **real-world environment** in hospitals was different. I wanted to do things the **standard way**, and distinguish myself from someone with a diploma or certificate—I had more knowledge. I didn't want to feel left behind compared to other professions that seem to be in the lead. For example, during doctors' rounds—doctors have the mandate to prescribe—I wanted to be **involved in those rounds**. I don't feel totally new to it, I just lack the "go-ahead" to participate. But when I'm in the rounds, I feel **oriented**.

I: Meaning, you feel like you're part of everything in that process?

R: Yes, because during rounds, they start by discussing the patient, cracking the case, taking history, diagnosing, then implementing interventions. These are things I didn't lead, but when they were discussed, I was not a stranger to them. For example, if they talked about **cervical cancer**, its signs and management—I wasn't totally new. There are things I'm told to do, and I'll think, "Hmm, no—this shouldn't be done like this."

I: But we also say **challenges are part of life**. Have you faced any challenges in your work? And if so, how did your nursing degree help you handle them?

R: There are many challenges…

I: Such as?

R: First, when you talk about the **nursing degree**, it represents maybe **30%** of those in the workforce—others have diplomas or certificates. Our education is a bit more **advanced**, but you're surrounded by people whose education is not to that level. The first challenge is **bias**.

I: In what way is bias a challenge?

R: When you work with someone who thinks, "We're all doing the same routine—how can you tell me you have a bachelor's degree? What more do you bring to the table?" That gives me strength to say, "Okay, we're doing the same thing, but I'm doing it in a more **advanced way**." Our degree goes **much deeper**, including **leadership, communication skills**—it's not just about

nursing. You find that others, especially diploma holders, were trained for **task-based roles**—not deeper subjects like **psychology**, **communication**, or **leadership**. So, even your **behavior** becomes different. You're able to **read the client** and know how best to provide care. Sometimes, someone else just follows basic instructions: "I was told to do this in the ward." But for me, maybe I start with **psychological therapy** before going into medical care. So there's a **difference in approach**.

I: Because of your bachelor's degree?

R: Yes. So even when there's debate like "we're all the same," I know through my **work performance** that, no—there's a difference.

I: Let me ask: What are your **future expectations**, and how has Kairuki's nursing degree prepared you for your professional advancement?

R: First of all, I'm someone who doesn't want to stay where I am—I want to **move forward**.

I: Okay, what are you hoping for in the future, or what plans do you have?

R: In my expectations, I may not want to go too far, but I love **health-related fields**, and nursing has **exposed me to many areas**: community health, research, teaching, clinical work. It has given me a **lot of choices**, and I can now **choose my path**. I'm still exploring whether I want to focus on **community work**, **clinical care**, or something else—it hasn't limited me to one direction.

I: So it's given you a **foundation**, if I understand you correctly?

R: Exactly—it's like it opened a room full of chairs and told me, "Choose where you want to sit." It hasn't boxed me into one option.

I: What **changes** would you suggest for the BSN program at **HKMU**?

R: At Kairuki?

I: Yes.

R: I think many things are going well—let me start by **praising that**. As I said, it's **advanced** in how things are taught. But what I think should be improved is the **pressure**…

I: You mean during the **curriculum review**, there are things you'd like us to consider?

R: Yes. There are topics we should learn, but **not put too much pressure** on. Some subjects are helpful, but others are more "good to know" rather than essential. They shouldn't be overemphasized.

I: What should we focus on in this **curriculum review**?

R: I think we should focus on **future-oriented topics**. For example, **research**. It's a very important area, and it's not something that will ever become obsolete. We should really invest in making students **fully understand research**—not just rush through it. Another topic I was personally **very interested in** was **entrepreneurship**. I felt like we were **thinking outside the box**. As for **core nursing content**, I think things are going well, but we shouldn't be **too theoretical**. We need to **emphasize practice**. When assessing students, don't just rely on theory—look at **real-world work environments**, then evaluate what needs improvement. For example, in anatomy class, nerves are perfectly illustrated in books—but in real-life cases, things may appear different. So we teach students based on books, but we also need to consider **practical realities**.

I: What challenges did you face when **looking for a job**, and what could have been done during your training to prepare you for the **job market**?

R: The job market—(laughter). I think the issue is **awareness**—that's something we need to understand.

I: In what way—awareness?

R: Being aware that **jobs are available**, but you'll **struggle**. When it comes to employment…

I: Sorry, what do you think should have been done during your training to **prepare you for the job market**?

R: I think…

I: Let's go back to what you said—**awareness**.

R: Awareness about the job market in **Tanzania**—people see you as **advanced**, and that **intimidates employers**. They think, "If I hire this person, they'll demand too much." So if a graduate walks in knowing "I'm valuable, I come with a price," but also understands how the **market perceives** them—that helps. Some people just want a job. They say, "Even if I'm a bachelor's graduate, just pay me like a diploma holder." But that causes problems. Another thing is that **real work is more serious than classwork**…

I: In what way?

Interviewer (I): When you're in class, you're thinking, "I'm just going for rotation as a student and then coming back." But when you get into actual work, people look at you like, *"You're doing our job, and we expect you to know what you're doing and to meet standards—we're giving you the mandate."* For example, in some places, it feels like you **own the whole environment**. They don't care if you're straight from school—you're the one arranging **5S** systems, organizing everything.

So if you have a mentality of "someone will tell me what to do," even if you **know what to do**, but still expect a push, that becomes a problem. People need to be aware that **in the workplace, it's about you**—you're no longer a student. You're treated as someone who knows everything. When I enter the ward, that **awareness needs to be present**.

Even as interns, we still view ourselves as students, but a time comes when we'll take over and lead that place. Sometimes I'll be the one suggesting interventions, pointing out challenges, and **correcting others**. But if I keep viewing myself as "just a student," then it's a lack of awareness.

I: So if I understand correctly, that **awareness should be built during training**?

R: Yes—**among students themselves**.

I: The key is making students aware that we're not just teaching them, we're **building them to lead and initiate change**—not just to be directed.

I: Now, how would you describe the **experience you gained during your BSN program**? What did you like about it, and what needs improvement?

R: What I liked first was the **academic depth**. In the BSN program, some subjects, like **gynecology**, really showed the value of schooling—like **anatomy**. It wasn't about becoming a "routine nurse," but someone who reasons things out. Even if I wasn't doing something directly, I understood the **theory behind it**. That was the first thing I liked.

Second, I liked the **rotations**. What wore me out, though, was that the place was **so busy** [laughter]. By 5 AM, everything was chaotic—I wish the schedule was a bit more relaxed.

I: What would you like us to improve there?

R: I think it shouldn't be about spoon-feeding students every single thing. Sometimes, give them space to **discover for themselves**. I don't know how best to phrase this, but if you **overload them**, they get confused. [laughter] So some topics could be introduced with more **flexibility**.

I: Like how?

R: It also depends on the teacher. Some can make a subject **easy**, and others can make it **difficult**. There are times when a teacher could say, "Try to research a few things on this topic yourself," then come to class and discuss it.

But if a teacher brings 100-slide PowerPoints for surgical topics, and another 100 for something else, and exams are around the corner—**that's overwhelming**. A more **summarized and focused approach** would help.

I: So simplify the content to make it more digestible?

R: Yes. And simplifying **doesn't mean** removing the core points. Some things students will grasp themselves. Some teachers come with just one piece of paper, the class discusses, and **key insights** come out. That's very effective compared to listing every detail on the board—it can be overwhelming. [laughter]

I: Teachers are facilitators. So how would you define a **good facilitator or teacher** in your BSN program?

R: A good facilitator is someone who **doesn't assume you know nothing**, but instead encourages you to **speak in detail**. For example, if the topic is **pain**, ask: "What do you know about pain?" Let students share, and then the teacher adds key points like "types of pain," etc. This approach makes the information **more memorable**.

Some teachers come in with huge explanations, and students start dozing off. [laughter] A **good facilitator simplifies difficult content** and even makes you like the subject. For instance, exam questions should be **motivating**—don't test something so minor that even though students studied, they still get stuck.

Let's say the topic is **pain**—design the question so that **if a student opened the slide**, they can at least answer something. Don't let them walk away empty-handed.

I: So, focus on **key concepts**?

R: Yes. I remember a teacher I really liked—if you reviewed the slide at all, you'd definitely get something right. Because usually, the problem isn't that a student knows **nothing**—they might just miss **one key point** among a hundred, and that causes unnecessary confusion.

I: You studied at Kairuki. In your job, how do you compare yourself to other BSN graduates from other universities? What stands out for you?

R: I feel **smarter** than others…

I: In what way?

R: Smarter in **decision-making**, and I'm smart enough to **ask** when I don't know something. If I don't understand something, I'll go back and **research** or **clarify** it.

For example, once I encountered someone who gave a patient an **IV fluid to drink**—saying it works just as well. I thought, "Even if you don't know something, there's a **way to seek clarity**."

Also, I've noticed that **Kairuki graduates are smart enough to stand their ground**. If they know something is **unprofessional**, they won't just go along with it because someone said so.

I: In your current job, what **skills from your training** do you find most helpful?

R: [Laughter] I use **a lot of what I learned**, especially from my **internship**. Clinical management is my strength—I'm good at **prescribing** and overall **patient care**.

Beyond that, **leadership** and **communication skills** are areas where I also feel strong. In my current role, I often deal with **clients who need psychological support**—it's not exactly psychiatry, but I find it **easier to manage such clients** than others do.

Some people struggle, but for me, it's very manageable—I've never felt like I couldn't handle a psychiatric or any other complex client.

I: In the BSN program, what **course or content** have you **not used at all** in your current job?

R: For me, probably **anatomy**—right now, it's **not needed** in my current role.

I: Is there a course that you've **never applied** since you started working—something you studied but **never used**?

R: [Laughter] There are quite a few.

I: Which course stands out as the **least relevant** to your current role?

R: In my current job, there are many, honestly—mostly because I'm **not doing clinical work**. I don't use **surgical** or **medical** nursing much anymore.

I: What are you focusing on now?

R: Right now, I'm mostly involved in **epidemiology**, **informatics**, **community health**, and a bit of **research**. It's a mix—I might go out for data collection, or do something related to **quality improvement**. So I use **bits of everything**, depending on the task. But because I've focused more on the **community side**, I don't use **clinical skills** as much anymore. If I were working in a ward, medical and surgical skills would be important. So it all depends on the **role** you choose.

I: Now, based on our discussion, what would you like us to consider when we begin reviewing the curriculum for the Bachelor of Science in Nursing program? What should we focus on?

R: The most important things...

I: As we begin reviewing this curriculum?

R: If I speak generally, I would say let's focus on things that are important.

I: Like what?

R: But here I face a challenge because **everything is important** in its own way. For example, I might say let's focus heavily on **medical and surgical nursing**, but then there's **research** and **epidemiology**, which for someone who wants to specialize in that area, will be very important. I believe **each subject has key elements that are essential to know**. If I were to say something like **informatics** is not important or **biostatistics**, which we studied in second year, feels a bit distant, I'd still be cautious. Every course has relevance, but within it, there are **core elements** and some that are **additional**. If we were to remove entire subjects, we'd end up **limiting opportunities**. The beauty of nursing is that it opens you up to **multiple pathways**. If I say epidemiology isn't important, then someone who wants to specialize in it will be disadvantaged. Similarly, for someone interested in **critical care**, emergency training becomes crucial.

I: Now, what are your thoughts on establishing **master's-level nursing and midwifery specialization programs** that align with the current job market?

R: Regarding the job market?

I: Yes.

R: For master's programs, I believe we should **focus on areas with real potential**. Some programs exist, but their **market value is low**. Let me give an example. I once saw a nurse with a master's degree working on a **general ward**. That degree could help with leadership or promotion, but **it didn't differentiate** them significantly from someone with just a bachelor's degree. So, when developing master's programs, we need to **ask if there is a true need** for the specialization, and whether it gives someone **added value**.

I: So, what kinds of master's programs do you think match the job market?

R: Right now?

I: Yes.

R: I see **nursing in critical care** as having strong demand. Then there's **cardiology**, **neurology**... **pediatrics**, not so much—unless someone plans to work abroad. But I see many people diverting into **public health**, **case management**, and other areas that aren't necessarily **nursing-focused** anymore.

I: In your work setting, what kind of master's program would have helped you most, and why?

R: In my current role, I would love to see more emphasis on **computer skills**—especially for **Microsoft Excel**. I really wish I had more hands-on exposure to it.

I: Why computer skills?

R: Because I use them **almost every day** where I work. Of course, other workplaces might need different things, but for me, I wish I had learned **computer proficiency** more deeply.

I: When you say computer skills, you mean?

R: We use computers to prepare slides and documents—things like **Microsoft Excel** and even **SPSS** for research. These skills make someone **multi-functional**.

I: Where do you see **gaps in skills** among specialized nurses and midwives—those with master's degrees—considering the current and future job demands?

R: You mean in practice or in knowledge?

I: Yes—skills gaps in their professional duties, now and in the future.

R: One gap I've seen is that nurses return from master's programs **with knowledge**, but the **challenge is in mandate and recognition**. A nurse with a master's degree may enter the ward, but an MD who has completed a five-year program is still more recognized. That **lack of recognition** affects motivation. The more you practice, the more **your standard should grow**. If someone has studied **gynecology** deeply, they should be allowed to work on **advanced interventions**—under clearly defined conditions. Because the **goal of advanced education** is to solve problems that cannot be solved with a lower qualification.

I: Sorry—when you mention "conditions," what do you mean?

R: I mean the **work setup** or **systems**. Even a master's or BSN holder is still **just viewed as a nurse**. Even though I've studied deeply and can perform tasks equivalent to a doctor's, I don't have that **freedom**. A nurse with a master's in gynecology is still just called "nurse" in the ward. But I expected they'd be **handling critical cases**—just like a specialist is expected to. That's why in **community health** or **public health**, there's no difference between me and a doctor—we do the same work. And now that I'm doing my master's, we're on **equal footing** in many ways.

I: As we wrap up, what is the **most important thing** we should consider as we develop a **master's-level nursing and midwifery curriculum**?

R: When you're creating a new curriculum...

I: Yes—what should we consider as we begin these programs?

R: My point is still the same: the **training should be advanced** so that when someone graduates, you know clearly:

I: By *"training"* do you mean that the **competencies should clearly show the difference** between one graduate and another?

R: Exactly. We should be able to tell what makes one person **capable of doing more** than the other. Even if two people study similar content, one goes **deeper**, and the difference should be **visible**. Someone should be able to say, "I'm the cream," and **demonstrate** it—not just say it.

I: Thank you very much. We've reached the end.

IDI Graduate #3

Here is the **full English translation** of the provided Swahili transcript:

Interviewer: Okay, as we mentioned earlier, we are reviewing the BSc.N curriculum and as part of the review, we are collecting feedback from various stakeholders to help guide the process. As a graduate, you are one of the key stakeholders we want input from. So there are a few questions we will ask each other and by the end, you will have helped us a lot.

Respondent: Mmmh.

Interviewer: Okay, so as a graduate, in what ways has this Bachelor of Science in Nursing program affected your job performance? What challenges are you facing in your current job?

Respondent: Do you mean challenges I face because I'm a nurse or just general work-related challenges?

Interviewer: Aah, any work-related challenges in your current nursing role.

Respondent: The question is too broad, madam.

Interviewer: Okay, you are a graduate of a bachelor's degree, right?

Respondent: Mmmh.

Interviewer: So we expect that we gave you knowledge and skills to do your work, right?

Respondent: Mmmh.

Interviewer: So when you compare the knowledge and skills we gave you with what you're currently doing, what challenges do you face as a nurse?

Respondent: Okay, maybe the main challenge I face is because I work in an institution under the local government—TAMISEMI. The big issue here is that when you try to apply standards—doing things the way you were taught—you find it difficult because of existing practices. For example, at this hospital, most nurses are medical attendants, yet it's a Designated District Hospital (DDS), previously run by a mission but now under government management. They used to get many donors, but now they don't, so there's a lack of resources, which makes it hard to do the job as required.

Interviewer: Mmmh.

Respondent: The challenge is that people don't strictly follow professionalism. So when you try to do things correctly, you stand out as someone who's trying too hard, and that creates friction.

Even if you're right, others see you as different, like you're trying to show off. How will you capture that in your questionnaire?

Interviewer: Okay, from what I understand, when you try to apply your classroom knowledge in the field, you struggle due to environmental factors like lack of equipment.

Respondent: Yes, mmmh.

Interviewer: What else hinders you from practicing as expected?

Respondent: Okay maybe the biggest challenge that I face, because I am in a... I am in an institution that is under local government—TAMISEMI. Yes, the big challenge is that here, I mean if you… if you want to provide—aaahhh—if you want to do things according to the standard, that what I studied is what you want to practice, you find you cannot do so because... there are those who call themselves senior staff... there is like… there is… there is some kind of zone of routine, where they do work in their own design. It's not very different, but somehow it's not the way it is supposed to be done.

It might be because… aaaahhh for example here, like here in the hospital, most of the workers are medical attendants on the nursing side—medical attendants. But this is a DDS (Designated District Hospital). So it is a mission hospital but also the government has its stake in it. So in the past they used to get a lot of donors, but now it seems there are none, so many supplies you find are not available. So even doing the job becomes a challenge because availability of equipment is not as per what is needed.

Interviewer: Mmmnhhh.

Respondent: The main challenge is that people do the work following professionalism, but... even that is to a limited extent. So when you want to do things the way they're supposed to be done, you find yourself different from others. Mmmhh... you yourself understand, when you are doing something—even if it's correct—but others are not doing the right thing, you'll appear like someone very different, who maybe looks like they want to work too much, and stuff like that. I don't know how you will put that point in your questionnaire over there.

Interviewer: Okay, so from what I understand, is that when you want to apply the knowledge that you got, as it was taught in school, you face barriers due to environmental reasons—for example, lack of supplies?

Respondent: Yes, mmmnhhh.

Interviewer: Okay. And what other things do you think you've encountered there that prevent you from practicing as you had expected to as a bachelor's degree nurse?

Respondent: Aaahhh, apart from that I see mostly the big reason is that one. Eeehhhh. Because… other administrative issues don't have a problem.

Interviewer: Okay, and in what way has your bachelor's degree in nursing helped you to deal with various challenges in your work area? That is, as a BSc.N graduate, how have the knowledge and skills you acquired helped you to cope with challenges in your workplace?

Respondent: Okay, it helps me to a large extent because there are some challenges that when they arise, because I have... I have... I have skills, because I have knowledge as a BSc.N graduate, I find myself... I apply them. For example, there are times—there are times you may find that maybe there are... there are... there are conflicts. Me, here in the hospital, I am in the administrative rank—after the patron and matron, I am the next in line. So you find that there are many things that—because I studied them in class, maybe things like solving problems, problem solving—so you find it helps me to deal with things of that nature. For example, if there is a shortage, it's a challenge that arises at work—before it reaches the relevant authorities like the patron or matron, you find I address it. So largely, the skills... the knowledge and education of my nursing degree helps me to deal with such challenges.

Interviewer: Okay, so it has... it has given you problem-solving skills, which you use a lot there. What other skills have you gotten that you feel you use a lot?

Respondent: Aaahhhh, I mean, if you say skill... mentioning one skill after another, there are many. For example...

Interviewer: At least mention a few.

Respondent: Sorry?

Interviewer: At least a few, even if you mention two or three.

Respondent: For example, there are times—if I'm not too busy with administrative issues—I sit in the minor theater. You just go and find, like these... these small procedures: incision and drainage we do; if there are people who come with cut wounds, we do stitching; if people come with fractures, we do POP application, we do. So there are many things we do based on what we learned—what we learned, yes.

Interviewer: Okay. Now, what are your future expectations or what are your plans?

Respondent: In relation... in relation to the license or? In my career?

Interviewer: Eeehhh.

Respondent: Aaahhh, you know these days—these days, nowadays—education is... I can say it's a business, like any other business. Although we aim to provide service, but there is competition. Eeehh, there is competition. In the past, there were few bachelor's holders, so the demand was high for the services they offered. But now we are heading toward a generation where we will have many bachelors, so it means the demand will be normal because the supply is high. So my expectation, I mean I will... I will... I expect to go study further in order to expand the scope—scope, scope in this career of mine. Yah.

Interviewer: Okay, so you aim to further your education?

Respondent: Yes.

Interviewer: And now that you aim to further your education, you are already a bachelor's graduate—do you think this bachelor's degree has prepared you to advance further academically?

Respondent: I mean this has built me a lot, because when I look at my nursing degree—first of all, it is conne... it's not limited. It is connected to many things that if I want to advance academically, I can attach myself anywhere I want to attach. Yes. So it hasn't limited me like now you've studied nursing so you'll... I mean you're just limited to that same field. I can do things that are... are not even related to... to nursing but which are health-related. So I think it has... it has... it has... it has opened up options for me to... to advance academically—not just in nursing things but even in other things that are non-health related.

Interviewer: Okay, for example, you—in those non-health related things—what are things that, if you had the chance, you'd like to advance in?

Respondent: Aaahhhhh, actually I think... for example, things like epidemiology. Epidemiology is not directly associated with nursing, although even in nursing, aaahh… eeehhh… aaammm… you can use the concept of epidemiology, but even other areas need epidemiology. Yah. So thinking of things like epidemiology, I was thinking about... another thing that is related to nursing—for example, critical care and trauma, or mmhh… So for example, if I say I go take a master's, it's either I'd go for MPH or take something in epidemiology. Mmhhh.

Interviewer: Okay, now you as a stakeholder in this program—you studied the bachelor's degree and the whole program from first year to fourth year—if you were told, what changes would you like to be made to this bachelor's program? What changes would you propose to be made?

Respondent: Aaahhh, to this bachelor's program?

Interviewer: Mmmh.

Respondent: The first thing—unlike other bachelor's degrees—this bachelor of ours is... is a bachelor that at a glance, you cannot differentiate it from its... lower levels, which are diploma and certificate, in terms of job performance, if you don't look closely. So what I would want... what I would propose to be done—and I think this should come from the ministry—is to create **modalities** that define responsibilities which are directly specified for Nursing Officer I, who is the bachelor's holder. These would help differentiate him/her from those others below, just like how it is for COs and MDs. There's a clear boundary: this one is a CO—there's something he cannot do but an MD can. That helps even in building... aaahhh... building the prestige of the title you hold. Yah. But also, another thing... aaahhhmmm... when we are in teaching—teaching I mean while we are at school—we were taught... we were taught a lot of things, many things that when we come into the workplace, those things we use them... sometimes we just use them as advice but not to... yaani... to directly apply them ourselves. So I was thinking—if really we are taught and understand well and we do know these things—the curriculum should be made in a way that what we studied isn't

just for giving advice to a doctor who already knows what they studied, but I should have **access to... to... to...** I mean, I should have **power and mandate** to apply it, since I studied it and understood it—that's why I was even given a license. Yes. For example, let's take one of the courses—medical nursing, okay? For example, medical nursing is typical internal medicine. Eeehh—it's internal medicine. If you study, let's say... paediatrics, the paediatrics we study isn't that different—it's not... it's not much different from the paediatrics studied by MDs, although there are a few things where they go deeper due to their pathway. But even what we study at bachelor level—if you look at our curriculum—it's very few things that differ. For example, if I study malaria, the malaria I study and what they study is the same. But in the end, I will be like... I'll just be looking, for example, if this patient has severe malaria, I'll ask: has this doctor prescribed artesunate? Of which, if he's studied, of course he will prescribe artesunate. So they should create a modality where the things we studied—we have the power to apply them directly, not just advise. Because by not doing that, sometimes you may find you lose skills just because you don't utilize them.

Interviewer: So if I understood correctly, if you were trained on how to give artesunate, then when you get there and have a patient, you should be able to give that artesunate—not just suggest that a doctor could give it, right?

Respondent: Eeeennnheeeee, yes. Because we are... we're treated just like assistants—but you know, nursing is an **independent profession**. I am a professional.

Interviewer: Okay, and what challenges have you encountered when looking for a job? And what do you think could have been done during your training to prepare you better to face the job market?

Respondent: Aaahhhh, for now... for now, the job market has one major challenge. I think if they were using... if they were using a system—I mean a system that **auto-selects randomly**, I think it would be good. But practically, I don't think that's how it works. Eeehh... the system of... of... of job acquisition looks fine on paper, but I think those who **operate** the system—that's where the challenge lies.

Because what happens is, it's very few people whom, if you try to ask them "How did you get your job?"—you'll hear: "Well, I was connected by so-and-so, they told me to send a letter, do this and that, and then later I was called for an interview—I did the interview and I got the job."

So now, most jobs are **acquired through that modality.** Now, there's this person who doesn't have anyone up there—has no one to **link him or her** to someone else who has access to placing them in a certain job. That kind of person will study, but in the end, they will **struggle a lot** to get a job. Because the **modality** of getting a job we've found ourselves in—even though it's not the proper procedure—**doesn't favor** that person.

Because if you don't have **connections**, you are more likely to **miss out on a job**, although that doesn't mean you can't get one—but you're more likely **not to get it**. So the challenge... the biggest challenge—I don't know which polite word to use that won't sound too harsh—but that's the main challenge: **you need to have someone you know**, who can help connect you to someone who will help you get into a certain position. That's the challenge. Although... qualifications matter, but in

the end you can have all your qualifications, but if you don’t have someone to **link you**, to **connect you** to someone who might be in a position to place you somewhere, you’re more likely **not to get** that position. That’s the biggest challenge right now.

Interviewer: Okay, so did you personally encounter that challenge? Or you didn’t face it?

Respondent: Aaahhhh, I can say I **did not encounter** it. I can say I didn’t encounter it because the job I got wasn’t... I mean, it’s a government job through TAMISEMI, but we didn’t apply in the usual way—like, you go online, apply, then names come out and you find your name on the list. **No.** It was those types of jobs where some institutions request to hire people—government gives them access, saying "Go find people to employ, we’ll be the ones paying their salaries." So I applied in two places: one was a **mission hospital in Kigoma**, and the other one is where I am now. I ended up being selected for both. So that’s why I say I didn’t face that challenge—mmmmm—but many people say they have, and that’s the **reality**.

Interviewer: Okay, so for example if you were told... is there something that could have been done maybe during your training to prepare you better for facing the job market? What would you say should have been done during your BSc.N program? After getting employed and hearing about the challenges others face, what do you think could be done during undergraduate training to better prepare a graduate to compete in the job market?

Respondent: Okay, the thing that could have been done... I think it’s being done, but with just a **little input**. Because at the end of the day, to get a job—apart from these government ones where there’s no interview—**for most**, you have to do an interview. You **must** do an interview. Eeeehhh... I think the part about **how to do interviews** should be emphasized. Because many may be missing opportunities simply because they **don’t know** what things to consider maybe during interviews, or maybe during the **application**—what things should be included in your **CV**? You might have your **skills in place**, but the **way you present them** to the person offering the job may cause them to perceive something different—just because you didn’t know how to **present yourself**. So I think that **aspect should be added**, to help people **enter the job market**. Because I believe if someone has studied and graduated, they already have the **knowledge, skills, qualifications**—the only thing they might not know, or may not have, is how to **present themselves**, how to **sell themselves** to the person who has that job or that opportunity. Yeah.

Interviewer: Okay, so how can you describe your experience—based on the experience you got during your Bachelor of Nursing training at Kairuki—what are the things that you liked or were good in the program, and what are the things you feel need improvement? Let’s maybe start with the things you felt were good during your training in the BSc. Nursing program here at Kairuki. What did you see as good in the program from first year to final year?

Respondent: Aaahhh… the things I saw as good… aaahhhh… I think the **sequence**—the sequence of courses from… when a person starts from first year up to… up to… up to completion—meaning the **flow of the courses** as it’s structured. So you find yourself… you find yourself studying a certain thing which, when you finish and go to the next level, **what you studied before helps** with what comes next. So at the end of the day, of course yes… yeah, the **sequence of courses**—how it’s arranged—it doesn’t confuse you like… you’re in first year but you’re being taught something

that you were supposed to learn in third year. Another thing is the **seriousness** and **commitment** of the institution—how, I think, it has built a kind of **culture**, eehh… there's a level of seriousness such that even if a student is not serious, **you are more likely not to graduate**. Because I see the institution is committed to producing **products that it believes** will do well out there. Eeeehhheee.

Interviewer: Okay, and what are the things that you feel need improvement?

Respondent: Aaahhh… the things that need improvement… heeeee (laughs), Kairuki… **the fees**, the fees are… are… I think to a large extent they are… I don't know what **criteria** were used to set that tuition rank, but nowadays, in the world we live in, **everyone has a right to education**—whether they're from a **low class** or **high class**. So if we aim to provide education to **all kinds of people**, I think the **amount of fees charged by the institution is high**, and it targets a certain economic rank. This child, this poor child who might also have wanted to study—**many struggle a lot** to study, and it might be **one of the reasons** why some people who come out of there don't do well—because the person studies under **stress**. So I think that matter should be looked into. It's a **good institution**, but in terms of fees, to a large extent, it's… it's… I wouldn't say it **oppresses** students—but I don't know if that would be too strong a word—but **students really struggle** because of the high tuition fees that the college has set, eeehnhee.

Interviewer: Okay, apart from fees, is there anything else you feel needs improvement?

Respondent: Another thing that needs improvement… aaahhhh I don't know where the problem lies… what I know is that even now it's still ongoing… there's this **issue of fieldwork**. Field, field, field, field works… Aaahhh… when we went for field placement, **students were not given anything**—no one followed up to ensure that people got **allowance for field**. And considering the high tuition fees and that you're going into a **new environment**, if there's no mechanism to support the student to reach there, you end up doing your fieldwork **under very hard conditions**. So I think… I don't know if that's the **finance office** or who's responsible, but that issue should be looked into. Yah… **field**, yes. Also, another thing—**the timing** of starting **clinical rotations**. For nurses, they start entering properly in **third year**, even though in second year they have PNU, it's **very little** for them. But because we need to be good in both **theory and practice**, they should try and… I don't know how… because the timetable itself is tight, there's a lot to cover—but they should try to **start clinical rotations earlier**. For example, in other universities, rotations start from **first year**—they study a little then go into the ward. For instance, if I look at Bugando… I think Bugando, even Muhimbili—from first year they start entering. So even us, the things done elsewhere, we can adapt—**for the sake of improving the quality of training we give to our students**.

Interviewer: You were taught by different facilitators. What makes a good or bad facilitator in your opinion?

Respondent: A good facilitator is approachable, available, and willing to explain until you understand. Most of our instructors were like that—cooperative and accessible.

Interviewer: And the not-so-good ones?

Respondent: Mostly in research. There was too much back-and-forth. You're told to do

Respondent: Aaahhh, let me just say that, I mean this is not out of favoritism, not out of favoritism eeeennnnhhheeee, good facilitators are—meaning if there are their sessions, they come to teach you something that you need, if you haven't understood maybe, and they are… they are friendly and you are free to go and ask them anything, I mean they are accessible whenever you… you… you need them, you have something that you need to ask, you go and ask them. For the most part, the teachers who were teaching us, there wasn't… there wasn't a teacher who was not accessible eeeehhhh, all teachers were accessible, I mean there was no teacher that like if you needed them, you wouldn't… wouldn't… wouldn't find them. Except maybe in research, in research I don't know, maybe we were the ones who didn't understand. But in research aaammmhhh there was this… this… this… this person who you'd say had too many corners—you go to one person, they instruct you to do this and this and this and this, then you go and do exactly what they instructed—this and this and this and this—and then when you come back, what they had instructed now seems… I mean it seems… you find yourself… you find yourself with… with… with… with so much time discussing the same thing over and over, to the point where as students we got tired—now what does this teacher want me to do? Because they are a teacher, sometimes you don't even tell them anything, but I think maybe we were making mistakes or the students were making mistakes because there were parts they didn't understand and didn't know that they hadn't understood, that's why they kept repeating the same mistakes. So I think in the research department, they should look into how they teach students to eliminate that confusion that comes up when preparing proposals and research reports. But in other areas, our teachers were very cooperative, very accessible, and during their scheduled class times, they were there to teach. In the exams, they set questions that reflected exactly what they taught—not like setting a hard exam just to punish you. Let's just say they were cooperative overall.

Interviewer: Now when you are at work, comparing yourself to graduates of other nursing degree programs from other institutions, what unique competencies do you feel set you apart from those graduates?

Respondent: Aaahhh now here… here I would like to talk about… about the products too… even other products from Kairuki that are not nursing, like MD. Yeah, honestly, many of the products from Kairuki—many times, we communicate—and I see that these are people who, even at work, are seen as very competent. Yeah, I mean competency is… is strong. The experience—even when a student has just graduated, just finished internship—once they're on the job, they are seen as someone… as if they are a worker with long-term experience. That's the image of many Kairuki people that I've… I've seen—something that even I observe here. Yes, eehhh.

Interviewer: Okay, so when you compare yourself to graduates from other colleges, you clearly see that in terms of knowledge and in terms of competency, you are more skilled than the others?

Respondent: We are more skilled than the others.

Interviewer: Okay.

Respondent: We are more skilled because even those others you find… I mean, I can give an example—even when we were there during internship, we would find ourselves as students all at the same level, all interns, but they needed you to teach them things—as if, as if they hadn't studied. So you can see how we are better, maybe, than graduates from other colleges.

Interviewer: Now, which course knowledge or competency that you got in this bachelor's degree—what is it that helps you most in your current work? You studied many things—anatomy, physiology, midwifery, and various skills—so in your current job, which content and competencies do you use the most?

Respondent:
Okay, for now here as I told you I'm in the minor theater, eeehhh I'm in the minor theater, so most of it is surgery-related issues, meaning surgical nursing but also naaa naaa and anatomy. But also there are times I do administrative tasks, so leadership too I use it a lot.

Interviewer:
Aaahhhh what course content and competencies from the BSc.N program do you **not use** much in your current job? As you've said you're in the minor theater, what are the skills or knowledge that you acquired during the BSc.N program which you see you **don't use much**?

Respondent:
In the minor theater?

Interviewer:
Mmmmnnhhh where you currently work.

Respondent:
That I don't use much?

Interviewer:
Mmmhhh what skills that you learned or content you were taught that you find you **don't use at all**, you just studied at school but don't apply them at all in your current job?

Respondent:
Eeeeehh now for that I'll have to sit down and think because… you know, knowing that this thing I don't use, you must sit and revise, eeehhh. So I think let me not respond to that yet, maybe if you had asked what I use, I can answer because it's something I do.

Interviewer:
Eenheee for the things you use?

Respondent:
What I use, yes—like those dressings, we dress, stitching aahh those incision and drainage, and with fractures applying POP, reduction—I think many surgical issues I deal with… those that are required and within the capability of our hospital—I do them.

Interviewer:
And when you are at your work station now, can you describe your experience of working **beyond your scope**? Have you encountered any challenges? We trained you as a BSc.N, and now you're at work, do you find yourself in situations where you are expected to perform beyond your scope?

Respondent:
Okay for example, like here, like here our institution has a challenge of shortage, eenheee shortage of staff—not doctors, not nurses. So sometimes you find, you find aaaahh I'm in minor theater and there are things that—according to maybe my job description I'm supposed to do this and this and this and this and this—eenheee, so for example those POP applications are things that **they are not within my scope of practice**. Eenheee those, I don't know, incision and drainage—those are things that, even though I know, I was taught—but I'm not allowed to do them, I'm not… I'm not licensed to do them.

So, but because I understand and sometimes the responsible person is not around, and you can't let the patient suffer, not get service just because I'm not allowed to do it—as long as what I do doesn't harm the patient, I don't… I don't harm the patient—I do. Things like that.

Interviewer:
Okay, and in doing that have you faced any challenges? When you do those tasks that are **beyond your scope**, what challenges do you encounter?

Respondent:
Aaaaahhhh I can say **God helped**, there's none that I've done that later caused problems—but I think if it had happened that I did something and later there was a problem, somehow I didn't follow protocol, there's a way I would have been held accountable—"why did you do it, knowing that you were not supposed to do it." But because **there hasn't been any challenge** with those I helped, mostly I haven't had any challenge, eeehhh, I haven't had a challenge.

Interviewer:
Okay, based now on this discussion we've had up to this point, what is one **important thing** you'd like us to consider as we go to review this BSc.N nursing curriculum? The most important thing that you say, "please don't forget this when you go to review the BSc.N curriculum, make sure you address this"?

Respondent:
Okay, meaning there… aaammnhh… at first I read the questionnaire you sent—the thing I noticed from the questionnaire, even if there was no direct explanation—is that it's like you were trying to **assess the products** that have come out of Kairuki, yah, that was… it's like you're doing **evaluation**, like "these people who have come out from us, how are they doing where they are?"—how are they progressing in all aspects—that's what you're doing.

But now, there are things that I think, as an institution, you **cannot do**. For example… like creating an environment where what we are taught—as long as we know it—we can practice it directly, that's not within the institution's ability, I think. But the institution **can advise**—that's

why others have pediatric nurses, nurses with their own specialties mmnnhh. But the institution can carry that as a recommendation to the relevant ministry which has authority to do that.

But one thing I think is important when you go to review your curriculum—**tuition fee is part of the curriculum** because… yes, I think they should review that. Eeeehhh meaning **fees**—they should look for a way they can… try to minimize it to create a way where everyone can study—even if… it can't be equal to government universities because this is a self-run institution—but at least let it be such that even with its private nature, **anyone who wants to study can get access**. So **fees should be reduced**.

Here is the **word-for-word English translation** of your text:

Interviewer:
And what is your opinion regarding the establishment of master's programs, aaahhh, in nursing and midwifery that will align with the needs of the job market? You are already employed, you are there, you know the needs of the market—what the market needs. Now that you know what the job market requires, what do you advise now regarding the introduction of these nursing and midwifery master's programs?

Respondent:
Okay, one thing I advise—first of all, master's, master's, in any place whether government institutions or private, master's is... it is known to be expensive. Now if we have a vision or if the institution has a vision of starting aaahh master's programs, then it should try to also find various stakeholders to collaborate with—I don't know whether it will be partnerships or sponsorships—where the institution, let's say it wants to enroll twenty master's students among those, have... among them sponsors, find sponsors to partner with or to offer sponsorships. Create a way that students can get sponsorship. Not necessarily fully funded, but even if not fully funded, at least let there be partial support—rather than someone coming and having to squeeze themselves with 12 million to pay. So when you are planning to start a master's, you're also planning to support that student who wants to study that master's—that yes, we've opened a master's program, but we also have stakeholders who provide sponsorship for students who meet criteria A, B, C, and so on. That will also help to get students to come study—who are also our clients.

Interviewer:
And in your current work area—you said you're in the theater—what master's degrees do you think would be appropriate to start, and why?

Respondent:
Aaaaahhhhh, which master's degrees would be appropriate to start in relation to where I am currently?

Interviewer:
Eeeeeehhh, just in relation to your work area where you are working... you said you are in the main theater, and when you're not in the theater, where are you?

Respondent:
I’m present, I’m in the office.

Interviewer:
Eeeeeehhh, you’re in the office. So now, in your workplace, there are various units and departments. Among those units and departments surrounding you, what master’s degrees do you think would be suitable to start and why?

Respondent:
Okay, there are, there are many. But on our side here, our community, many people here... they use motorcycles, “bodaboda,” so we find ourselves getting many cases of fractures and dislocations and orthopedic issues, eennnheeee. Now, in orthopedics, you can’t separate it from physiotherapy. Orthopedic and physiotherapy are like siblings—they go hand in hand. Enhee. So if they can start a master’s program related to orthopedics—I don’t know if for us nurses we have that in our system—but if it exists and it relates to where I am, it could help improve the services we provide to people who come here with those bone problems, which are the most common cases—bodaboda (motor cyclists), accidents, I mean. But also, right now we are constructing an emergency department. It wasn’t modern—I can say that. So that means once it’s done, we will need a critical care and trauma team that can work in EMD or ICU. So even that specialty is needed. It has... it has demand, yah.

Interviewer:
And now in your current job, since you’re already employed, do you see any gaps in competencies for nurses and midwives who are specialists—considering the required performance at work?

Respondent:
Competencies? The gaps in competencies that I see?

Interviewer:
Eeeehhh, for these now—they are called specialist midwives and nurses—those with master’s degrees. Where do you see the gaps in their competencies?

Respondent:
Well, for me here, where I work, I can’t really speak on gaps practically because we don’t have a nurse with a master’s here. The highest rank here is bachelor, and I think there are maybe three of us? Two?

Interviewer:
And for your views now, what competencies should this specialist midwife or nurse have in order to fit into their roles?

Respondent:
The competencies they should have?

Interviewer:
This specialist—someone with a master's now—eenheee, do you feel, maybe, as someone who studied bachelor, what competencies do you feel you're missing that make you want to pursue a master's?

Respondent:
Okay, yes. That's why earlier I said that in our profession, we need to properly establish the issue of **professional boundaries** among ourselves. Eeeeeheeee. Once that is clearly defined, once that is established, then it will clearly show that "aahh okay, so if I have a bachelor, there is something that person with a master's can do that I can't." If there are no boundaries, people will pursue master's either due to personal interest in increasing their skills or just academic qualification. If we don't define the boundaries properly—for example, I think at Muhimbili there are many nurses who have master's—not many, but at least some. What differentiates those nurses—aside from those who did specialized master's in pediatric nursing, or critical care and trauma, or those who specialized in anesthesia. But others, like MPH—what will make her different from you? Obviously, she will be in administrative matters, but when it comes to practice, you find yourself doing the exact same things. Why does this happen? For example, someone studying critical care and trauma—they might be able to do a tracheostomy, they might—but me, because I'm not specialized in that, I can't. But there are some sub-specialties in nursing where someone studies and the difference is only on paper—that this one has a master's in this, this one has a bachelor—but in practice, we're doing the same thing. There is **nothing that they can do that I can't**. So I think in our master's programs—not just master's, even diploma and degree—there is no clear boundary. There are no direct lines, eeehhh. Unless we talk about specific master's one by one—you can't talk about master's in nursing generally, you have to mention them individually. Yes. I don't know if you've understood my point?

Interviewer:
Eeeehhh, I've understood.

Respondent:
Yes.

Interviewer:
Final question—based on all our discussions, as we now go to start these master's degrees in nursing and midwifery, what is the most important thing that we must consider?

Respondent:
What is the most important thing we must consider? You mean in the institution?

Interviewer:
Eeeehh, as we go now—we as HKMU.

Respondent:
Okay, I think… I think… aaahhh there are many diseases and the need is great. But let us look, let us look—let us base on **current issues**, current issues that are beneficial to the learner but

also useful for **career development**—not just for the individual, but for the profession itself. Enheeee.

Interviewer:
For example?

Respondent:
For example, aaahhhh right now we are at a point where TANNA has… has grown. You can say it has expanded in a way where now even nurses are involved in policymaking processes that involve health systems. Enheee. So when we plan to start these master's, let's also think about starting master's programs that will give us **access**—where we can be involved even in government policy development. For example, if you study something like epidemiology, or research-related fields—you have greater access, and the government may have demand for you. Now suppose the government demands you, and at the same time, you are a nurse—you find yourself with **influence**, even over ideas that may be dismissed if you're just a nurse suggesting them. But if you're in a certain **position** that the government uses, it's easier to **lobby**, even to introduce policies that are friendly to us nurses. So let's start master's programs that—first—benefit the individual student, but also contribute to the growth of our **nursing profession**. Yah.

Interviewer:
Okay Juliano, thank you very much for your willingness to do this in-depth interview—you've contributed a lot and shared many ideas. No doubt it will be a big help as we go to review our curriculum and get a strong curriculum that will benefit us and our country.

Respondent:
Well then… I also thank you all for involving us.

Interviewer:
Alright, thank you.

Respondent:
Alright, thank you too. Okay, good work.

Interviewer:
Okay, you too.

IDI Graduate #4

Interviewer:

Dear participant, the Faculty of Nursing from Hubert Kairuki Memorial University (HKMU) is in the process of reviewing the curriculum in order to meet the changing needs of both its BScN graduates and the Tanzanian people. The purpose of this study is to seek opinions of stakeholders regarding HKMU BScN graduates. We are therefore conducting this study to capture important information that will inform the BScN curriculum review. We are requesting you to participate in this IDI which may take about half an hour to one and a half hours. The information that you share with us is confidential and will not be shared in any way that identifies you. We do not anticipate any risks associated with your participation in this study. However, feel free to report any problems concerning this study to the chairperson of HKMU Institutional Research Ethics Committee (IREC) Prof. Frederick Kaijage or the principal investigator, Prof. Columba Mbekenga. There may be no direct benefits to you as a participant in this study. However, the information you provide will be used for the purposes of BScN curriculum review initiative. Your participation is voluntary. You may withdraw or refuse to participate in this study or in any part of this study at any time, and this will not result in loss of benefits or poor relations with HKMU.

The main areas include a broad overview of graduates' knowledge, skills, job performance, views and experiences during the BScN program and after graduation, challenges, and professional career development.

Have you understood the information I have provided? And do you agree to participate?

Respondent:

Yes, I have understood, and I agree to participate in the study.

Interviewer:
Aaaaaa... now, aaaaa… I would like to know you as aaahhh... A graduate of this bachelor's degree in... in nursing from Kairuki University, in what way has the bachelor's degree affected your job performance in the following aspects:- What are the challenges you have encountered in the job that you are doing? Mmmnhhh... you have gone through this degree... this... this... nursing bachelor's degree and you... you graduated, now after finishing and getting a job, what are the challenges that you can say you have encountered in the work you are doing?

Respondent:
Aaannhaaaa, challenges?

Interviewer:
Eeeeeehhh…

Respondent:
Aaahhh honestly I have no... there are no challenges because personally... after finishing internship... I got part-time teaching sessions here, after part-time I got full-time, after teaching full-time then I... I was appointed to the exam office but still also I teach in the nursing department. So for what I learned from Kairuki I don't get big challenges, although the challenge is in our... what we call transcripts—someone when you give them, they say it looks like just a piece of paper. That's the only challenge eeehhh but with what we gained, I don't have challenges up to now. We

work with the ministry, Ministry of Health, we collaborate with them, people from the ministry come, we work with them. They appreciate the knowledge we got. If you say, “I studied at Kairuki,” they say “Oh wow! So you studied at Kairuki? Aaaahhhh, you do good work,” so we work with the ministry—it’s... it’s very good things. So what we were taught—I have no challenge.

Interviewer:
Okay, so in... when doing your daily duties in your workplace, are there things you encounter maybe in one way or another that you fail to do and think maybe if you had studied them in your bachelor’s degree, they would have helped in your work performance?

Respondent:
Eeeeehhh… maybe if anything... maybe because me... me I do… like... I don’t regret that I missed something that should be added, eeehhh but for me I do... things are okay, yeah. For what I learned in college, things are okay... for me, many things help me. Maybe in the future, with time, but as of now, I haven’t seen [a problem]. Yah.

Interviewer:
Okay, and does this nursing bachelor’s degree help you in your current job, and if it helps you, how has it helped?

Respondent:
Together with everything, it has helped me **very much** because it is the one that first gave me the opportunity to work—meaning without... without a degree... let’s say from Kairuki, I wouldn’t have been able to get a teaching job, and also being appointed as an exam officer. So it has helped me greatly to get a job, to get a chance to develop myself in teaching—meaning, if I was just sitting jobless, I wouldn’t have been able to teach or use the knowledge I got. So it has helped me very much. Through the nursing degree, I help my family, meaning without it, maybe I wouldn’t even be able to help my family significantly, and myself too. So it has helped me a lot. I’m also continuing to develop myself, I keep teaching, I keep studying—I can’t teach without studying. I must study, read different books, go study—so it has helped me greatly.

Interviewer:
Okay, aaaahhhhh and… what are your future expectations or plans?

Respondent:
Yaaahh... Plans in what? There are many plans... either in my career or? Because there are many expectations.

Interviewer:
Eeenhee, how do you see this nursing degree you got—do you think it prepared you for advancing your career?

Respondent:
Aaahhh... okay...

Interviewer:
Mmmnhhh… because now you are our bachelor's degree graduate and you finished... now how do you see this degree prepared you for the future in advancing your career?

Respondent:
Aaahhh... first of all, it prepared me to become... become... become a tutor and... and until now I am continuing to develop my career by teaching diploma and certificate level students. And me also to go develop further... I participate in the exams of... of... of TNMC—I am one of the people supervising board exams. Even recently we were there... So it has also helped me to prepare new graduates and I also have plans to continue studying, to continue advancing our profession.

Interviewer:
Mmmnhhh... so your future plans include furthering your education?

Respondent:
Yes, absolutely continuing to study, yeah, and... and my big expectation—I don't think it's bad to say it here—is that there will be Mwanza University. So, I will also continue by going to teach there along with furthering my studies.

Interviewer:
Okay, and... and now for example, can you propose what changes should be made in this bachelor's nursing program here at Kairuki?

Respondent:
Aaahhh, the big proposal is... is to add... in what? Theory or practicals?

Interviewer:
In both, theory and practical.

Respondent:
Aaahhhh, theory and practicals?

Interviewer:
Eeeeehhh...

Respondent:
Aaahhh… there are those... those trends in nursing, make changes in the trends a bit—they don't really build up... they take a long time but also they don't really help the student much in developing further. So I wouldn't recommend it be there. Also, for practicals, the time of... of clinicals should be increased and the time for the skills lab should be sufficient.

Interviewer:
Okay, meaning the practical time should be increased?

Respondent:
Yes, it should... it should... although even during internship, most new graduates—even those we receive now—have a bit of a challenge in practicals.

Interviewer:
Aaahhhh... they're not very good?

Respondent:
Eeehhh... so we should increase practicals a bit.

Interviewer:
Okay... and on trends you said?

Respondent:
Aaahhh... trends... I don't think they help anywhere.

Interviewer:
Eeeehhh... why?

Respondent:
Ahahahahaahah (laughs)

Interviewer:
We like to hear your opinions now so we can work on them.

Respondent:
On... on the things we study in trends... I find I don't... aaahh... we don't really get how to apply them anywhere, yes.

Interviewer:
Aanhaa... okay, so it means for example the things you studied in trends, for example now when you work, you don't see how you apply them in your job?

Respondent:
Yeah, exactly... I don't see how.
But also increase more... make sure it's in... that educational psychology—yes? That one is very good.
Many nurses when they come to teach, we struggle there. I think that one, add more emphasis on it... eeehhh... because many nurses—I'm not a trained nurse educator—I'm just a nurse per se, but I got a chance to teach students. That knowledge of educational psychology—how to prepare... how to prepare a class—helped me a lot, so strengthen it more and you'll produce more teachers who will help students here.

Interviewer:
Okay, when you say add more emphasis, in what way, maybe what's your suggestion—what should we do?

Respondent:
Practice—like for example, give students... maybe you can give them during those sessions, have a practice day, even to go and teach in a real classroom—maybe certificate students, I believe you have them—just let them try to teach, yes. If not in the skills lab, let them try to teach so you can help them improve their teaching—it will really help.

Interviewer:
Okay, alright—and do you have any other issues you'd like considered during the review of this BSc.N curriculum?
Aside from those you've already said?

Respondent:
Aaahhhh... I don't think... I don't think I have others eeeehhh... if there are others maybe we'll contribute later, but... but... but those are the ones. Yes. I don't think there are others... many things are okay, many things are very okay and have helped us grow—they are okay... eehhhh...

Interviewer:
Okay, and now... now that you finished your internship, what challenges did you encounter when looking for a job?

Respondent:
Challenges during job search... eeehhh... for me personally, because I connected... eeehh... after... after... during internship, we stayed... we stayed... there was a small session of teaching. We came to teach, after teaching eeehhh... they said why don't you get a session—the head of the nursing department—get a teaching session, so after finishing internship, I continued. The only challenge I encountered was the **transcript**. Our transcripts, please improve them—when students are asked "you studied at Kairuki?" they say "why does your transcript look a bit off?"—like... there's a certain way the transcript is supposed to appear at least...

Interviewer:
Mmnnhh, what do you think—how would it have been, how would it be better?

Respondent:
Aaahhh... let's see, like how when you look at how the ones from NACTE come out, you know at our level, yes we teach things, they issue transcripts similar to those from NACTE, certificates like NACTE's—if you look at it, the transcript looks like NACTE's a bit.
When you give it to someone, they say yes, it ha... it haa... it has something.

Interviewer:
Mmnnhhh… this one that you are given, what shortcomings does it have?
Maybe they haven't included all the things? All the subjects you studied? Maybe they haven't shown the marks you got?
Or what things are missing? These transcripts that you are given?

Respondent:
Appearance, that is—when you look at its appearance, it looks like... when you make a copy...

when you use it... when you print it, it's just like a paper—plain, just like someone issued provisional results.
Eeehhhh... it's no different from someone asking for... it's like provisional results, but when you look at the certificate, when you give it to someone, they say yes, this is a certificate.
But when they ask for your transcript, it's like you just gave a piece of paper, like... just like plain paper with results filled in, like a provisional result.

Interviewer:
Aaanhaaa... and is this something employers have also been... been questioning when you go looking for those jobs?

Respondent:
Aaahhh me, I was... I was asked, he said, "Why is your... your document... when... I had already gotten [the job], but after getting it you must send your documents."
He asked me, "Mmmhh... why does your transcript look like... like provisional results?"
Eeehhh… not like a transcript. Someone when they see it, even if you say it's a copy, they say yes it's a copy but why does it look just like plain paper?
Eeeehhh... so please help us there to improve it. But otherwise, other things are okay—congratulations.

Interviewer:
Okay, thank you. Aaaand... now, what do you think could have been done during your bachelor's training to better prepare you for the job market?

Respondent:
Aaahhh... in preparing for the job market, one of them is exactly such things, yes. You teach us a lot of knowledge—enough, eeehhh... but one of them is those small things.
Because when you meet people who come with documents that are complete, it's possible yes someone will be satisfied that this is a transcript, really—it has a transcript look.
That a little would make us... would make us better in the job market.
Eeeehhh... and the other thing is just to move with time, like you're doing now—coming now to prepare... to review the curriculum.
Meaning, you've... you've taken our views, and that's one of the things—when we tell you these, they'll help in... in... growing... they'll help you produce eehhhh graduates who will... will compete in the job market.
Yeah. And... and also something else eeehh... what did I want to say...? I think that's it.
Eeehhh... even though the job market now, the challenge is for... for the whole country—people are many now, honestly.
Yes, people are very many, I don't think... that we should blame our college so much... people are many and we cannot deny the challenges that exist honestly.
Because sometimes it's... the number of people needed is maybe ten, but the graduates are many.
So we can't complain too much about our college... but the college taught us very good things—I'm proud and they help us.

Interviewer:
Okay, aaaand... now, I'd like to know your experience when you were getting this BSc.N training

here at Kairuki—what things did you like about this nursing program, and what things do you feel need improvement?
Let's start with the things that you think were good and that you liked about the BSc.N program here at Kairuki.

Respondent:
Aaahhh... I liked the skills lab. That was fantastic. Skills lab—honestly, I liked it very, very much. It was building us—the skills lab really builds us, it really builds the student toward going to the patient. So it's... it's... it's a very good thing, I really liked it, I was very happy during the skills lab sessions. And also there were computers—you could look up anything that challenged you, see how it's done—it's a very good thing. I also liked the clinical area—eehhhh, the time was a lot in the clinical area… veryyy much... I mean… Very much. I'm very proud even today—especially at Temeke... OBS and Gyne... Temeke and Amana it was fantastic, along with paediatric in nursing— Eeehhh... the clinical area was very good and the follow-up was very good. It was a very, very good thing, honestly. Also that mixing... and another thing that... that mixing of students from year one up to year two in subjects like fundamentals— It's a very good thing for this college—it's a very good thing. It builds big cooperation among nursing students and also the medical students. I... I liked how most teachers really dedicate themselves—maybe it's changed now, there are new ones, but most teachers really dedicate themselves to students. That is something rooted in the teachers—so please maintain that, it will... it will really help students. Yes.

Interviewer:
Okay, and what are the things now that you feel need improvement?

Respondent:
I don't know for now... student transportation. Student transportation to the clinical area—it's a very good thing. Yes, it's a very good thing—to go to the clinical area. We see other colleges—even we take our students, and we also see our neighbors here in Mwanza-Bugando, they are our neighbors—they have vehicles, they take the students, they go drop them off. That is a very good thing madam—if you haven't done it yet, please do it. Yes.

Interviewer:
Mmmnnhhh, and is there anything else that you feel needs improvement?

Respondent:
Aaahhh... something else... something else is in computer—I don't know currently... That is communication skills, computer communication skills—improve how students... how to make PowerPoints—add those things a bit. Eeehhh... I mean... make them align with how this student, after they graduate, if they go to teach as a nursing or medicine student, they should be able to even prepare a PowerPoint. That thing really... really challenges us, yes. How to prepare these... even how to type things on the computer—add that a bit, it was lacking when we were studying. Eehhhh... those things add them under computer skills. Others, the student, depending on... on... on today's needs—when they go to teach like me, most of the time they'll need to use PowerPoint, they'll need Excel, Word... Let it help when they go... when they go to the ward, they'll need more to use these health service systems—students, if possible, should be taught there. Yes.

Another final thing is in leadership—many students, we found ourselves in leadership, there's a bit of a challenge— Like in practice, we fail—you find a student, you give them, prepare... prepare... a duty roster, and it's a bit hard for them—add more there. They should be told that as a leader they should do what? You ask yourself, in leadership, so what did I study to become? Just a tutor or what? Add a bit there—explain to students, as a leader what should they do? As a supervisor, what should they do in the ward? Yes.

Interviewer:
Okay, so if I understood you correctly, you mean that this student should be exposed to the real roles of a leader where they will be after graduation?

Respondent:
Yes.

Interviewer:
Mmmnnhhh okay, and you had mentioned something about communication skills—I didn't hear you clearly, what did you say should be done about communication skills?

Respondent:
Aaahhh... the communication skills that we were... we were studying, gave me a bit of a challenge—maybe I didn't mention it earlier... It challenged me in preparing PowerPoints. I mean, there are some things—considering that we're being prepared to later become nurse educators, right? That they are being prepared as nurse educators—they need to know how to prepare PowerPoint. They need to know how to prepare Microsoft Word—know it well. If we need them to go teach—if we need them, when they are in the ward, to use computer things—how will those computer things help them when they're in the ward? Meaning, for example, if told to enter data in a system—they should at least understand how to use a computer. Soo... have you... have you heard me madam?

Interviewer:
Eeennheee, you got cut off a bit.

Respondent:
The way we study that computer, it should at least be linked with the student—for example, when they go to clinical, when they go—say, when they go there to Kairuki Hospital, rotating—if they're told to enter the patient information system, they should at least have the understanding of *how* to enter something like patient information. Yes, that's the meaning of computer. If they've gone to teach like us here, they should be guided at least on how to use PowerPoint—how to prepare PowerPoint. If it's… yeah, things like that.

Interviewer:
Okay, alright. So now, while you were studying here in the BSc.N program, you encountered different facilitators, right? You met various facilitators—teachers, from first year to fourth year until you graduated, right? So now, please tell me the qualities of the one you saw as the best facilitator and the qualities of the one you saw as the worst facilitator. Let's start with the best

facilitator.
The teachers that you saw were the best—how did they present themselves to you?

Respondent:
Aaahhh… starting from first year or…?

Interviewer:
Eeeehhh… from first year to fourth year.

Respondent:
Good teachers—starting from first year, eeeehhh… Lufukuja was the best—very best lecturer. Yeah, Lufukuja.

Interviewer:
Mmmnhhh, why do you say he was the best?

Respondent:
He really knew how to teach, yes, Lufukuja really knew how to teach, very well…

Interviewer:
Okay, when you say he knew how to teach—how exactly did he teach?

Respondent:
Enheehehehehe (laughs)

Interviewer:
Like, the way he presents the content? The way he links…? Please explain that a bit more because I…

Respondent:
The way… the way he prepared his notes—when you saw the notes, okay, when you saw how he prepared his notes, you would understand immediately. He used to have many pictures—once you saw them, you'd know, "Eeehh, yes, this thing," meaning the student seeing it would know, "Aaahhh, so this is it." That thing built me a lot and it… it became a technique I've used myself quite a bit.

Interviewer:
He put many pictures in his slides?

Respondent:
Yes. He would ensure that if there were explanations, he'd also include a picture to help the student link. So he was… Another thing, he was knowledgeable—he had knowledge about what he was teaching. Another, he was capable—he knew, I mean… he had ability. So… Another—should I only say one?

Interviewer:
Okay, now… now let's do this—since you'll be describing the best and the worst qualities, just describe them without mentioning anyone, okay? Just tell me that maybe the teachers who were best facilitators, like you said, included pictures, were knowledgeable—things like that. Then we'll move to those who you saw as not very good facilitators.

Respondent:
Hahahahaha (laughs) I don't think there's a… a… a worse… I didn't see any, by the way. So, should I continue with the good qualities?

Interviewer:
Eeeeeee… let's continue with the good qualities first. Enheeee… so?

Respondent:
Another point… most teachers during clinicals had a big ability to follow up with students—to know if they were attending. You know a student is a student. Eeeee… many teachers had the heart to go to clinicals, to check on their students and guide them. Yes. Also… also, not only clinicals—also during field placements, community field, teachers were committed to come guide us on what to do. Eeeehhh… that's a very good quality. Also the teachers… during those lab practicals, they were very committed, they… they taught and they knew those things practically. Eeeeehhh… in many subjects, those practical contents—they understood them and helped us a lot. Yaaaani, they had a lot of knowledge. So even the scheduling of sessions was very good. Yes. In eeehh… I think improvement is needed on… on… I think not 'worse', just… just a point of improvement—let's not use the word 'worse', ahahahaha… It's not worse—it's just an area to improve: some exams repeated exactly the same. Eeehhh… That thing, if it's there, please remove it. Repeating exams doesn't help students grow. Yes. And also in private exams—you'd hear, you might hear like during fundamentals, you'd hear the exam… leaked a little bit. Just control that exam issue. Eeehh… although it was mostly in fundamentals back then when it was… a mix, yes.

Interviewer:
Aanhaa… so during the time you were taking classes together with MDs?

Respondent:
Yes.

Interviewer:
Okay.

Respondent:
Eeenhhhee, so a little bit the exam… your exams—make them more… more confi… confidential, enhee… especially in fundamentals exams. Another thing in the best performing area is the cooperation—cooperation between teachers and students. The relationships were very good. We left there ourselves having very good relationships with teachers. That's the best—congratulations, madam Monica. I'll mention personally Madam Monica, Madam Minael, eehh Madam Mika, Sir Shidende, and others—the relationships were very… very good.

Interviewer:
Okay.

Respondent:
In research, I'll say—eehh, when we were doing research, a bit… our supervisor, I won't name names, my own supervisor was using an outdated reference year. So that a bit… tell me, when you look at a reference from an old year—like to judge a student currently—we should use things that are more current. Yes.

Interviewer:
Okay, so they were using references that were very old?

Respondent:
Aaahhh… very old, yanii… I was really wondering—so outdated, and now… If you're using that to guide a student on how to do something, at least use something more current.

Interviewer:
Okay. So when they were giving you input for your research, they were telling you to go review things that were very outdated?

Respondent:
Eeeeeheee… like very outdated. Eeee… so when you look, if it's from old years, a little bit it doesn't align well with… with current contexts. Yes. Finally, most teachers have those lecturer qualities—yaaaaniii… many have them… there are many.

Interviewer:
Mmnnhhh, like which ones? Those qualities?

Respondent:
Aaahhh… hahahahahah (laughs) I think I've already said—knowledgeable, capable, eeehhh… A teacher who is accessible—when you need them to guide you, they are accessible. Yaani… competent. Most teachers were competent—yaaaani, very well-educated. Yes.

Interviewer:
Thank you. Now, when you compare yourself—because you're meeting other BSc.N graduates, right? When you compare yourself with graduates from other colleges of the same degree—what unique abilities do you have that differentiate you from graduates of other colleges? What thing or things unique have you gained by studying the Bachelor of Nursing here at Kairuki that you feel graduates from other colleges don't have?

Respondent:
Educational psychology. Yes, educational psychology helps me a lot in how to teach. I was taught by Madam Mika, I think… that one… that one differentiates me a lot from… from… from… from other graduates. Yes. Because my way of teaching—personally, because I spend most of my time teaching, I deal with student issues. So… another thing is just that ability… the things we received from Kairuki— We're competent. Yaani, they produce competent graduates—there's no problem.

You leave knowing, “Eeehh… yaani, we know,” you say, “Yes, this will help me.” Because of getting a lot of airtime rotating in the first unit—something that’s within the hospital, so it’s right there. So it makes us a bit different from many students… others. Eeheeee… yes.

Interviewer:
Okay, so for example, other graduates you’ve met—their curriculum didn’t include educational psychology?

Respondent:
Eeehhh… some don’t have educational psychology. So sometimes you find someone can’t do it—you have to start showing them, “Do this, we prepare this, how to prepare a class, how to role model inside the class, let students do role model…” So inaaa… some of them don’t have it.

Interviewer:
Okay. Now, what things have you studied—as content, knowledge-wise and competencies during your BSc.N training here—that you currently apply most in your work setting?

Respondent:
Ooohhhh… many things I apply—eeehhh… that I studied. The main one is… is teaching—because I teach a lot, madam.

Interviewer:
Mmmmm… so you mostly teach?

Respondent:
Eeehhhh… for me, it’s mainly teaching. I teach and work in the exam office—dealing with exams. Yaani, if you tell me exams, I know. If you tell me curriculum, these… those I deal with a lot. And exam guidelines—especially those of NACTE—I deal with them and teaching.

Interviewer:
Mmmmmnnhhh…

Respondent:
Eeehhhh… so I mostly just apply that… the thing I saw my teachers doing is exactly what I’m doing now—how to teach, how to prepare things… I think I won’t mention others too much. Maybe… maybe clinicals sometimes—patient care is rare, mostly when I take students there. But most of the time it’s teaching.

Interviewer:
Okaaaayyy… okay, and maybe here, what subjects do you teach more?

Respondent:
Teaching… for example, like right now I have… I have… it’s newborn—right now I have the subject of newborn with abnormal and normal conditions, which, due to the other tasks that I do,

so I only have one module that I teach there. Eeehhh, so I mostly teach… for example, since I came, two main subjects: obstetric emergency and… and… newborn with abnormal conditions. Eeeheee.

Interviewer:
Okay, so specifically, one of the skills you said you use more, which you learned during BSc.N training, includes teaching and educational psychology, right?

Respondent:
Yes, the way of… that subject that's related to teaching.

Interviewer:
Mmnnhhh.

Respondent:
Eehhh, so for me, that's what I mostly use. In the clinical area, maybe when I've taken students there, maybe to… to see how they're doing—but not a lot.

Interviewer:
Okaaaayyy… alright. Aaahhh, and which content or competencies that you learned during your BSc.N training do you *not* use much in your current job?

Respondent:
Which I don't use much…

Interviewer:
Mmmmhhhh… because you've said you use educational psychology more, hehehehehe (laughs).

Respondent:
It's the principles… nursing principles—all of them I think I… I don't use much. Things like drug administration—I don't really use many of those in my case.

Interviewer:
Mmmnnnhhhh……

Respondent:
Eeenheeee… I teach the principles but I don't really use them, honestly. Eeeheee. Yes.

Interviewer:
Okay. Mmnnhhh, okay—so those PNU things you don't use much?

Respondent:
Aaahhh, I don't use them much—I would be lying.

Interviewer:
Eehehehehehehe (laughs)

Respondent:
Yaaaaniii… hahahahah (laughs) so if you ask me that—it's very rare. Although, from afar, your subject—OBGY—I do sometimes go to Sekou Toure Hospital, and if I find students working, I check them a bit, I do something. Eeeheee.

Interviewer:
Mmnhhhh… okay, so in other words, most of your time is spent more at the college, but in the clinical area you spend very little time—just when you go to supervise students?

Respondent:
Yes, when block study ends… when they go into block study—block study is clinical. I rarely go—most of my time is spent teaching during block study. We spend a lot of time during block study.

Interviewer:
Mmnnhhh… okay, so when students are there on block study, they are with instructors in the wards—you teachers don't go much?

Respondent:
We teachers don't go much—they're with clinical instructors while they're at the practical site. They have a logbook—they get signed there. So we just go occasionally, maybe once or twice a week. Like I told you, I also have another office—the exams office—so as you understand, in the exams office there are many things. So for me, clinicals are… are very minimal. Most of my time I'm teaching.

Interviewer:
Okaayyy. And now, in your work, can you describe your experience doing tasks beyond your scope?
I mean, you find yourself doing duties bigger than… than what your job description says?

Respondent:
Aaahhhh, heheheheeh (laughs)

Interviewer:
For example, you are an educator—now in your workplace, have you ever been given responsibilities that are beyond—because you as an educator have your specific roles and responsibilities—have you ever found yourself doing tasks meant for someone with a master's, or something that's not in your scope at all?

Respondent:
Aaahhhh, no, not in teaching. No. Because most of the students I teach are certificate and diploma level, along with basic technician, who, according to the curriculum, I'm actually the one meant to teach them. So there are no bigger tasks that a person with a master's was supposed to do. Eeeehhh… maybe just those tasks—you might find you're assigned something like… like I said earlier—you asked me if I do something else. I also work in the exams office, which sometimes needs someone who studied documentation, or… or certain things. But I'm also someone who

studied nursing. Yes, maybe just that—but as for teaching, I'm okay. We don't have students of master's level, or advanced diploma or degree that I'm teaching. No. Eeehhhh.

Interviewer:
Mmnnhhhh… so you also said you hold the exams office, so… so it's like you're… you're the examination officer?

Respondent:
Yes.

Interviewer:
Okay. Mmnnhhh.

Respondent:
Yes, yes.

Interviewer:
Okay. And when you're holding both offices at the same time—you're a teacher and an examination officer—what challenges do you face?

Respondent:
Challenge is tiiime… the time for teaching. There are times you'll be in class and you're called—"We need you in the office." Eeehhhh. There are times you're supposed to go to clinicals, but there are things you need to handle there. Yeah—the challenge is that overlap. So when you're supposed to be in class, you find yourself doing office work—like exam office work. So at times, you leave the students like, "You guys, do a discussion or I'll come tomorrow." Eeheeee.

Interviewer:
Okay. Now, from the discussion we've had so far—

Respondent:
Eeehhhhh

Interviewer:
From the discussion we've had so far—

Respondent:
Yes madam.

Interviewer:
Eeeeehhh… what issues do you advise us to consider as we review this BSc.N curriculum?

Respondent:
Aaahhhh… like I said earlier, yes—I said, you know, right now we're moving toward something that's more… We're moving more toward *skills* than just… just theory. Eeehhh, you must also

improve on… on skills. When students graduate, they should be more skilled. Eehhhh, rather than just knowing theory—knowing it well—if you ask them, say, about cancer, ovarian cancer—they understand. Or uterine cancer—they understand. But in skills—nothing. So improve more… as you review the curriculum, improve more on students' skills.

Interviewer:
Okay, and what would you suggest we do to improve students' skills?

Respondent:
Improve the skills lab… make it bigger. The current one is a bit small. Eeenheee. Also, like I said, the student vehicle—for going to rotations. There are students—truthfully—they wouldn't go sometimes because of transport. Dar es Salaam's transport is tough, I know you understand. You leave at 7 AM and reach Muhimbili or Amana at 9 AM. Yes. So if there was a student vehicle, they would get to the practical site on time. Yes.

Interviewer:
Mmmhhhh, okay—so we should improve the skills lab. By… by adding what, maybe?

Respondent:
Ahhhhhh… there were manikins, they were few. Eeenheee. The manikins that were there—truthfully—you find that there were… there were too few. And there were some manikins—like for delivery—I don't think we had them. They were very few. Manikins for… for resuscitation—cardiopulmonary resuscitation (CPR)—there was only one. Add those accessories in the skills lab. And make it bigger, like I said.

Interviewer:
Okay, okay. So improve the skills lab, the student vehicle for taking them to clinicals. What else?

Respondent:
Yes.
I think also in the pharmacology practicals—I don't know how you'll do it—Some of the things were very few. The medicines we were learning about—they were very few. Just those for… yes. So improve there a bit too.

Interviewer:
Okay. Now, I'd also like your thoughts on starting these master's programs in nursing and midwifery that align with the needs of the job market.

Respondent:
At first, I didn't catch that—what did you say?

Interviewer:
Aaahhhh—I was asking your opinion about establishing master's programs in nursing and midwifery here at Kairuki that align with job market needs. What master's do you think we should start here that match the needs out there?

Respondent:
Aahhh… will you give me time or should I answer now?

Interviewer:
Aaahhhh… you can just say—maybe based on your experience after graduating, being employed—you've seen things—what master's programs could we start that are in demand in society?

Respondent:
Aaaahhh… community health.

Interviewer:
Aaaahaaaa, master's in community health?

Respondent:
Yes, community health, eeeehhhh, that one is… is… is important.

Interviewer:
Okay, why is it important?

Respondent:
Along with maternal health, yes.

Interviewer:
Okay, why do you think… you said community health and maternal health are important?

Respondent:
Maternal health… and… and I know you understand—competent nurses are needed who will be… in… in caring for women coming to give birth. Still, yaanii, there is still a shortage of professionals, yes, in caring for mothers—especially on the side of nursing. You'll even find in these hospitals, finding a nurse who has a master's is very rare—especially in labor and maternity in general. You might find someone they say is experienced, like an older woman or man with experience, but one who is… is… competent, has a master's, knows what they're doing—many are doing things based on experience. So in the end, you'll depend more on needing doctors who are specialists, yes. In community health nursing, those people are still needed—there are many challenges in… in… in the community, yes. People are needed—people to go into communities to educate them on health matters. So those people are needed—it's also a good program. Another one is anesthesia.

Interviewer:
Mmmnhhh, anesthesia?

Respondent:
Yes, anesthesia—those available, if you look currently, are just people with certificates only. Meaning they finished a diploma, or it's a clinical officer who completed a diploma.

Interviewer:
Okay, fine. And in the place where you work—aaaahhhh—you work at a college, right?

Respondent:
Yes madam.

Interviewer:
Eneheee. Aaahhhh… do you think, if you were to introduce master's courses—apart from those you've mentioned—what other ones could be introduced? Besides community and maternal?

Respondent:
Aaahhh, you know, for us here it's… hahaha (laughs), it's private. And it's people who—you know, I can't say we would introduce them. It depends on the needs. But you know it's a teaching college. Mostly for us… if they want to introduce anything, they'll actually start with degree programs first. Enehee. So about master's, I'd be lying—it would be another course… We're still at diploma level. Actually, if there's anything to be added, they'll move up to degree. Yes, which is probably where I am now. So we're still down here—we're still at NACTE. So if I tell you maybe about master's programs, I'd be lying.

Interviewer:
Okay. Aaahhh, where do you see gaps in competencies in the roles of nurses and midwives? You… you… you work with your colleagues—probably all of you there are either nurse-midwives, right?

Respondent:
Yes.

Interviewer:
Okay. Now, as you're performing your roles—you and your colleagues—when you observe, what gaps do you see in their competencies, in their skills? What things are missing?

Respondent:
Eeehhhh… the things that are missing…

Interviewer:
Mmmhhhhh. Like, as team members—your fellow colleagues—what are some things you can say are lacking?

Respondent:
Do you mean based on the things we teach or…?

Interviewer:
Eeeeeehhhhh…

Respondent:
Like maybe there where we go with students to clinicals?

Interviewer:
Eeenheeee, even there, yes—even in clinicals.

Respondent:
Aaahhh, some—during clinicals, here, many… many have… have experience. And many of the nurses we find—they are… they are our seniors. Clinicals—there are some nurses, their knowledge… yaanii, many rely on experience. Even if you bring them students, they don't explain how something works. They just say, "I have experience; here, we do it like this." Some are not very competent, eeheee, because they take things lightly. And sometimes they say they don't need to know more, because maybe there's a doctor who will… who will… who will know more. Yes, eeeeehhhh.

Interviewer:
Okay. So they just do it as a routine, but they don't know *why* they do those things.

Respondent:
Yes. Sometimes if you ask someone why they do something, they say, "aaaahhaaaahhh, it's just how it's done." Yes. But on the teaching side here, many teachers are… are… things are okay, depending on the level. Those of NACTE, the degree levels they're teaching—so many things are okay. Eeeeheeeee.

Interviewer:
Okay. Now the very last question.

Respondent:
Yes madam.

Interviewer:
From all the discussions we've had—what's the most important thing we need to consider as we go to establish master's programs in nursing and midwifery?

Respondent:
Aaahhhh, the most important thing to consider when you want to start is… is teachers. Yaanii… in terms of teachers… you must really have teachers. That's the most important thing. If you don't have good people to teach your students, you will produce graduates who are not competent. Yes, you must look at the availability of teachers and the competence of the teachers. That's the only very important thing you must look at. Yes.

Interviewer:
Alright, then. Thank you so much for your cooperation. Thank you for agreeing to do this interview—it took some time but, well, thank you.

Respondent:
Thank you madam. I'm also happy—we've talked for a long time.

Interviewer:
Eeehhhhh, very long.

KII Educator #1

Interviewer (I): First of all, let me begin our discussion by thanking you very much for taking the time to speak with us. You are a person with many responsibilities, and we appreciate that. Aah! I believe you are familiar with Hubert Kairuki Memorial University (HKMU), so perhaps I'll begin by asking: what is your opinion about the **quality of undergraduate nursing education** for HKMU graduates?

Participant (P): Ah! Yes, I do know the university and... to some extent I might say I may not be in the best position to fully comment on the quality. Because I think I've only met a few graduates from Kairuki—mainly those in the Master's programs that I know of. And those few whom I have met, especially those in Master's programs—we tend to evaluate their background to see if it allows them to **cope and succeed** in a Master's level program. So, among those few I encountered—actually, one of them I didn't just meet; I studied with him—he had a good capacity. His **overall capability as a graduate of a BScN program** was quite good. So... I'm a bit limited in my input on this, because I haven't interacted with many of them, but what I can say is... they seem to be doing well. Also, I've met a few students during **clinical placements**, and from that interaction I've also seen that they seem to be doing well.

I: Aha! If I understood you correctly, let me ask—speaking now as a senior leader—what are your thoughts on how we should support **undergraduate nursing graduates** to further their **professional development**?

P: Mmh! Ah! My recommendation is... I think from a broad academic perspective, our graduates—or those from our institutions—especially in professional programs, must **demonstrate some key core competencies**. I'd say these are competencies that enable them to meet the current **market demands**. There is a strong demand for **hands-on ability**—that is, psychomotor skills. These include both the **basic/fundamental nursing procedures**, like catheterization, which every nurse must master, but also more **specialized kinds of skills** depending on today's healthcare needs. For example, we currently have very **specific care areas**—neonatal care is one. Neonatal deaths are high, and we see the government establishing **neonatal intensive care units (NICUs)**. We expect graduates to be able to **provide care** there and to **work with technology**. We're also facing a rise in **non-communicable diseases (NCDs)**, which require some **technological interventions**. So apart from the basic nursing skills, we also expect graduates to be able to **demonstrate higher-level abilities**. And I think the biggest area is **soft skills**—things like **decision-making**, **clinical judgment**, **communication**, and **presentation skills**. Because now, the degree program qualifies graduates as **nurse officers**—and once you are an officer, you're a **leader**. Therefore, these soft skills must be **well demonstrated**. So, if there's any plan to **revise the curriculum**, I believe that's the direction we should take, yeah.

I: Mmh! Now let me ask: What is your opinion about the connection between the **bachelor's degree program in nursing** and **professional competence** or performance?

P: Okay! Ah... Are you referring to the picture of the graduate?

I: I mean, like in terms of communication skills or human relationship skills.

P: Yeah! Because when you enter the workforce, **practically speaking**, there are certain **core skills** you must have to perform well. Our nursing roles place us in positions where we **work with other people**. So, **human relation skills** are a must—you need to build good relationships at work. You also need to be able to **build teams**. **Teamwork is crucial**. That means the **curriculum and the overall implementation approach** must prepare this graduate to **work well with teams**, build positive working relationships, and practice **effective communication**. Because all of these contribute directly to their **performance** once they begin work. They can't function in isolation—they'll always be working in teams. And if they're a leader, they must also know how to **communicate with others**, and so on.

I: Aha! Do you also see any link between the nursing degree program and a graduate's **ability to teach, learn, or perform hands-on tasks**?

P: Yeah! There is a connection. Because when we talk about a graduate nurse, we expect them to have—let's say—three or four major competencies. They must be able to **perform nursing duties**, they must be able to **teach**, they must be able to **lead**, and they must be able to **conduct research**. So, within the curriculum, or in whoever completes it, we must see those competencies. We must graduate someone who can **demonstrate competence**—whether it's **psychomotor**, or if something in their care requires **improvement**, they should be able to **research** the issue. If they're placed in a unit or some other position, they should be able to **lead others**. And they should also be able to **teach**, because—at that level—they'll likely work with **junior staff who will need direction and training**. So yes, **teaching as a skill is very important**.

I: Mmh! What **skills** do you think current bachelor's degree nursing graduates **lack**, especially when they're in training or during the clinical experiences we conduct today?

P: Mmh…

I: Mmh! What specific **skills or abilities** are they missing?

Participant (P): I think we're still seeing challenges, especially in the area of **soft skills**—let me start there. Soft skills like **clinical judgment**. Because, aah, a graduate who is going to work at the **officer level**, in a **practical, operational role**, will need to have **decision-making ability**. And by decision-making, I mean decisions related to **patient care**, or **resource management**, or **planning and organizing services**. They also need to have strong **clinical judgment**—and this is a soft skill that **must be instilled during training**. They should have the confidence to say, "This is the diagnosis," and even review what the doctor prescribed—especially if it's pharmacological management—and advise: "I think this needs to be evaluated differently," and give proper recommendations to the doctor. Or when it comes to **investigations**, like lab work or imaging—if an intern comes and writes up a test order—the nurse should have the ability to **assess that request**, and based on the **nature of the illness**, say, "I don't think this investigation is going to help the patient right now." They also need to have **evaluative skills**, because at the end of the day, it's the nurse who **stays with the patient**. The doctors come in, do their prescriptions, assessments, and whatever else, but when treatment or management begins, it's the nurse who **monitors and evaluates the outcomes**. So if the nurse lacks **evaluative skills**—being able to track the patient's baseline, understand what intervention was made, and what outcomes are expected—it can really

affect care. Another major gap I see in many of our graduates—perhaps even those from your institution—is in **professionalism and ethics**. For instance, when you meet a young graduate today, they may **not even greet you** properly. They're dressed in whatever they want. Their nails aren't trimmed; their haircut is done however they feel like—it doesn't reflect a professional image. And the big question is: **Aren't they taught these things during their training?** So I think these are some of the gaps that also need serious attention.

Interviewer (I): Now, speaking of gaps—what do you think the BScN graduates from Kairuki **lack academically**, or within their **current program**, that we should address when we review the curriculum?

P: Aha! Yes—let me speak, reflecting on the few that I've interacted with. I may not generalize too much, but since this is a **qualitative study**, I believe it's fair to say that the **main issue lies in clinical judgment and decision-making skills**. These soft skills… Among the few I've encountered, they're generally good in **hands-on skills**—although not always to the level we expect—but where I see the biggest gap is when you sit down and talk to them in a clinical setting. Let me give an example—even with **interns from Kairuki** that I've worked with. In a **teaching round**, if you ask, "Why do you think this intervention was done for this patient?"—you expect a degree-level graduate to have **some level of clinical reasoning**. But often, that reasoning is **missing**. So I think one solution is to provide a **stronger foundation in basic sciences**—because that's what helps form **rational thinking**. This is a **Bachelor of Science**, after all. And with that name, it implies that the graduate must be rooted in **scientific rationale**. They need to understand **why** something is done—not just that it's done. If a student checks a patient's blood pressure and finds it at 150, they shouldn't just report the number—they should ask: "What does this mean?" The graduate should be able to say, "This is **tachycardia**, it could indicate this or that, and this is the required management. If this isn't addressed quickly, the patient might face serious complications." That's the **level of reasoning** we expect. So yes, a solid foundation in **basic sciences** is essential. And when they get to **clinical courses**—like medical-surgical nursing—we need **stronger teaching strategies** that specifically **trigger clinical judgment**. That includes **case-based teaching**, not just procedural demonstrations. If the student is managing a patient with **diabetic ketoacidosis**, they shouldn't only know how to insert a cannula or administer insulin. They should be able to **explain the reasoning** behind their care decisions: "Why do I start with fluid therapy?" "Why do I monitor the blood sugar next?" "Even though the patient is acidotic, why am I **not** giving sodium bicarbonate?" Those are the kinds of **clinical judgment skills** that must be triggered through the **teaching methodology**. That's what I've noticed—among the few I've interacted with, that's where the real challenge lies.

Interviewer (I): Ehee! Thank you—we will take that into consideration. Now, as we continue with our conversation, from your position as a senior leader here, what **nursing and midwifery skills** are currently lacking in your institution?

Participant (P): Ah! Here I can say... yes. Maybe if we set aside general competencies—because general competencies are typically grouped into four main categories. We usually say that any graduate or professional working at any level must be an **expert in their area**. So, for instance, if someone is a **critical care nurse**—like I am—we expect them to be a **true expert** in critical care. They should know the **interventions**, the **skills**, everything. Here, for example, we're involved in

oncology nursing, and the expectation is that practitioners must be able to **demonstrate** these competencies. From our side, that doesn't seem to be a major issue. But when it comes to **leadership competence**—for example, if you assign a young person, maybe one of our TAs or even an assistant lecturer, to lead a unit—you see that **leadership skills are lacking**. They may not be able to **create a shared vision**, or **develop a strategic plan**, or even **communicate and collaborate effectively**. So yes, leadership skills are something we clearly notice as a **challenge**, even among those we've worked with. Also, in terms of **research**, I do see that many—even the junior staff—are trying. I see them writing **grant proposals** and such. In **teaching**, of course, they're trying. But I still think there are challenges, especially in **clinical teaching**. As nurses, we benchmark everything against **clinical competence**. But most people have now opted for what we call **linguistic methods**—mainly lectures. And yet, when it comes to clinical teaching, the **educator must demonstrate**. If they're in the ward and they want to teach something like a **pelvic exam (PV)**, they must **demonstrate it themselves** and also **know how to teach it effectively in that clinical setting**. That's another area where we're seeing a gap.

Interviewer (I): Thank you. Now, let's return to your **area of specialization**. In your opinion, what **Master's-level programs** in nursing and midwifery should be introduced—and why?

Participant (P): Ah! I think right now we're looking at **demand-driven priorities**. **Maternal and child health** is still very much an agenda. Yes, we've seen some recent improvements—we've managed to reduce maternal and neonatal deaths a little—but we still **need more midwives**, especially **skilled midwives**. So, **Master's programs in Midwifery** are still necessary. The same goes for **Pediatrics**, because it's a field that has been **neglected for a long time**. We focused more on women and forgot the **children and newborns**. So, for me, **Pediatric Nursing** and **Neonatal Nursing** are areas we should definitely pursue. Also, the rise in **non-communicable diseases (NCDs)** has created demand for fields like **Nephrology Nursing**. This is especially relevant now that many dialysis units are opening, but the **big question** has become: **Where will we train these people** to gain true competence? So those are the areas I feel are **urgently needed**.

Interviewer (I): Now, finally, just to close our conversation—where do you see **gaps among specialized nursing and midwifery professionals**?

Participant (P): Mmh!

I: Eeh! In terms of current and future needs—do they lack any specific skills or areas of practice?

P: Yes! I think we still have gaps, even at the **Master's level**. We expect a **high level of mastery**. For example, if someone is a midwife with a Master's degree, they should know how to **handle obstetric emergencies** like they're second nature. If a woman presents with **obstructed labor** and needs assistance via **vacuum delivery**, they should be able to **manipulate and perform the intervention** confidently. But we've seen that this is still a challenge.

That might be partly due to **time constraints**. There's an ongoing argument—even here—that **two years may be too short** for true Mastery in such programs. The student is expected to **master an entire area of specialization**, complete a **dissertation**, and attend some **basic science courses** all within those two years. So, yes—**time is a factor**. But also, there's the matter of **supervision**

commitment, and once again, **clinical teaching**. We keep returning to that. So, I believe that if there’s a plan to **introduce such Master’s programs**, those are the **key areas that must be addressed**: Ensuring we have **expert instructors** with real field experience, And making sure they are available to **supervise students in clinical settings**.

Interviewer (I): Okay! Let me thank you very, very much for taking the time out of your extremely busy schedule. We sincerely appreciate it—and this marks the **end of our discussion**.

Participant (P): Aise, thank you—thank you very much.

KII Educators #2

Interviewer: Okay, thank you. Now, we would like to hear your opinion on the **quality of the Bachelor of Science in Nursing training for graduates**. How would you describe the **quality of these students** who have completed their undergraduate nursing education?

Respondent: Alright… aaahhhh, for me, the whole issue of quality—it's what you might call a **cross-cutting agenda**, right? You can't really say, "This is 100% top quality, this is the final definition." Quality is something that is always **evolving**—you keep aspiring for more. You reach a certain level, and then aim even higher. But overall, I can say that, **from what I see**, there's been a **very significant improvement** in the **overall quality of nursing education**—and that's due to several factors. For example, in our context, **curricula have improved**, we're hiring **more faculty**, there's **greater access to information**, and institutions are beginning to **better understand the nursing program**—unlike before. In the past—even at institutions like (he names a university)—people didn't really understand nursing… the program wasn't well-known or respected. But now, it's beginning to take proper shape. The **Bachelor of Science in Nursing program** is now developing more structure; people are starting to understand what it is, unlike before when students just studied without even knowing where the program was taking them. Now we even have **Master's programs** coming up, which offer a clearer picture of **specialization**. This helps even **undergraduate students** to see that there's **more beyond** just being a general nurse. Previously, everyone was treated the same—once you graduated, you were just a nurse, and wherever you ended up working, that was that. You might find yourself in any clinic, just by chance. But now, at least, some people are pursuing **specialization tracks**, and you can begin to see the **identity of the RN (Registered Nurse)** emerging. So I think the **quality is improving**. Even our **regulatory bodies**—back in the day, if you sat in a TNMC (Tanzania Nursing and Midwifery Council) meeting, there was total confusion. They didn't really understand what a **Bachelor of Science in Nursing** was or where it was going. But now, you can see even the **graduates from this program are involved in regulation**, and they are helping **define and oversee quality**. So for me, I really believe we've made great progress. Also, the **quality of students we're admitting** has changed. We're moving away from the old mindset that **nursing was for those who failed elsewhere**. Now, we're at a point where, to join a nursing program, you really have to **perform well academically**. In fact, these days, **after medicine**, nursing may be the program that takes in the **next highest-performing students**. That means if the **input is strong**, the **output** is also likely to be good—provided the **training processes** are in place. And like I said, I can't speak for all schools, but in ours, **sufficient resources are allocated**. In terms of **budget**, we receive adequate funding. Whether it's **recruiting clinical sites**, or **paying clinical instructors**, we have support. Now, when you go to a hospital, you'll find that **clinical instructors already have degrees**. This is a big change compared to the past—when you, as a Bachelor's student, were being trained by someone with only a **certificate qualification**. That was very demoralizing—and honestly, you didn't learn much from it. So across various dimensions, I can confidently say that **quality is headed in the right direction**—we're on a good path. When it comes to **PhD-level faculty**, back in the day maybe there were just two at MUHAS. But now, we have **PhD holders all over**, and they're **conducting research**. So yes, if you ask me about quality—I see a significant improvement. But of course, we'll continue to **strive for even greater quality**. That's just how it is.

Interviewer: Okay, thank you. Now, can you share your thoughts on **recommending Bachelor of Nursing graduates** for **further professional development**?

Respondent: You mean recommending them for… what?

Interviewer: Aah… when our graduates finish, do you think they are **really ready to pursue Master's programs** and such? What do you think about their **competencies**—those specific competencies?

Respondent: Aaahmm, I think… let me just say this—of course, people are coming in **with competencies**. But you can't really say it's the best across the board, because there is **a lot of variation** among the students we admit. And unfortunately, we don't conduct interviews. So, in the classroom, you don't see uniformity. When you say someone is an RN (Registered Nurse) in Tanzania, you'd **expect a certain level of competency**, but you'll find one person is **very strong**, and another is **extremely poor**, to the point where you wonder, "Where did this person even graduate from?" Sometimes they're both from the **same institution**, but they seem like they're from **two different worlds**. So that's a challenge. However, overall, we are starting to see an **improvement in the quality of graduates**, even when they get into Master's programs. There is one major issue though—it's a **national problem in Tanzania**—and that is **English proficiency**. You may find people who are competent in practical areas but when it comes to **writing**, maybe only **half the class is ready**, and for the rest, we have to start teaching **grammar and basic writing skills**. But when we compare our students here—since we're in a **major university**—others even say that our students are **slightly better off**. I think this is because during their **bachelor's preparation**, those who study **medicine or nursing** tend to be more **serious** compared to those in some other disciplines. So by the time they come to pursue a Master's, they are doing so because they **genuinely want to advance their skills**, not just to earn another certificate or look for job flexibility. So yes, we do get students who, even if they **lack some competencies**, they come in with a **genuine desire to learn**. And when someone comes in with that mindset—they learn, and they leave as competent professionals. Yeah.

Interviewer: Okay… okay, thank you. Now, can you talk about the relationship between the **undergraduate nursing program** and the graduate's **professional capabilities**, particularly in the following areas: **communication skills**, **human relation skills**, and **problem-solving skills**? Do you see a relationship between what they learned in the program and how they perform in real situations?

Respondent: So far, I can't really speak for people I haven't worked with—but if I look at the **students and staff we've hired here**, of course, in the university, we admit the best students. They are **very good**. When I look at our current team—the young people we've hired, or even just our fourth-year students—if I compare them to **our own cohort back then**, I'd say **they are much better**. When it comes to **communication**, they are very articulate, very confident. Even in class now, you'll see how confident they are when speaking. And I think this is also because we **admit top-performing students**. For example, over the past five years, all the students we've admitted were **Division One**. So these are **serious people**—they have a **strong academic background**. And I think the **nursing program itself also contributes**, because as you know, the nature of nursing really emphasizes these competencies—especially **communication skills**. That part is

well-integrated in the curriculum. As for **problem-solving**, you cannot finish a nursing program **without learning problem-solving**. Just sitting with a patient during assessment—you start problem-solving immediately afterward. Now, regarding the **third point**—**human relation skills**—that might be where we still see some **challenges**. This is mainly because we don't have **interprofessional teaching**. We teach people in **silos**. You'll find there is **no shared curriculum**, except maybe in Development Studies (DS), and even there, you study only with medical students. So, a graduate finishes the program without ever having **interacted with students from other disciplines**—like **business, law, or engineering**. But real life isn't like that. When you're put in charge of a ward, you'll have to work with **engineers**, **estate managers**, **procurement officers**, **human resource personnel**, and **administrators**. So yes, there is a gap. We don't have a space that allows students to **interact with peers from other professions**, and this makes it difficult to **develop that last key competency**—**human relations**—because we kind of **lock them in**, and their focus remains **narrowly on hospitals and patients**. But once they finish and enter the real world—they are expected to adjust and start interacting across disciplines. So yeah, that's where there's a bit of a **challenge**, and I think it's something we **really need to address in our curriculum**. I'm not even sure whether our own curriculum here has **looked at this issue deeply**. Yeah…

Here is the **full English translation** of the final excerpt you provided. It addresses key insights around **research skills**, **professionalism**, **practical training**, and the role of **teaching and learning preparation** for nursing graduates.

Interviewer: Okay, aahhh—and what about **research skills**, **professionalism**, and **practical skills**?

Respondent: I think for us, when it comes to **research** and **professionalism**, our students are generally **better prepared than others**. Honestly, I wish I could do a study—something to explore issues like what people often complain about: **poor customer care**, or the use of **inappropriate language** by nurses. I wish there was a way to **research that**, to see whether a person who has completed a **Bachelor of Science in Nursing** is among those who contribute to this negative image of nurses—as people who are **rude**, who use **harsh language**, and so on. But from what I've observed, when it comes to **professionalism**, I think these students are **well-prepared**—unless it's just **someone's personal character**, because, you know, some people are just naturally... hmm... prone to disrupt things. Even if someone attends church daily, if that's who they are—they'll still cause problems. So some cases may be like that. But in general, I believe the **curriculum does a good job** preparing students for professionalism. All the subjects we offer—like I've said—are well structured to ensure students are well prepared. But the **curriculum should also be reviewed** to strengthen those areas. It shouldn't just be about teaching theories. We need to focus on **real-life situations** from the profession—situations that really **require a nurse to act appropriately under pressure**. Right now, we teach too much **theory in ethics and professionalism**. It's slide after slide, without **bringing real-life examples** that would allow learners to **reflect critically** and grow—and for us to help them **model professional behavior**. On the **research side**, I think **nursing is doing really well**—in fact, even **better than medicine** in some cases. That's partly because the **nursing curriculum emphasizes research**. At least that's what I've observed. But another issue is how we **expose students to research** after they graduate. For example, during internship, there's **no research placement**. There's also no placement in

health systems management, which is where most big decisions are made. There's also **no placement in research institutions**. Everything is locked into hospital settings. But in reality, **not every graduate will go on to become a clinical nurse**. So I think we need to **reconsider** this structure. They tried to include something like **educational teaching** in the past—but I think a **nurse is also a researcher**. A nurse should be able to go into a science lab and actually **do scientific research**—even in **basic sciences or advanced science**. Because what nurses study is broad—they shouldn't be **confined to a small corner of practice**. We need nurses who can specialize—**nurses who are microbiologists**, who can go on to become **professors in microbiology, physiology**, or those who can make **inventions**. So internship and teaching placements **shouldn't restrict students**. They should provide **broad exposure to research**. These days, people talk about **nursing research**. But if you compare what medical students, public health students, and nursing students are doing—you'll see that nursing students are often **locked into one narrow area of research**. Their focus is just on **compassion, attitude**, and such topics. But they've also studied **microbiology**, and many other things. We need to **expand the scope of research** for nursing students—and let them know that **research is a critical component** of the profession and their future careers. So that's what I think.

Interviewer: Okay—and what about the **teaching and learning** component for these graduates? Can you speak to that?

Respondent: Yeah, for us—I think even recently, when we were reviewing our curriculum—this concern came up: "Are we truly **qualified to teach teaching**?" We teach **educational psychology**, **curriculum development**, maybe **teaching methods**—but I think there are **better-qualified people** to teach those areas. Even the **content** of the course shouldn't be dictated by us. Because what happens is "**garbage in, garbage out**." I was taught by someone who **wasn't a trained educator**, and the curriculum just said something like "nursing education" or whatever... We really need to **work with education specialists**. The curriculum should be designed to **actually train someone to teach**. If it's about curriculum development, then let it be taught properly. That's what we're doing now—we'll **partner** on teaching that course, but the ones who should teach it are **faculty from the College of Education**. They should own the content, because they are the **center of excellence** for that. Otherwise, if I say I'm teaching "teaching," I'd just be **misleading the students**—because that's **not my field**. I haven't studied it. What I can do is **add value** by sharing my **clinical experience**, or reflecting on how we teach in the clinical setting. But the **instructor for that course should be from education**. Yeah, I think that's a very good observation—and you (the research team) should consider that. There are many qualified educators out there. Go to **UDSM** (University of Dar es Salaam), bring them in to enrich the curriculum and teach the course properly—so that our graduates leave **truly prepared to be nurse educators**. Mmh.

Interviewer: Okay, and what about the BScN graduates—what **competencies do you think they're lacking** in the current program that you would like the curriculum review to address?

Respondent: Alright. One issue is… yeah… mmhh… **technology**—especially **emerging technologies**. There are certain areas… because we recently revised our own curriculum and addressed these, so I'll use them as examples.

We looked at **Health Information Science**, **Health Informatics**, and even **Data Analytics**—this whole component is **crucial** and should be included in nursing training. The world is moving toward **artificial intelligence**, **data production**, and **data-driven learning**. So innovation and data use are where we're headed. But nurses shouldn't just be **data generators** or **system input users**—they should also be **system users** who understand how to **extract insights** from data. For example, if I want to know why many patients are dying from a certain illness, I should be able to go into the hospital system or across multiple hospitals, **pull specific datasets**, run analyses, and interpret the results. A nurse shouldn't wait for someone to hand them a graph at the end of the month and say, "Look, death rates increased." They should be part of the **data generation and analysis** process. This area could include **two or more courses**—we've created at least two ourselves—to **orient students to digital systems**. The **second issue** is leadership and management. We teach a course in that, but when students go for hospital placements, they're often sent to **shadow a nurse in charge of a ward**. But the nurse in charge isn't a true **health leader**. If we want to train for leadership, we should **expose students to the wider health system**—where real decisions are made. They should attend **district and regional health meetings**, gain **residency at the Ministry of Health**, and participate in **national programs**—from **policy development** to **implementation**. That's the full leadership journey. So the **Nursing Leadership and Management** course needs to be broken down. Separate **Health Systems Management** from **Nursing/Hospital Management**. Have tracks for **Public Health Management**, **Hospital Management**, and **Health Systems**—and ensure there are real **placements** for each area. Many graduates leave without understanding how the broader health system operates. They clash with unit heads, and maybe escalate issues to hospital directors, but they don't understand **where medicines come from**, how **procurement works**, or how to **plan resources**. Nurses get stuck thinking their job ends at the patient bedside—but **many roles are evolving**, and nurses need to **see the big picture**. We also considered **clinical education reform**. We revised our approach so that **basic sciences** are taught until **year two**, and **core nursing theory**—like midwifery, pediatrics, medical-surgical—is compressed into around **10 weeks** of intense theory in year three, first semester. From there, students move **directly into clinicals**, with no more classroom work—just **rotation-based clinical practice** until graduation. The rotations are **simultaneous and thematic**, to reflect **real hospital conditions**. There's no point in saying, "I'm studying surgical nursing now," then you go to the hospital and only find **medical patients**. It doesn't make sense. So now, we prepare them with **foundational knowledge** first, and then rotate them across wards **regardless of specialty timing**, so they **apply all their knowledge** throughout. Class sizes are split into **tracks**—for example, a class of 80 is split into 4–5 tracks. In a pediatrics rotation, maybe only 14 students go in, and they're divided further by **shifts** (morning, afternoon, night) for more effective learning. We also teach **emergency and critical care** as **separate courses**, with **dedicated clinical rotations**. And for the future, I suggest you also look into **subspecialties** like **ophthalmology**, **ENT**, **dental**, etc.—even short **2-week rotations**—so the curriculum truly reflects the full scope of nursing. We've ignored these areas for too long. For benchmarking, we're happy to **share our semester structures**—semester one, semester two, second year—and you can adjust the content as needed. The goal is **harmonization**, not copying, just a **shared direction**.

Interviewer: Okay. And now in your department, what **nursing skills** do you think your faculty lack—and wish they had?

Respondent: Ahhh, quite a few. Are you referring to **faculty specifically**?

Interviewer: Yes—**faculty**.

Respondent: Ahhh, well… **we're a young team**, and we have to be honest about that. But we're growing **very fast**. I remember when I joined around **2014/2015**, there wasn't a single **nurse with a PhD** at the school. But now we're lucky to have about **20 people with PhDs**, and more are studying. Back then, we didn't even have Master's holders—maybe just one in **mental health**. But now we've started **Master's programs in pediatrics, midwifery**, and others, and they've produced **faculty with specialized expertise**—all of whom are now **registered for PhDs**. That's why we're now proud to say we can **run specialized clinical rotations**. For example, a **midwifery rotation** is led by about **five Master's-level faculty**, and some are now pursuing PhDs—so the **research component** is strengthening. It's the same in **pediatrics** and **medical-surgical**. We have trained people, but what **worries me most** is: The **number of faculty**, Their **maturity in research**, **consultancies**, and **grant writing**, and The ability to **mentor junior staff** and build **succession plans**. I'm also concerned about **exposure**. Many of our faculty trained **locally**, and they need opportunities to **go abroad**, see how others teach and research. So yes, those are some **gaps** we still have. But overall, compared to where we started—we're doing well. The team is growing, and the **motivation is high**. For example, young staff are already **writing grants**. Even at Master's level, they're mobilizing themselves, completing one or two grant cycles, and then moving on to more. So yes—**the momentum is building**.

Interviewer: Okay, and in these specializations, which **Master's-level nursing and midwifery programs** do you think should be introduced, and why? In your view, **which Master's programs** are needed and for what reasons?

Respondent: Aaahhh… now that's a bit of a challenge—personally, I find it difficult too. Why? Because we **need to sit down and define the progression** pathway for nurses moving into Master's programs. What exactly **is** a Master's degree in nursing? It's not always clear. For example, in **medicine**, you can't just say someone should pursue a Master's in **Oncology** without first doing **MMed in Internal Medicine**. You need that foundational specialty first to truly understand Oncology. But in **nursing**, we're jumping straight into **Master of Nursing in Oncology**, **Cardiovascular Nursing**, etc. When we launched programs like **Master's in Pediatric Nursing**, **Midwifery**, or **Women's Health**, the idea was that these would act as foundational pillars—similar to how a doctor would say, "I have an MMed in something," without implying their academic journey is over. From there, you'd proceed to **further specialization**. So rather than just filling the field with "Master's in everything," I believe we should consider creating something like a **Doctor of Nursing Practice (DNP)** or another **clinical doctoral route**. For example, within **Midwifery**, one could later specialize in **Reproductive Health** or even **Obstetrics and Gynecology sub-tracks**—but those should happen at **Doctoral or super specialty levels**, not all crammed into standard Master's programs. Otherwise, you end up with too many fragmented qualifications. Let's say I have a **Master's in Pediatric Nursing**, and someone else has a **Master's in Pediatric Oncology Nursing**—who leads whom? Who takes precedence? That's why we need to establish **command structures** and **professional hierarchy** within nursing. Without that, it becomes **noise**—you understand? We need clear tracks. Like someone first does a **Master's in Medical-Surgical Nursing**, and only after that, they proceed to

something like a **three-year Doctorate or super specialization in Oncology Nursing**. That way, when two professionals meet in the ward, the one with **general Master's training defers to the superspecialist**, just like in medicine. But now, you'll find a young graduate who just completed their four-year degree, goes straight into **Master's in Oncology**, finishes, and that's it. The next step is usually **PhD in research**, which doesn't always give them **clinical command** in a hospital setting. So, I don't know what approach you'll take, but my advice is: don't follow the route some universities have taken—where everything becomes a Master's, and **super specialties are treated like basic programs**. It really depends on how you choose to design the structure.

Interviewer: Okay. Finally, when you look at the **roles of nurses and midwives**, where do you see the **greatest gaps**, particularly with regard to **current and future needs**?

Respondent: Roles? What do you mean by roles?

Interviewer: Aahhh… things like patient care, research, teaching—those kinds of professional roles. Where do you see gaps, considering where we are and where we're heading?

Respondent: I think this goes back to what I was saying earlier. Those of us who've been **entrusted with authority**, we need to be very careful. Right now, I technically have the power to **propose any curriculum** I want. I could come in tomorrow and suggest a **Master's in Kidney Transplant Nursing** or **Bone Marrow Transplant Nursing**—and you'll find programs like these online already. The issue is that people are creating **Master's degrees around tiny specializations**. Right now, I'm curious to even look up a **Master's in Cardiology Nursing** and see: what exactly have these students mastered? You can't just create a curriculum with three cardiology-related courses, throw in some research and management, and call someone a **specialist**. So to me, the **real issue** is that we need to be extremely deliberate about how **specializations are defined**, because those are what eventually define the **roles** nurses take on. If we **rush into hyper-specialization**, we'll neglect building a **strong foundation**.

Interviewer: So, if I understand you correctly, you're saying we should **stick with core pillars**—like **Medical**, **Surgical**, **Midwifery**, and **Mental Health**—and only after completing those, a graduate should then specialize in something like **Oncology**?

Respondent: Yes—**exactly**. We need to **clearly define that path** and agree on it. Let's say someone completes a **two- or three-year Master's** in one of the **core pillars**—then they can proceed into **super specialization**. When we place them in hospitals, someone with a Master's in **Pediatrics**, for instance, can move into a **three-year Doctorate** in something like **Neonatology**. There, they'll do research and **gain deep expertise** in that field. For example, a **midwife** might go on to specialize in **Neonatal Health**. They'd then be a **Doctor of Nursing in Neonatology**, after **three years of clinical and research learning**. Their role will be very distinct from someone who only has a **Master's in Midwifery**. So when a **BScN nurse** encounters a **Resident Midwife** with a Master's, and a **Neonatal Nursing Doctorate holder**, their roles will be **clearly defined**. This hierarchy helps structure the profession. It lets the **Bachelor's graduate** understand their path forward and that their current role is that of a **general nurse practitioner**. They shouldn't feel entitled to move across all specialties without structured training. For example, they can't just start practicing in **Mental Health**—they must first do a **Master's in Mental Health**, and then move on

to define themselves within that specialty. Within **Mental Health**, super specialization could include areas like **Addictions**, **Psychotherapy**, or **Adolescent Psychiatry**. So I don't know what direction you'll take—but right now, I'm seeing people being labeled "**Advanced Practitioners**" across the board. But what does that even mean? These are things we **need to understand deeply**, and they are **very critical**. I don't know what platform we'll use, but at least **we, as internal experts**, have the power to **determine the way forward**.

Interviewer: Thank you very much for your time and your willingness to participate in this… aahhh… KII (Key Informant Interview).

Respondent: Yeah—this is very… it's a very important exercise. That's why we've dedicated sufficient time to it.

Policymaker #1

Interviewer: You, as one of the policy makers, I had a few questions which I would like your opinion on.
Respondent: Okay.
Interviewer: Okay. What are your thoughts for HKMU—can you give us your opinion regarding the training of the Bachelor of Nursing program?
Respondent: It should be four years.
Interviewer: Mmmhh, we should make it four years?
Respondent: Yes, four years.
Interviewer: Okay, and why do you think it's better if we make it four years?
Respondent: Making it four years is good because they will have time to study and learn more things. For example, if someone is doing biochemistry, they will be able to study its details, but also when entering into nursing issues, and also hands-on—because one can be good in theory but not good in practice. But with four years, I believe they will have time for both practical and theory.
Interviewer: Okay, so by making it four... do you have other comments?
Respondent: Other comments, apart from that—if I had seen the curriculum at least I could advise more, because the years might not be the issue, but we would be able to see the content—what is being taught. They can stay in class four years, right? But maybe they just sit there from morning to evening talking nonsense, then leave. If I had seen your curriculum, I could advise more.
Interviewer: Okay, and now, do you think there are specific competencies you would recommend this nursing program to give its students so that when they graduate they can cope with different health challenges happening within our country and even outside it?
Respondent: Honestly, I will speak from experience when receiving those who come with a Bachelor... those who come with a bachelor aaaahhh, to tell the truth, even in terms of decision-making, there is still a challenge—making quick decisions like this patient... because as a nurse, they should have a sharp mind, be keen-eyed, have seen something, made a decision, and implemented it. Honestly, I've seen this as a big challenge. I've seen it as a big challenge in all the students I've received. Mmmhh... even when you ask something, you see them hesitating—they are not sure that what they're about to answer is correct.
Interviewer: Okay, so for those challenges, what competencies do you think we should give them specifically to help them when they reach the workplace and begin dealing with those health challenges they encounter in our community?
Respondent: Mainly, even if they study the pathophysiology of a disease, they should study and understand it. When they study management of the disease, they should study and understand it. When they say, "this case is on me," they shouldn't just give up. Even if it's a referral, they should be able to decide in time and know "I've reached my limit; I can't go further." Yes. But also, you could add something like **critical thinking** as a proper course. I believe they would then be able to criticize some issues—not negatively, but constructively. Yes.
Interviewer: Mmmmh… okay. And how is this graduate of the Bachelor of Nursing recognized within the Tanzanian government employment structure?
Respondent: Repeat your question.
Interviewer: How is this BSc.N graduate recognized by Tanzania's public service structure? If they are recognized, how?

Respondent: Aaaah, they are recognized as a nurse who has a bachelor's degree in nursing and who can carry out responsibilities. Of course, in the scheme of service, their duties are also outlined. Yes, they are fully recognized. A bachelor's holder has no challenges.
Interviewer: Okay, so the scheme of service explains their roles?
Respondent: Yes… it explains their duties. First, qualifications—what qualifications should they have? They should have studied science subjects and be from a government-recognized institution, and they must have done internship, and that already qualifies them. And that's why when they come, they start at that level—even though the level is a bit low. We have… we are fighting… we've submitted proposals, and they've reached all levels so that they can start at a higher salary scale. Yes… so they are recognized.
Interviewer: Okay. And what are some national issues that you would recommend be added to the Bachelor of Nursing curriculum?
Interviewer: So I was asking, what national issues would you recommend we add to the BSc.N curriculum?
Respondent: Monica… I'm afraid to go straight because I haven't seen it—if you had shared it with me…
Interviewer: Ahh… just in general. In general—not specifically for Kairuki…
Respondent: Ah, for… for… everyone?
Interviewer: Yes… for all.
Respondent: Of course, I would have wished for you to add **M&E issues** (Monitoring & Evaluation) in it—I don't think they study it, honestly. Mmmhh… M&E issues, you should add them. We struggle with them a bit when they arrive—even if you tell someone to do monitoring and evaluation, first they just stare at you. If time allows, let them study M&E.
Interviewer: Okay. How do you think it can help them when they come to the workplace, if they take an M&E course?
Respondent: If they study M&E—because maybe they already have statistics on something—you know, even doing **operational research**, these students can't do it. You know that? They can't.
Even just a simple operational research—you tell them "do it in your ward, publish it, there's a place you can publish,"—they can't. So they will be able to process that data—"I have these patients, I see these diseases are leading, I can do research on this and explain it," etc. That will help them.
Interviewer: Okay, eeehhhee. Any other issue you would like added in this BSc.N curriculum?
Respondent: Are you including leadership? Is it there?
Interviewer: Aaanhaaa… leadership… eeehhh?
Respondent: Leadership and management. Yes. Because these are the people who will later become leaders here.
Interviewer: Okay, fine. So that's for the nurse at the bachelor's level. Now for the nurse at the master's level—what issues would be good to include in their curriculum?
Respondent: In the?
Interviewer: In the **Master's curriculum** for nursing and midwifery—what national issues would you recommend we add when preparing these programs?
Respondent: I will continue to insist on **leadership and management**, **M&E**, and **critical thinking**. I see those things… because once someone can… so I think those will help them even in making decisions, at any level they'll be at. Yes.
Even if they're studying midwifery, it doesn't mean they'll only stay in the ward. There may be

leadership roles. They'll have to do evaluation, follow-up on midwifery services in the ward they're working in and see what's going on.
Why is maternal mortality high? Isn't that right? They can help us. Maybe **perinatal death** in the ward is rising, etc.
Interviewer: Okay, now when it comes to establishing these Master's programs in nursing and midwifery, what things support and what things hinder their establishment? Let's start with things that support.
Respondent: It's having… first, having instructors—I see teachers are very important.
Eeehhh… once you have enough teachers, and students with the criteria…
But also, practical training—because things like midwifery—it's more practical. Where will they do it? Eeehhh… because theory alone, they can't be a good midwife. They must do hands-on to become a good midwife. So the key is teachers, a **conducive learning environment**, and places for practicals—which at Kairuki I'm not worried about, since you have your own hospital, right?
Interviewer: Yesss… we do.
Respondent: So have enough teachers and a curriculum that follows the country's guidelines—so that when we say Ziada is a midwife, we really mean Ziada is a well-trained midwife—not someone who just touches things.
Also start **Geriatrics**—you know we don't have geriatrics.
Interviewer: Okay—geriatrics?
Respondent: Yes. We completely don't have any geriatric nurses. You won't believe it. Right now, we have a **geriatric unit** that's been opened… mmmm… but we lack even one geriatric nurse.
Interviewer: Okay. Now we've talked about things that support starting these programs—you mentioned teachers, students with criteria, enabling environment, and practical areas. Now what do you think are things that might hinder their establishment?
Respondent: Mmmmhhhhh… what could hinder is—if you don't meet the criteria, you won't be allowed to run the program. Yes, that's the main thing. Otherwise, we need to have many specialists—because now we're looking for people with PhDs, because as we go on, we're starting to phase out gradually. For example, the **nursing certificate**—it's slowly being phased out, so eventually, it won't be there. Yes.
Interviewer: Okay, and now how is a specialized nurse or midwife with a Master's and beyond recognized by Tanzania's public service structure? How does the scheme of service recognize a specialized nurse or midwife?
Respondent: Honestly, in the current curriculum, they're not recognized.
But the one we proposed does recognize up to a **nurse consultant**—that is, with a PhD—which we expect to pass this year. But we do recognize their contribution in the workplace—though in the scheme of service, to tell the truth, we only have three levels: certificate, diploma, and bachelor.
Eeehhh… but this scheme is very old—over ten years old. Now it's been reviewed again and passed through all councils—we expect it to pass this year.
Interviewer: Okay. Now for these Master's programs that have already started in different universities—nursing and midwifery—what things do you think can support their continuation and what might hinder them?
Respondent: First, when they start offering those Master's that are **nursing-related**—because some people say they are offering a Master's, but it's actually **MPH**.
And when they do MPH, they can't apply it to nursing. So they go against what they agreed with

the ministry and government.
Yes. But also, others say their Master’s is two or three years—but you find someone finishes in 18 months. See? Now, those 18 months—you can’t say that equals the expected years. That becomes a challenge.Also, **content matters**—because in your curriculum you said you’ll teach this, but what you’re teaching doesn’t match—and you produce students who are not competent, as expected. So your school will be stopped from offering those programs. Yes.
Interviewer: Okay. Well, I would like to thank you for your time. Thank you for agreeing to participate in this discussion.
Respondent: Thank you so much.
Interviewer: Thank you very much.
Respondent: Are you starting **psychiatric**?
Interviewer: Psychiatry..? We are in the process of starting.
Respondent: Ahhh… okay, start it! I believe that scheme of service will pass. So encourage many nurses—I also keep encouraging them—and the government is trying, you know, even **sponsorship** is increasing for nurses going for Master’s.
Interviewer: Okay, fine.
Respondent: Alright, thank you.
Interviewer: Okay, thank you so much.

Policymaker #2

Interviewer: So, you as one of the stakeholders, what recommendations do you have for Kairuki University regarding the training of undergraduate nursing students? What would you recommend?

Respondent: Undergraduate, postgraduate…

Interviewer: Undergraduate.

Respondent: What's important is that... because I haven't specifically had exposure with Kairuki students per se, but I can offer general opinions.

Interviewer: Okay.

Respondent: That universities should be able; first, to develop programs that will train students so that when one graduates at their level—if it's BSN—they graduate as a true BSN in the sense that they have enough competence for that level and can do what is expected. Unlike now, where many—although you can't always tell which university someone came from—many finish university with little competence compared to what's expected. We give them assigned duties, but you find that some perform in a way that makes you think a diploma holder might be better than a degree holder—which we see is not the reason. Our expectation is that this degree holder has studied more, understands more, and should do greater things which will ultimately help improve patient outcomes and service quality. Also, they should be good leaders who can coordinate their performance, supervise service delivery, and provide care themselves as a good example. So, all these things should be ensured by the institution offering training—ensuring that students are supervised and the curriculum is well designed to match actual needs. When they graduate, they should be able to work independently without worry.

Interviewer: What specific competencies would you recommend in this undergraduate nursing program that would enable a graduate to address various emerging health challenges in our country and even globally?

Respondent: Even when you look at graduates today, mostly—except for those with master's degrees—at some hospitals lucky enough to have them, you find those specialists are the ones with higher education. So mostly, we depend on them to be good leaders. So, **leadership skills** are very important. We expect them to be leading persons—even though we call them junior researchers—they should be able to do **operational research**. You'll find many have worked for 10 years but have never done a single research project, not even as a research assistant—maybe just collecting data for someone else. So we should expect that, since they're taught, they should be able to conduct research—especially to inform challenges. Just collecting a few data points might help us, the Ministry, to make informed decisions. But now, such research isn't done and workplaces suffer.

Also, we expect these people to provide patient care that shows a difference. I want to see a patient cared for by someone with a BSN and that the patient is satisfied. **Client satisfaction** comes when a nurse has strong **customer care** skills—something often ignored in training—and also practical skills in their area. If it's pediatrics, this nurse should deliver comprehensive care to the child, not just wait for instructions from, say, a doctor. They must truly act.

The **nursing process** has become a major challenge—we expected BSN graduates to help with this area, but they themselves still need training on it after employment, which is problematic. The nursing process is the **core** of nursing practice—if they can't apply it, that's a huge problem.

Interviewer: Okay. Now, how is the nursing graduate recognized by the Tanzanian public service scheme?

Respondent: The structure is there—they are recognized. Upon entry, there's an entry point and rank, TGSH C. But the challenge is that, compared to other cadres, it is still low. The government is in the process of finalizing a new structure that—compared with other cadres—should improve things, especially in terms of **motivation** and **incentive packages**, like salaries. That's the only current issue—the structure is low—but at least it exists, unlike in the past when we started and there was no structure at all.

Interviewer: Okay [laughter]. So, what specific national issues would you recommend be added to the undergraduate nursing curriculum?

Respondent: Mmm, if I had seen what you teach, I could tell you exactly what should be added. Right now, many things are being taught that require focused training. For example, we now have **intensive care** issues. Yes, we need **critical care nurses**, but currently, critical care is still very general—it hasn't been made specific, which it should be for someone doing a master's degree. A master's student should be very specific, but currently, critical care is defined as any patient in critical condition. But we need specialization—renal, cardiac, ICU as a unit, emergency care—these areas overlap and need to be more defined even for BSN students.

Also, I think it would be important to equip them well for the **ICU**, because even when we studied, we only passed through it by chance—we never got intensive training. Then there are areas emerging like **forensic** and **psychiatric** nursing. We still don't have national-level experts in these fields. Even in psychiatry, BSN graduates need better training so that—even if they don't specialize—they can still help meaningfully.

There are areas like **burn care**. We don't have specialists there, yet it's needed. In some countries, people even get PhDs in wound care. We also need to address the rise in **technologies**—lots of new machines are being used, and nurses are with the patient 24/7. If they don't know how to use them properly, they may end up harming patients without knowing it.

Interviewer: Okay, now shifting to the master's level, what national issues should be integrated into the curriculum for nursing and midwifery master's programs?

Respondent: I think I've already mentioned some areas that should be considered. But if we go back, some programs that were once specialized have now been phased out, so we lack staff in those areas. Even **nurse educators**—we have no school producing them. A nurse needs to first become a nurse and then be trained as a health educator. A **nurse tutor** must have dual qualifications—nursing and teaching in health sciences.

Also, emerging issues like **cancer care**—a few universities have started **oncology nursing**, and oncology itself has branches that need attention. Eye care—we don't have nurses specialized in

ophthalmology. ICG services? We don't have them. Pediatric nursing—very few are trained, and we need to expand that, including **neonatology**.

We also lack **elderly care**, though we have many elderly people. **Counseling** used to be a strong area for nurses but is now neglected. Things like **nutrition** are being pushed to other cadres, though it originally came from nursing.

There are areas like **cardiology**, **nephrology**, **theater management**, **health economics**, **decision-making**, **leadership**, and even the rare but important idea of having **nurse politicians**—who can take the nursing agenda to Parliament. That's where key decisions are made that affect nursing.

Also, in **midwifery**, we need **specialized midwives**—someone focused solely on **labor**, or **antenatal care**, or **managing complications** like PPH. But currently, midwifery has become too generalized.

We need **health economists**, **evaluators**, and more **specialized services** in nursing to match specialized medical services. For example, **orthopedic nursing** is lacking—we don't have specialists in that.

Unlike other fields where one person can serve an entire hospital, in nursing we need **many** specialists in each area to serve effectively.

Interviewer: Thank you. What do you think supports or hinders the introduction of postgraduate training in nursing and midwifery?

Respondent: On the hindrance side—there are many things. It's not always a direct obstacle, but sometimes it's **lack of motivation**—for example, the **scheme of service** doesn't recognize a specialist nurse. That's number one. Second, **sponsorship**—it's a problem. I try to push for it, and we get a few, but not enough.

Another issue—we haven't **created demand**. We assume even a certificate-level nurse can handle everything. That leads to **poor health outcomes**. Also, many haven't followed the right academic progression—some only pursued BSN after many years. When it comes to master's entry, many don't meet requirements or feel too old.

Then there's a **shortage of institutions** offering relevant master's programs—and limited space even where they exist. Most focus only on **mental health** or **midwifery**—maybe a bit of pediatrics—but we need **more programs** aligned to the services we need. Also, more **graduates per program**.

Some specialties like **wound care** are being ignored, yet in other countries, they go up to PhD just for wound care.

There are also **policy analysis** and **policy implication** skills needed. So, many issues discourage people—but also, some **encouraging factors** exist.

However, in hospitals, there's often a **tug of war**—"Why should I let you go study and return to compete with me?" [laughter]—we discourage this mindset, but it happens.

Other barriers include **lack of awareness**—some people don't even know master's programs exist. With more **sensitization**, and when **scholarships** become more available, many will enroll.

I've encouraged many—from diploma to degree to master's—they made it.

Interviewer: How is a specialist nurse or midwife recognized by Tanzania's public service structure [laughter]?

Respondent: I think I mentioned—the current structure doesn't really recognize them. But in terms of **responsibilities**, yes—they are assigned advanced duties. Even the nursing council licenses them according to their **scope of practice**. What's missing is **an official salary structure**, which is supposed to be in the **new proposed scheme**, recognizing up to the PhD level.

Interviewer: So for the bachelor level, you reviewed the scheme…?

Respondent: The scheme is one—it covers all nursing cadres.

Interviewer: Okay, so for the master's holder, their roles are…

Respondent: They are identified.

Interviewer: But the salary isn't…

Respondent: The current structure doesn't explicitly list master's holders. It just shows a **career ladder**—from degree level onward—but doesn't separate master's holders like in other fields. That's what's addressed in the proposed new structure.

Interviewer: Okay. This relates to the above—regarding existing postgraduate midwifery programs, what factors support or hinder their continuation?

Respondent: It depends on the institution. People are needed—so the institution must do **sensitization** so that people know opportunities exist.

From the government side, **more scholarships** are needed. The main challenge is **tuition**—students are accepted, but paying fees is hard because salaries are low.

Also, **infrastructure**—if institutions provide a wider range of program options, that can be motivating.

Interviewer: Alright. I'd like to thank you for your time and for agreeing to this interview. Thank you so much. [laughter]

Respondent: Thank you. One last thing I forgot—**family health**—it also needs to be covered well under specialization.

Interviewer: Okay, family health?

Respondent: Yes. Someone can **specialize** in family care. There's also **community nursing**—these are important areas not to forget. Thank you for coming. Sorry for keeping you.

Interviewer: No problem.

Respondent: Alright.

Interviewer: Thank you.

Policymaker #3

I: So, welcome to this interview.

R: Thank you.

I: As we go to review our Bachelor of Science in Nursing curriculum, as a stakeholder we would like to interview you about various issues to help us as we review our curriculum.

R: Okay.

I: What recommendations do you have regarding the training of undergraduate nursing students?

R: First of all, I'm happy and thankful to be here for the interview because it is my desire to get nurses with skills. First, my wish is to get nurses who can perform—that is, work with patients. Looking at our nursing history, in the past we were taught as if we were going to be leaders, so you find our training didn't focus much on practical training. So I would like the new curriculum to prioritize extensive practical training time. Also, investing in having enough practical instructors, including classroom teachers making the effort to reach the wards and teach practically. That way, we will get a degree-holding nurse who can work directly with patients, considering today's job market and government trend. We need degree-holding nurses even in council hospitals and health centers. So this will help us get nurses who will assist us effectively at work.

I: Thank you. What specific **competencies** would you recommend for this undergraduate nursing program that would enable the graduate to handle health challenges emerging in our country and even abroad if they work there?

R: Hmm! First area is competencies in providing services to a pregnant woman during delivery and the 42 days after giving birth. They must have enough skills in that area, including providing emergency services for mothers, known as Basic Emergency Obstetric and Newborn Care skills. That's the first. Secondly, as you can see, the world is now faced with outbreak diseases, so I also wish to see how care can be provided in the context of outbreak diseases. First, to have competency in emergency care, but also in critical care—those are essential areas. A major one I also think is important is for students to be taught about **customer care**. The health service delivery system globally and even in Tanzania is now shifting—from institutions or hospitals giving instructions to patients—to more involvement of the patient in service delivery and being able to meet patients' expectations. So to align with this, our students must study customer care.

It's also very important to see how we can place **non-communicable diseases** properly. I know we used to study hypertension and diabetes, but in today's world, the disease dynamic is moving from infectious diseases to non-communicable diseases due to lifestyle and other global factors. So I'd like to see more emphasis and energy placed on modules and subjects related to non-communicable diseases.

I: Thank you. How is the **graduate nurse** recognized in the Tanzanian public service structure [scheme of service]?

R: Thank you. The Bachelor of Nursing started being recognized in the public service scheme from the year 2009, where the graduate was recognized as a health cadre—meaning nursing cadre. But there were challenges in terms of salaries and salary scales, which were set lower compared to various employment criteria—first, the years spent in school, and also the working environment and the importance of the work they do. Because we know that nursing and midwifery, if not present, services cause direct consequences such as death or disability. So due to the weight of their work, along with the education they received and time spent in school, the scheme of service needs to be improved. And already, as the government, we have worked on improvements. We have submitted the draft of the new scheme of service to the President's Office for final review. And God willing, our government might begin to implement the new scheme which will have better benefits.

I: Thank you. What specific national issues would you recommend be added to the undergraduate nursing curriculum?

R: As I mentioned earlier, one very important issue is **customer care**. This issue is especially important now as we aim to improve health services. In improving health services, we emphasize service quality. One part of service quality is customer care. Nurses are often criticized for poor communication and other negative behaviors toward patients. That area must be emphasized so that a nurse graduating from school can treat patients while observing **dignity** and **human rights**, and all issues related to **ethics**—professional ethics, national ethics, and public service ethics. That's the first area. Second, as I mentioned, as a country, we still have a major challenge with **maternal mortality**, so that area must be properly addressed. And we say in Tanzania a nurse is also a midwife. So they must be able to provide **midwifery services** according to national guidelines. When developing curricula, review the various national guidelines and see what midwifery services are required and include those competencies thoroughly.

The third and final point is the area of **communicable diseases**. As I said, it also needs to be emphasized. As a country, those are the important areas we see—along with outbreak diseases. Train the nurse to also understand the **preventive aspect**—basic **epidemiology concepts**—to be able to address outbreaks from **prevention to curative** stages.

I: Still on that, what about postgraduate degrees in nursing and midwifery? Are the issues the same or can you add a bit since we're at a different level?

R: Yes, I can add. At the undergraduate level, we're building the foundation, so the person is a **generalist**—you can place them in any area, and they will work. They have the basics as I previously outlined. But as we go to the specialization level, that's at the **master's level**. At the master's level, we strongly encourage **nursing master's programs**. For a long time, we had very few master's programs in nursing. And even now, although we have added some, they are still few. We encourage many **specialty programs**, whether in NCDs, neurology, pediatrics, maternity, emergency, critical care—there are so many areas. As a country, we haven't established many specialties, but the market for specialties is there. As I mentioned regarding the scheme of service, we're not only improving the benefits of bachelor holders, but we've also recognized **masters holders**, and current national policy wants **specialized services** to begin at regional hospitals. We now have 26 regions and many government hospitals—28—and private

hospitals at that level and above. So we really need to **expand training programs** based on our sectoral needs and priorities.

I: Okay. And in your view, what supports or hinders the **establishment** of master's-level nursing and midwifery programs?

R: There are two areas. First, **policy support**—you need to consider what postgraduate priority areas are identified by the Ministry of Health. These determine job market demand. Other important things include the **quality of the institution** offering the postgraduate program. This is under authorities like TCU. You must have qualified faculty, and a **teaching hospital** that qualifies to teach those areas. But most importantly, it's about **policy needs and priorities** in those specialties. The professional council also looks at policy priorities.

For example, right now we have a shortage of **neonatal nurses**, **pediatric nurses**, and nurses for **non-communicable diseases**. The government has invested heavily in specialized services, equipment, etc. Oncology, for instance—cancer is increasing, but oncology nurse production is still low. Same with **cardiac diseases**, **renal failure**, **dialysis**—we have many diseases needing specialized care.

It's unfortunate when a doctor is specialized and works with a **generalist nurse**, sometimes with just a diploma. That mismatch becomes a challenge. Our Minister of Health directed that when supporting studies abroad, we send a **package**—both nurse and doctor to specialize in the same area. That way, they'll be able to collaborate well upon return.

Also, when such programs are aligned with national priorities, the **government can more easily fund** them. We fund students studying priority specialties—someone studying Public Health may not get sponsorship, but someone studying **nephrology** might, because we need them for dialysis patients. So **funding** is a critical factor when launching a master's program.

I: Okay. And how is a **specialist nurse or midwife** recognized in Tanzania's scheme of service?

R: Thank you. As I've said, when we develop structures, we look at the availability of that professional to the patient. In our **recently submitted structure**, we've ensured to recognize the **master's-level nurse**, but the type of master's we value is one that helps the nurse deliver care differently from a bachelor holder. So all specialist master's roles that I've mentioned have been recognized in the **new draft scheme**, which we hope will soon be implemented.

I: Now for the master's programs that have already been established—say, in **critical care**, **mental health**, etc.—what factors help or hinder the ongoing delivery of these programs?

R: Thank you. Let me begin with the factors that hinder.

I: Okay.

R: One is **motivation**—if someone graduates but their salary and incentives don't change, it discourages others. That's the issue if the **new scheme hasn't passed yet**. Second, **lack of scholarships**—say 50 students apply, but the budget allows sponsorship for only 20. The rest, who can't afford to self-fund, will be left out.

On the **positive side**, what promotes these programs is their **recognition in the scheme of service**—showing the person will get better salary/incentives. Also, **policy emphasis on specialization** encourages people to enroll. And naturally, **humans desire to grow professionally**—so some people, after working for a few years, feel they should return to school to enhance skills and knowledge. That also promotes participation.

I: Thank you very much. Do you have any final general thoughts, advice, or reflections from this interview?

R: I do. First, I'm really impressed and grateful you came to conduct a **tracer study** at the policy-making level. It's my first time being involved in something I've always dreamed of—it's important for improving our profession.

Secondly, we've faced a huge societal challenge where people say **degree-holding nurses are not hands-on**, that they just want management roles. Partly, I understand this perception—it was rare to find degree nurses in hospitals, and our profession advanced late. But to erase that image, we must **go out and work** in various areas and show we can deliver **different and better care**.

Let me repeat—when it comes to skill development, there's **skills, knowledge, and attitude**. The **skills part is still a major gap** for degree students. They're well taught in class, but there's weak connection between theory and practice. Doctors, for instance, have teachers who teach in class and also do ward rounds with students. They connect lessons to real patients.

In nursing, we lack that **link between school and teaching hospital**—I strongly urge you to address that in your curriculum. Also, the **teaching methodology** plays a big part. Are we teaching competencies through lectures only? We need to adopt **activity-based methods**: reflection, scenarios, case studies, small group discussions, demonstrations.

If it were up to me, I would drastically reduce lectures. Lectures should be **self-directed learning**. Students should read content before class and come for discussion, reflection, and hands-on activities. That would help students **internalize and own** their learning.

In the **curriculum development process**, it's crucial to involve **key stakeholders**—both technical and community. Include policy makers, trainers, service supervisors, and employers. Blend those voices for a **well-rounded and inclusive curriculum**. That way, you're more likely to create something truly useful.

I: Thank you very much. I appreciate your time and for agreeing to do this interview.

R: Thank you very much.

I: Alright.

Policymaker # 4

Interviewer: What recommendations do you have for Kairuki University regarding the training of Bachelor of Nursing students?

Respondent: My recommendation is that they reduce theory and increase practicals because, from my experience, many students graduate with a lot of theory but they don’t get enough weight in clinical practice. As a result, they lack proficiency—competences, skills, and even attitude. You know when you go and spend more time in clinical areas, you even shape your attitude. So my recommendation would be that theory should only cover the main areas, but most of the time should be for clinical practice.

Interviewer: Because in many curricula you find the time is squeezed, the contents are many. So to improve now, maybe you can advise us on what to reduce so we can increase clinical time by a large percentage.

Respondent: From my perspective, I would say in training, I would like first that the sciences be enough at the beginning—meaning anatomy should not be superficial, just name-naming. Also physiology, biochemistry, microbiology—the basic sciences should be sufficient. But when we come to nursing subjects, we could place more emphasis on the foundations of nursing. But also on how to provide care to patients with surgical problems—we call it surgical nursing; medical problems—we call it medical nursing. But we could also... there are these other subjects—how to provide care to children, and care for pregnant women and related matters—meaning reproductive and child health. But we could also emphasize ethics and customer care a lot. But these other subjects—those are the foundation, and these trainings should be more based in clinical areas. Because nowadays, many colleges say we are going to the skills lab—but actually, that doesn’t shape them. I have witnessed some students, when they go to the wards—especially during their internship period—they are afraid of patients. But if you ask them about theory, they speak well. But in hands-on? There is a problem. Even the attitude—how to approach—because they were practicing on dummies. You know, practicing on dummies is different from real-life situations. So that’s what my views would be.

Interviewer: Now, due to changes and increasing technology, many new problems arise. What skills would you suggest we add to our nursing curriculum to tackle national issues that arise?

Respondent: Mmmhh. First, I’d say now is the age of science and technology. Mmmhh. Because it is... the age of science and technology. Apart from having the manual skills, I’d like to advise that even this technology—using machines to know the patient's condition—we invest in technology. Because nowadays there are many machines. In the past, if you wanted to give medication, you’d use your eyes to observe. But nowadays, there’s an infusion pump. So this technology—we must invest in it. Also, in these competences, I’d say let’s invest more in the nurse’s assessment skills—being able to identify patient problems. And once they identify the problem, they should be able to act. So they must have cross-cutting competencies, according to our scope. But I’ve also mentioned scope. If it were up to me, I’d wish that colleges review this, because we’ve limited the scope of nurses too much. Because of that limitation, sometimes they assess well and identify the problem—but we limit them and say, "Wait for someone else to

prescribe." So if it were up to me, besides the usual things we know—giving medicine, catheterization, IV fluids—we would also give knowledge on how to **prescribe** at various levels: lower, mid, and upper. So that once this person finishes their degree, assesses the patient, knows the issue—but just says, “I’m not allowed.” I’d like colleges to open up and expand the scope for degree-level nurses. For example, someone looks and wants to give valium but wonders, “What’s this?” Just because of prescription issues. Let’s look at how to incorporate that knowledge. Because **prescription** is not just about saying “take two pills.” What is the **principle** of prescription? How far can they prescribe? So we can increase the period in which they can see a patient, assess them, come up with a diagnosis, and know that—at their level—they can treat the patient up to a certain point. That’s what I wish.

Interviewer: Now, since you’re a key stakeholder, and many times we hear people say, maybe we can even be taught how to read lab results, interpret them—but after interpreting them, if you’re on night shift and already know the issue, you still have to wait for the doctor to come and give the go-ahead. So as one of the policy-makers, how do you help nurses to be empowered? He’s already read and understood the issue—but waits until morning for a doctor to approve what could’ve been done during the night?

Respondent: It’s like this—we work in levels, as a team. You know, this degree-level nurse calls the on-duty doctor—maybe a specialist—it’s not a matter of just calling and saying, “Hello, this is Agnes, come see the patient.” That is not the communication expected at that level. The communication should be: “Hello, this is Agnes from Ward Two. The patient, so-and-so, has this problem. His lab results show high calcium. I’m concerned this might cause complications.” We don’t do that—we just say, “Hello, come see the patient, it’s gotten worse.” What is “worsened”? So here is where they need to be **empowered**—to be able to make decisions, to know the problem of the patient. This patient, after my assessment and tests, shows this problem. So now I’m calling you because—even if I had the power to initiate treatment—I’d still **discuss** it with my colleagues. You must get opinions from others. So I’m saying, add this. If they can interpret and know the dose, and know this patient is **dehydrated**, it’s not necessarily about giving antihypertensives—maybe just IV fluids because electrolyte results are off. Now this **Ringer Lactate** I’m giving will help him. That kind of reasoning—ehh, so it's not just, “I know now, so prescribe aspirin!” It’s interpretation and giving rationale. Even when I talk to them—say you’re in Dar es Salaam, and the doctor is in Bunju—you tell him, “Come see the patient.” That’s why sometimes they don’t come—they arrive not even knowing the patient’s issue. But I, who stay with them 24 hours, when I call and say, “This patient has 1, 2, 3... their results show X, Y, Z... probably they have this... and I don’t think they’ll make it until morning.” That kind of communication.

Interviewer: Okay! How are Bachelor of Nursing graduates recognized in the Tanzanian public service structure?

Respondent: Mmmh, so far, they are recognized up to **bachelor’s level**—though there are challenges. Challenges because even us, as training institutions, we’ve differed a lot. Some teach for 2 years, others for 3, others for 4. So when trying to recognize and allocate their benefits, the policymaker will use the **minimum**, and that minimum is 2 years—even though you teach 4 years to provide enough science for reasoning. So I’d say training institutions should **agree on a**

common standard—what's the ideal training period, and what content? They are recognized, yes—but at a **lower level** due to this confusion. Someone trains for 2 years—so why complicate things with 4 years? That's why they rank you at the 2-year level. Look at medical schools—it's 5 years, no debate. But for us, we argue too much. That's what leads to our people being recognized at low levels. Our **training standards** are what cause the problem.

Interviewer: But isn't the **Registrar** part of the regulatory bodies that authorize these colleges—like TCU? Don't they get authority from TNMC too?

Respondent: Yes, there's TCU and NACTE. For example, at NACTE, no college can currently offer 2- or 3-year training. But in the **TCU regulatory framework**, there was a lot of variation—and still, some colleges are struggling with this. But we at **TNMC** have already declared: Year 1, fill them with science. The other 3 years go into the profession. So from now, we will only recognize those from **4-year** programs. You know what's happening now—people are going abroad. Some programs are 2 years, others 1 year, others 3 years. That's a challenge we've seen. We've already spoken to TCU—clearly. Now, we've started moving abroad—but even here at home, we contradict each other. But I emphasize now—our position is: if you graduate someone in 2 or 3 years, we won't register them. That curriculum won't be approved. If it was approved in the past—it's gone. But now, we're no longer approving that kind of curriculum.

Interviewer: What specific national issues would you recommend be included in the development of master's degree programs in nursing, since Kairuki is planning to introduce master's programs? So, we need to understand what important aspects we should add or include in the programs we're initiating.

Respondent: Programs at the master's level or…?

Interviewer: Yes, at the master's level.

Respondent: Aaaah, at the master's level, honestly, I would recommend this—you know nowadays you can't easily **demarcate** what was covered in the bachelor's level and what in the master's. So I'd say, if we're starting master's programs, we should **forecast** what they're for. But I would recommend they be more **clinically oriented**, so that the people acquiring them are really gaining competence. Also, subjects like **policy**—people should be taught those. Beyond clinical competence, they should be taught how the world works. Also, topics like the **use of technology**—because in the bachelor's, there may be too many topics and some are left out. Now that someone is specializing, let them gain those competences. For example, if someone is doing **critical care**, they should truly be able to provide critical care. They should be able to **assess** patients, **manipulate** ventilators, **monitor**, and **take action**. But also, on medication—we were talking about interpretation—they should now learn about **dosages** more deeply. Teaching shouldn't just be classroom-based anymore. They should get **intro** in class, but real learning happens in **clinical** areas. Let's copy others—because if the course is clinical, and they're specializing clinically, let's **reduce theory**. When entering the master's level, let's **forecast** more on **clinical skills**. That's my perspective.

Interviewer: There's this Master’s in Midwifery program being offered at Muhimbili—I'm not sure if you've interacted with its graduates. Are there any **gaps** you’ve observed that could be improved in the program?

Respondent: Actually, there are **gaps**. Gaps that we’re trying to address, even while we’re developing a **scope of practice**, but still there’s an issue. In this master’s in midwifery, you find that previously the highest midwifery training was **at diploma level**. So they learned many things, but **didn’t specialize**. Now, when entering the master’s level, I believe we need to **empower** these individuals more in **diagnostic knowledge**, but also in **technology use**. For example, I expect this master’s-level midwife to **use ultrasound**, to be a **midwife sonographer**. To know that, “This mother came to me, I examined her, I used the ultrasound and noticed something is off—let me refer her to an obstetrician.” That’s what we’re aiming for. There are also **manipulation procedures**. You’ll find a stakeholder coming to teach 2-3 people how to use vacuum, how to do advanced techniques in midwifery—but I expect **master’s graduates** to already **know those things**. Yet sometimes they don’t. You look at the **curriculum**, and those components aren’t there—either they were squeezed in but aren’t taught properly.

Interviewer: Although I already asked earlier—maybe you already touched on it—but I’m not sure if you addressed the recognition of master’s graduates in the Tanzanian public service.

Respondent: Master’s degree holders **weren’t recognized** before in the public structure. But now a **proposal** has been submitted to recognize them. The new structure we’ve submitted to the **Public Service Office** recognizes master’s-level nurses. But recognition is one thing—they must **demonstrate mastery**. Many master's holders just have “bla bla.” So in these curricula, let’s **ensure** that master’s students **actually master** their field. If you say someone has a **master’s**, they must **master** that field. Whether it’s **clinical midwifery**, let them be someone who can **diagnose**, **perform procedures**, **teach**, and be **consulted**.

Interviewer: There are communication challenges among nurses. Do you see a difference in communication between master’s or bachelor’s degree holders and diploma holders?

Respondent: Eeh, maybe let me say this. That’s why I said earlier—issues of **customer care** and **communication** must now be prioritized. We must **invest** in them heavily because most complaints from our clients are about **poor communication and customer care**. But I’ve also said—let’s **equip** them with proper **knowledge** and **practical experience**. Because sometimes their communication is poor simply because they lack **competence**. They have no competence, yet boast: “I have a master’s!” or “I have a degree!” but no real competence. That’s why I said, let’s **focus** on providing competence in the clinical area. Secondly, in teaching—students come from different colleges. I don’t know whether they’re told things like: “You’ll be bedside nurses,” “You’ll be managers.” That causes challenges. Especially for master’s students—they go study, then come back, and instead of showing **improvement** they bring back **rank fights**: “I have a master’s, so I must be this and that.” We’re saying—go and **demonstrate** your master’s level in the clinical area. When someone says “I have a master’s,” let people **see it** in action. When someone says “I’m a graduate nurse,” let that show in how they work. Otherwise, people will start asking, “Who’s the certificate holder?” and “Who’s the degree holder?” And this is all because of **lack of competence**. But I believe in **science**. I didn’t go straight to university—I

started with diploma. Then, when I later went to study a degree, I **personally noticed the difference**. The science I got there—the **anatomy**, **physiology**, **biochemistry**, **epidemiology**, **behavioral sciences**—that's when I realized, "Ah, I was missing all this." That's why my reasoning used to be flawed. So these subjects are **fundamental**. Another thing—nowadays there's a debate: should master's be 2 years? We want the student to also do **research**, but the time is not enough. So these master's programs—we should **re-evaluate**. If you want to teach it in 2 years, **stuff it** with skills and knowledge. But also allow a **year for research**. So I would advise anyone starting a master's to make it **3 years**. The first **2 years**—focus on real clinical skills and knowledge. The **third year**—dedicated to research. Then, when they graduate and go into clinical settings—they are fully cooked. And when they go into research, they've been busy doing it. But now, with mixing everything together—they finish, but **can't even do research**. They come to clinical practice and can't even conduct a basic operation to get results. Don't even know how to start. People don't even know how to write a basic **concept note**. That's why I say—the current time is **not enough**. Let's increase it.

Interviewer: Okay, finally, in general—what are the key things we should consider as we review our curriculum and initiate a Master of Science in Midwifery?

Respondent: Starting with the **master's in midwifery**, I recommend one—**increase the duration** of the program. Two years takes us back to where we can't even demarcate properly. Second, when teaching a master's, it should be **clinical-based**. All that theory? It's been taught enough. So now, focus on **clinical practice** and **competencies**. Because even at diploma or bachelor's, midwifery isn't covered deeply—it's mostly just **maternal health**. So now, in midwifery at master's level, let's focus more on **abnormal conditions**—because the **normal** ones are even covered at certificate level. Let's go into recognizing **abnormalities**. Also, consider **technology**—it should not be a foreign thing. I went to Japan—the midwife stays with her patient, assesses her, takes out a **portable ultrasound**, and sees that amniotic fluid is low. Then she refers the patient to the obstetrician. That's what we should be doing at master's level. For **undergraduate** level, I say—let's **invest more in skills**. Let's reduce the overcrowded curriculum. Like for example, on **medical conditions**, maybe we just choose **two from respiratory**, **two from abdominal**, and so on. Teach them the **principles** of how to care for a surgical patient, a respiratory patient, etc.—but **go deeper in clinical practice**. That's my view.

Interviewer: Thank you so much for your cooperation. Everything you've said will be addressed and put into use.

Respondent: Okay, okay—you're most welcome.

Policymaker #5

Interviewer: I am from Hubert Kairuki Memorial University. We have a program for Bachelor of Science in Nursing. We are in the process of reviewing our BScN curriculum. So, you as one of our stakeholders in nursing programs in Tanzania, Head of the Department of Training and Professional Development for nurses in Tanzania, Madam [Name]... I have a few questions which I've been asked by the School of Nursing to ask you in order to help improve our nursing curriculum.

Interviewer: Maybe let me begin—do you have any thoughts or recommendations regarding the courses offered at Hubert Kairuki University for Bachelor of Science in Nursing students?

Respondent: My recommendations or thoughts... I think Hubert Kairuki has its curriculum—I wish I had it here! The curriculum you're using to offer training—I think it's a bit outdated, because I think if it's not from 2010, then it's from before 2010, which I saw here. So, it means this curriculum is offering training in some areas that are behind the current times. So I feel that although maybe you're offering training, there are some things this curriculum is lacking due to being outdated. I'm not sure because I don't have it here—I would have reviewed it. Because right now we're talking about various trends, and I feel your curriculum hasn't included those things.

Interviewer: Okay, since you said our curriculum is a bit outdated, maybe it hasn't captured current things—what specific knowledge or skills would you recommend for a nursing degree to address the changes in technology and the diseases that are emerging nowadays? What are the key things we can add?

Respondent: Mmmmh, okay. You know, for example, in the curriculum we're discussing or preparing—and as I look at the previous curricula—we look at things that are *current issues, current trends*. We expect the curriculum to meet or incorporate those things. For example, if you look at healthcare services today, they are *more advanced*, and nurses are the ones involved in these services. For example, now we're talking about **dialysis**, we're talking about all these **communicable diseases** and their **management**, we have **infectious disease trends**, **advanced care using technological machines**—all of these, if you look at them, the nurse is the number one involved. So if they're not in the curriculum and haven't been covered, definitely when the nurse arrives at the workplace, they won't be able to deliver those services. So I think things like these should be looked at. But there are also **cross-cutting issues** that emerge which a nurse should also know. How will they know them? By covering them in the curriculum. When such issues arise, they can apply them. So this curriculum needs to be reviewed to see what things were lacking behind so they can be incorporated. And when we say we are preparing what kind of nurse through this curriculum, we should look at: in reality, what kind of Tanzanian nurse do we want to produce? We want to put them in a position where **nursing becomes self-dependent**. So we must design a curriculum that prepares a person who is **self-reliant**, in terms of **critical thinking**, **applied sciences**, and **applied knowledge and technology** in delivering all services in their area.

Interviewer: Okay. Now, along with dialysis, maybe ultrasound—how can we build our students' capacity to read lab results, interpret them? They may read and interpret and be taught and understand, but the ability to act—like, now they've received the results, but the patient cannot be helped until the doctor is called to prescribe. What if it's night? How do we build this capacity in our graduates?

Respondent: Mmmmh... First, as I said, right now we want the nurse to apply the concept of **self-dependence**, and at the degree level, they must apply this self-dependence by using the skills and knowledge they have learned—**basic skills and knowledge**. They have learned science and must be able to **apply it**. There's also **evidence-based practice**, issues of **research**, and even lab results are **evidence** for them. They can use those results to **apply a problem-solving approach** and make **decisions**. So, I wish the curriculum would prepare someone who can do **decision-making**. Use of decision-making, applied sciences, and arrive at **proper interventions**, so that they can treat the patient. The result should be that **patient safety** is ensured. Because our aim in care and intervention is to ensure patient safety. If someone applies the competences well, applies their science well, uses critical thinking well, and has evidence—it leads to outcomes and results that allow them to intervene. And when we talk about all of this—it's the **nursing process**. If we properly train them in the nursing process, they can do everything, place the patient at the center, and do what we expect a well-prepared nurse to do.

Interviewer: Okay. On the other side—how are these Bachelor of Science in Nursing graduates recognized in the Tanzanian public service system?

Respondent: Mmmmh... The current **scheme of service**, or the one that has been revised—I believe it includes recognition for these graduates, although it's still in process. But yes, it has included and recognized them. I believe, for example, now it recognizes them based on the **duration of study** they've completed, and has included them in terms of **salaries and benefits**—based on their **science background**, **training time**. So they are recognized. But it's still in process, and I can't say how far it has gone because that's not the responsibility of the regulatory body in terms of the scheme or service—but it has already been addressed.

Interviewer: And because we are also in the process of launching a **Master's program in Midwifery**, we were wondering—how are **Master's graduates** recognized under this scheme of service? Or is it still under review?

Respondent: The structure recognizes them, but what I know is that any development of a document or any program must **go through a process**, and I believe even you are undergoing a process involving **key stakeholders**. Not just to gather information, but even for creating—for example, matters of public service must be consulted, to see what kind of master's you are launching. Does it have **recognition**? What are its **implications** in the public service? That way, we can be advised and develop the program accordingly—with alignment to the information already submitted to the **scheme of service**. In the scheme, **master's is considered**, but what I want to emphasize here is that **curriculum development** must involve **stakeholders from public service** to ensure the need and its job placement is clear. Not starting a program only to come later and demand recognition—we need to avoid that. That's why often we find ourselves **complaining** after the fact. This process must start early—**needs analysis** will help you define

the **demand** clearly. If it's a real need, what does the government say? What does the public service say? Where is this person going to be placed? That's when you can feel **confident** in running the program. But if it doesn't go through that process, the result is producing **graduates who are not recognized**. Then we start using lots of effort to ask why they are not recognized—when the problem started back when we were **designing the training**. So first, it is important to ensure **all institutions** involved in recognizing this person—including **public service**—are **involved from the beginning**.

Interviewer: At Kairuki University, as I said, we have a plan to introduce a Master's program in Midwifery. Maybe from your knowledge, what things hinder or obstruct the establishment of a Master's in Midwifery in Tanzania? And for us as a university, what are the key things we should forecast when launching this Master's?

Respondent: You want to launch a Master's in what? Midwifery?

Interviewer: Yes, Master of Science in Midwifery.

Respondent: I think you've studied and seen that other institutions also have the same Master's program. We cannot say that, like, a university can be stopped from introducing it. But when a university is introducing a Master's, I think as a university, you must be clear on **what kind of person you want to produce**. That's the first thing—not just "I have a Master's" and then... curriculum... process... but the environment is not conducive to preparing this person to truly be competent and be comparable or placed in a clinical setting and operate at the same level as another Master's holder. I'm saying this because, why? The environment may not be conducive to giving them skills at the level where we can say, "this is a Master's level," and we can compare them to another Master's. Because we say a person with a Master's in midwifery should be able to work alongside someone else at their level—maybe a doctor in that field. They should be able to go **toe-to-toe**. When we talk about **evidence-based issues**, **advanced care**, they too should be present there offering their skills in helping that mother or baby **at that level**. They should be able to integrate services, integrate technology, integrate things at an advanced level to save the mother. It shouldn't be that I have a Master's, but if you put me in a clinical placement, I don't produce any output different from someone with a degree—or even a diploma. The result is I just sit and complain: "you don't recognize us"—but you're not recognized because your competencies or services **don't portray the level of qualification**. We would love to say, "there's a Master's" and **see the impact** of a Master's holder in nursing or midwifery **within the system**. That we see their output just as we see our counterparts in other professions. Now I ask myself—is it maybe the **duration of study**? Because if you look at others, they study for **three years**, for example, postgraduate doctors study for three years. And **intensively** it's clinical-based. Now if we look at ours—some curricula, when you check, you find **it's mostly theoretical**. So what kind of skills are we giving this person to position them as a specialist? Aren't we preparing an expert? An expert? Then we need to make sure the curriculum is not a **repetition** of what they studied at the basic level. The curriculum should **prepare them in clinical-based** areas, and they should be placed in **clinical areas**. With case studies, with whatever cases—so that we can have evidence: "Here is a Master's midwife!" You see? [laughs] I think you understand meee!

Interviewer: So by that you mean, if I understood you well, the duration—what do we do now…? So the time for the Master's program, should it be extended?

Respondent: Mmmmh, I thiink for me, I was trying to look at the **product** of Master's programs—maybe Master's in Nursing, Midwifery—I want to compare it in a clinical base to other Master's programs. The **independence**, the **output**, is not there. Either there's a lack of **competence** or **skills** to give them evidence or give them confidence. So this confidence depends on the **length of training** and the time spent learning. So even if I say we should add more time, I think it might not be the issue—because others in the same health field study Master's for **two years**, but there are some who study more. So we need to ask ourselves: why is it that our Master's graduates are not at the same level as theirs? When they come back, they come back to the same point—no difference. I don't know if you've already seen that, mmmmh...

Interviewer: Okay. Maybe now that we're reaching the end—when it comes to nurses—there is the issue of **communication** between nurse and nurse, communication between nurse and patient, and also the **use of information and technology**. How do you speak about this issue with nurses? Because there are some murmurs that maybe the language is not friendly.

Respondent: First of all, yaaah... the issue of communication, if you speak about it, is very broad. But the **key**, or the **pivotal area** which we should consider very seriously in **discharging our services**, is **improving communication**. And communication is **everything**. When you start talking about **professionalism**, **ethical practice**, **behavior**, all those demonstrations and behaviors—it's all communication. Even when a person speaks, or when they meet a patient, when they face the patient—that's all communication. There's a problem there. We really see that most complaints—many of them—the source is **communication**, whether verbal, whether documentation, whether reporting, whether informing—it's all areas where we've **lagged behind**. If we don't provide proper information—that's communication. Documentation? That's communication. The way you behave? You're communicating. All of that is communication. So there must be **a strong foundation built** so we can improve communication among our professionals.

Interviewer: Sorry, you said we should build their capacity properly—so how do we **build enough capacity** for our graduates?

Respondent: In this, I think, in teaching—there are **modes** for teaching communication. But I don't know what to say—**role modeling**, first of all. But also giving them **exposure to proper communication**, and **practice** in implementing the **knowledge, skills, and attitudes** of communication. Maybe these things are not being acquired enough—maybe our curriculum **doesn't allocate enough time** for people to learn. But also, **role modeling**—we need an environment where this person can demonstrate proper communication skills and apply them wherever they are providing services. That is one of the **key areas of competence**, and I think we need to **emphasize it strongly**, because it cuts across all other areas. If we have enough time—and also the issue of **ethics**, yes. **Professionalism and ethics**—there's a problem. There's a problem because **morals are deteriorating** every day. Now we ask: this module, or the contents of professionalism and ethics, how are they being handled? Mmmmh, you know we're

admitting youth into the BScN program. But **modeling** must be done properly, by emphasizing these **moral principles**. These are what carry everything else in the curriculum. Mmmh.

Interviewer: Okay. Lastly, as we go to review our curriculum—what are the **top priorities** that you, as the Head of the Department of Training and Academic Development, feel are the **most essential things** we should consider—now that we've discussed many things?

Respondent:
Mmmmhh, I think I've said many things in the conversation there—you can see them—and the main thing is that issue of **ethics**, matters of **communication**, the issue of **professionalism**, because all those, one will get. The others—those **applied sciences**—he/she will get. But the **foundations**, especially the foundation of **nursing**, which is what we say is professionalism—the foundation of nursing must be considered. But more importantly, **enough time in clinical** [laughing]—that is the main thing we would like you to look into. This person should get enough time—this nurse, when he/she is... when he/she doesn't get enough clinical time, so that he/she can **integrate what he/she has learned** in... in knowledge—should get enough **skills**, should get enough **exposure**. Mmmmh, **enough clinical time** is very fundamental, and those are the things we need to look into. Right now, we are using **competence-based** [curriculum]. I'm not sure how the **skills lab** preparation is, but I think the **clinical area** is the best—we should allocate enough **clinical time** in our curricula. Mmmmh, that's where one will get everything. Mmm. But the main thing is: that clinical area—**they must have enough time**, but also **adequate supervision and support** from the teachers who taught them in class, to go with the student into clinical [practice]. This is a **big challenge** in curriculum implementation. So even when you're thinking of preparing a curriculum—even for this **Master's**—you must **raise the challenges** that are said to impair... impair the Master's. It's the **number of teaching staff**, the **support for students**. When we say this student should be **clinical-based**, what support do they get **behind the scenes** to help them become a competent person? We can give them a lot of time, but if they **don't have support**, then that's... those are the things to think about—adequate **resources** in the clinical setting to support them, and from the school, to support the student. Mmmmh. Because the curriculum is not just for the trainers—it also depends on the **areas to implement that curriculum**. If those are missing, there's nothing—we may design a very good curriculum, theory and everything, but the **implementation** requires **adequate support**. Mmmhh.

Interviewer:
Then thank you so much, thank you for your time and for your cooperation. All that we've gathered we will go and work on it.

Respondent:
Thank you very much.

Interviewer:
Thank you very much, may God bless you.

Respondent:
You're very welcome.
